# Sterically constrained tricyclic phosphine: redox behaviour, reductive and oxidative cleavage of P–C bonds, generation of a dilithium phosphaindole as a promising synthon in phosphine chemistry†‡

**DOI:** 10.1039/d0sc06155g

**Published:** 2021-01-18

**Authors:** Alexander Brand, Stephen Schulz, Alexander Hepp, Jan J. Weigand, Werner Uhl

**Affiliations:** Institut für Anorganische und Analytische Chemie der Universität Münster Corrensstraße 30 D-48149 Münster Germany uhlw@uni-muenster.de; Anorganische Molekülchemie, Fakultät für Chemie und Lebensmittelchemie, Technische-Universität Dresden D-01069 Dresden Germany jan.weigand@tu-dresden.de

## Abstract

The redox behaviour of sterically constrained tricyclic phosphine **3a** was investigated by spectroelectrochemistry. The data suggested a highly negative reduction potential with the reversible formation of a dianionic species. Accordingly, **3a** reacted with two equivalents of Li/naphthalene by reductive cleavage of a P–C bond of one of the PC_4_ heterocycles. The resulting dilithium compound **5** represents a phosphaindole derivative with annulated aromatic C_6_ and PC_4_ rings. It is an interesting starting material for the synthesis of new heterocyclic molecules, as was shown by treatment with Me_2_SiCl_2_ and PhPCl_2_. The structures of the products (**6** and **7**) formally reflect ring expansion by insertion of silylen or phosphinidene fragments into a P–C bond of **3a**. Treatment of **3a** with H_2_O_2_ did not result in the usually observed transfer of a single O atom to phosphorus, but oxidative cleavage of a strained PC_4_ ring afforded a bicyclic phosphinic acid, R_2_PO_2_H.

## Abstract 2

Sterically constrained tricyclic phosphines with annulated five- and six-membered rings show fascinating chemical and redox reactivity as indicated by sophisticated *in situ* UV-vis CV and multi-pulse chronoamperometry.

## Graphical abstract

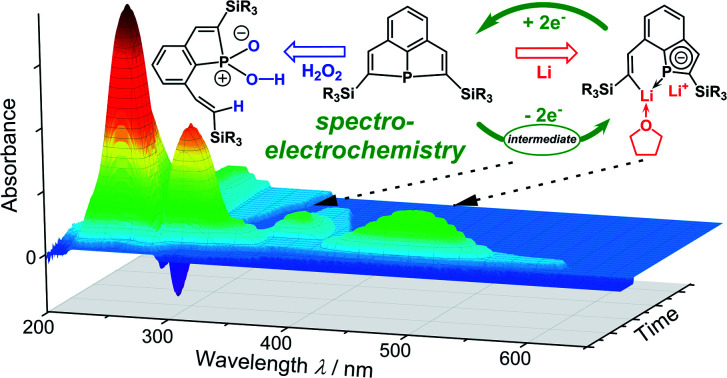

## Introduction

**Scheme 1:**
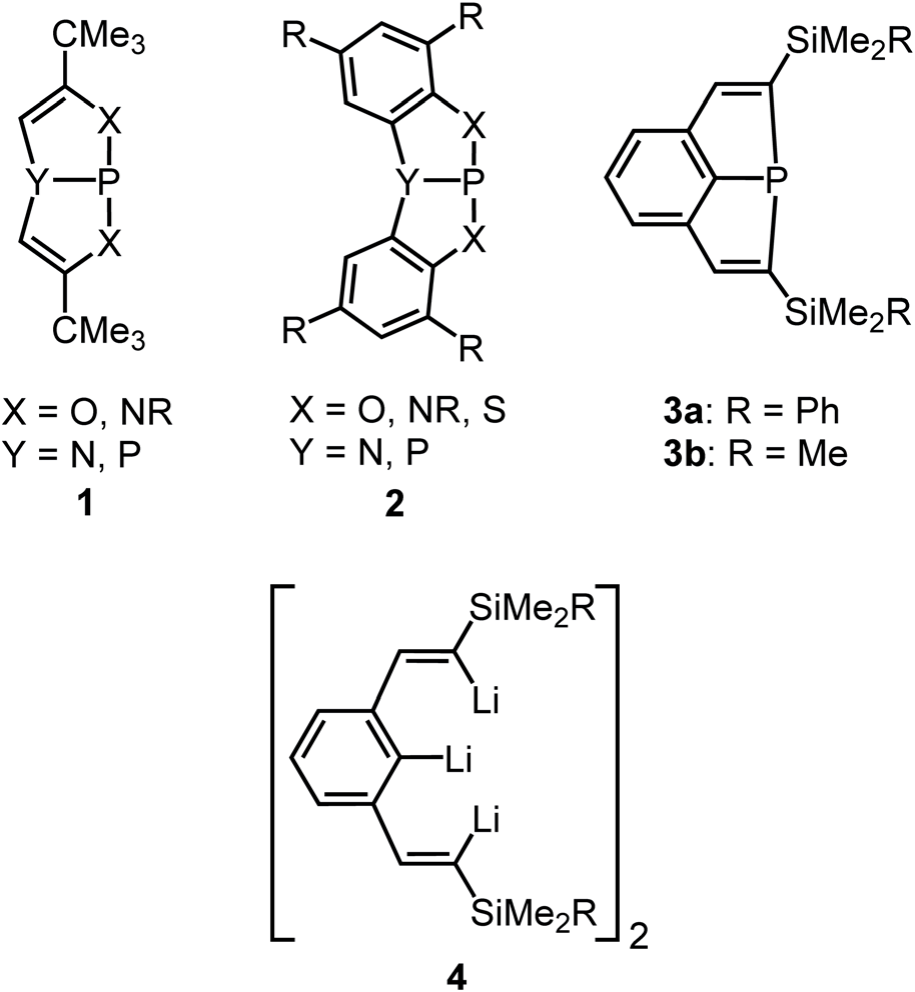
Sterically constrained bi- and tricyclic phosphines (**1–3**) and the trilithium starting compound (**4**).

The highly interesting chemical properties of sterically constrained bicyclic or tricyclic phosphines (**1** to **3**; Scheme 1) have attracted considerable attention in recent research.^1–7^ These compounds are accessible through facile routes and have in most cases heteroatoms such as N, O, S or P bound to their central P atoms. Only recently we found facile access to the first derivatives (**3**) which had the P atoms coordinated in a homoleptic fashion by three C atoms.^1,7,8^ The latter compounds were obtained by treatment of the dimeric or trimeric trilithium compounds **4** (ref. 7 and 9) with PCl_3_ and salt elimination. The steric strain of these molecules became evident from flattened molecular structures with unusually large C_vinyl_–P–C_vinyl_ angles of about 128°, very short P–C bonds to the ipso-C atoms of the bridging aryl rings (∼175 pm) and a considerable deviation of the α-C atoms of the vinyl groups from the planes of the central aryl rings. All phosphines **1** to **3** show a fascinating chemical reactivity which comprises normal phosphine reactions such as oxidative addition to the P atoms or formation of transition metal complexes by retention of the molecular constitution.^1^ The first type of reactions^2–5,7,8,10,11^ afforded *e.g.* dihalogenophosphoranes, which allowed the synthesis of unusual dimethyl substituted phosphor(v) species.^2*a*,8^ More fascinating are reactions which are clearly favoured by the minimization of ring strain and result in ring cleavage or insertion of atoms into endocyclic P–X bonds by ring expansion. Such transformations comprise the activation of H–H,^12^ O–H^2,4^ or N–H bonds^5^ and the insertion of O,^10*a*^ S^7^ and Se atoms^7^ or of CO molecules.^13^ Ring strain should also result in a unique and highly promising redox behaviour of such compounds.

Not only electron precise species may be formed, but one-electron reduction or oxidation may result in the formation of radical species which may be stabilized by delocalisation of electron density into the molecular backbone. However, investigations into the redox chemistry of **1** to **2** are limited and have not been elucidated systematically. Treatment with a strong reductant (KC_8_) resulted in P–P bond formation,^10*a*^ while oxidation with silver cations Ag^+^ afforded a relatively persistent radical cation.^14^ The stable and nonpolar P–C bonds of compounds **3** may help to stabilize unusual products of electron transfer reactions. The constitution of **3** may allow the facile delocalisation of spin density or charges into the π-systems of adjacent aromatic rings and alkenyl moieties and contribute to the stability and isolability of reactive secondary products. In this article we report on comprehensive studies into the redox properties of phosphine **3a** by cyclovoltammetry combined with spectroelectrochemistry and on first successful attempts to transfer the results into a preparative scale.

## Results and discussion

### Cyclic voltammetry and square-wave voltammetry of phosphine **3a**

Analytical cyclic voltammetry (CV) was used to elucidate the electrochemical behaviour of tricyclic phosphine **3a**. Based on the sterically constrained structure of phosphine **3a** oxidation reactions may lead to the formation of kinetically stabilized radical cations,^15^ while reductions are expected to result in P–C bond cleavage^16^ driven by release of ring strain. *In situ* UV-vis-spectroelectrochemical methods like UV-vis CV as well as UV-vis multi pulse chrono amperommetry (*in situ* UV-vis MPCA) in semi-preparative measurement cells were used to explore the stability of formed species and to probe for preparative accessible compounds. CV of phosphine **3a** at a platinum disc electrode was optimized in fluorobenzene (see ESI Fig. SI.1.‡), acetonitrile (see ESI Fig. SI.2.–4.‡) and tetrahydrofuran (see ESI Fig. SI.5. and 6.‡) as solvents. A low diffusion coefficient and, therefore, low intense and broad peaks in the CV hampered an use of electrochemically reductive stable fluorobenzene as an electrolyte. Acetonitrile enabled the determination of the oxidation (*E*_1/2_(SWV) = 1.15 V)^17^ and reduction potential (*E*_1/2_(SWV) = −2.38 V)^17^ but also showed follow-up reactions of the oxidized (ESI Fig. SI.3.3‡) and reduced (ESI Fig. SI.3.4‡) species. Tetrahydrofuran represents a good alternative with a wider potential window for the reduction reaction, while allowing for an acceptable peak shape due to the diffusion coefficient of phosphine **3a**. Oxidation of neutral phosphine **3a** does not lead to stable (PhF, CH_3_CN) or accessible (THF) species.

**Fig. 1:**
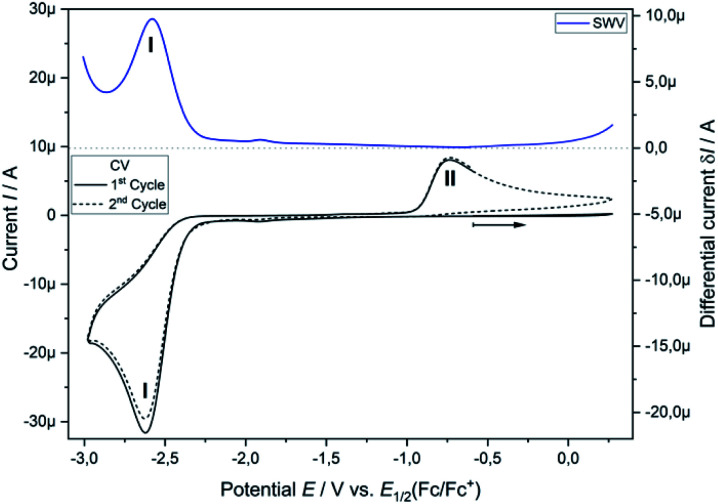
Cyclic voltammogram of **3a** (2.32 mM) in THF/0.1 M [^*n*^Bu_4_N][OTf] at a Pt disc electrode (1.6 mm; 0.1 V s^−1^). *iR* compensated by *R* = 4750 Ω.

Cyclic voltammetry of phosphine **3a** in THF with [^*n*^Bu_4_N][OTf] as a supporting electrolyte shows a non-reversible reduction process at a very low peak-potential of *E*_P_(red **I**) = −2.63 V (*vs. E*_1/2_(Cp_2_Fe/Cp_2_Fe^+^)) in the first cycle (Fig. 1). The second cycle reveals a non-reversible re-oxidation process at *E*_P_(re-ox **II**) = −0.74 V in a potential region where the starting material **3a** was electrochemically not active. Square-wave voltammetry (SWV) allowed to assign a half-wave potential of *E*_1/2_ = −2.58 V for the substrate-based reduction reaction **I**, while the re-oxidation process **II** is not being found in accordance with the necessity of the formation of the product from the reduction **I**. *In situ* UV-vis CV spectroelectrochemistry was used to gain mechanistic insights for the reduction **I** of **3a** and the re-oxidation **II** of the product of the reduction **I**.

### *In situ* UV-vis-NIR spectroelectrochemistry of phosphine **3a**

**Fig. 2:**
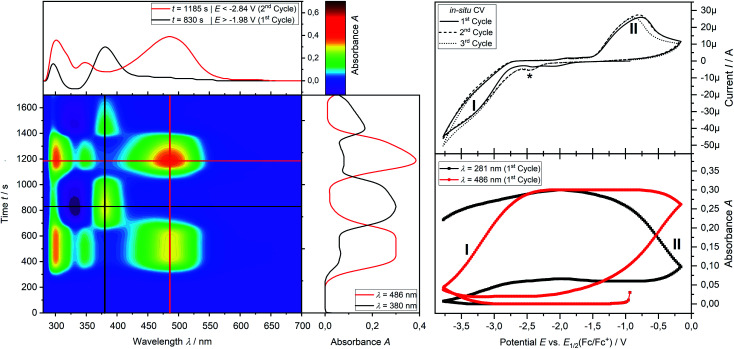
Spectroelectrochemical CV measurement of the reduction **I** and reoxidation **II** (*v* = −10 mV s^−1^) of phosphine **3a** (2.50 mM) in THF/0.1 M [^*n*^Bu_4_N][OTf] at a platinum grid electrode in a double compartment cuvette cell. Left contour plot: relative UV-vis spectral changes during 3 cycles of CV; middle vertical plot: time dependent UV-vis absorption; top left: projected relative UV-vis spectra after reduction **I** (red line) and after reoxidation **II** (black line); top right: *in situ* CV, bottom right: absorption based CV.

**Fig. 3:**
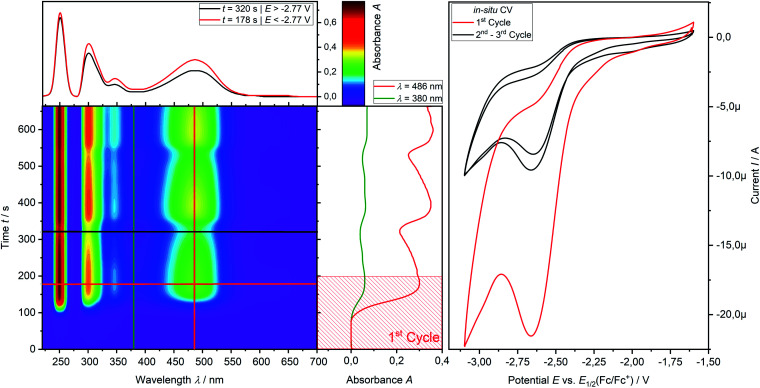
Spectroelectrochemical UV-vis CV (*v* = −15 mV s^−1^) measurement of the reduction **I** of phosphine **3a** (1.00 mM) in THF/0.1 M [^*n*^Bu_4_N][OTf] at a platinum grid electrode in double compartment cuvette cell. Left contour plot: relative UV-vis spectral changes during 3 cycles of CV; middle vertical plot: time dependent relative UV-vis absorbance; top left: projected relative UV-vis spectra after reduction in the 1^st^ CV cycle (red line) and after the 2^nd^ CV cycle (black line); right: *in situ* CV.

**Scheme 2:**
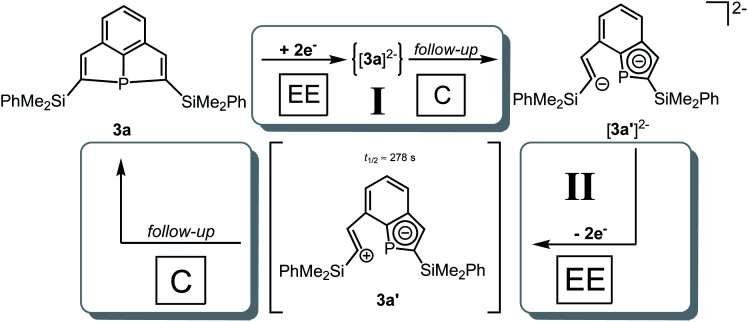
Mechanism for the two-electron reduction **I** of tricyclic phosphine **3a** to anion **[3a′]2−** by an EEC mechanism und re-oxidation **II** mechanism *via* an EEC sequence involving a meta-stable intermediate **3a′**.

**Fig. 4:**
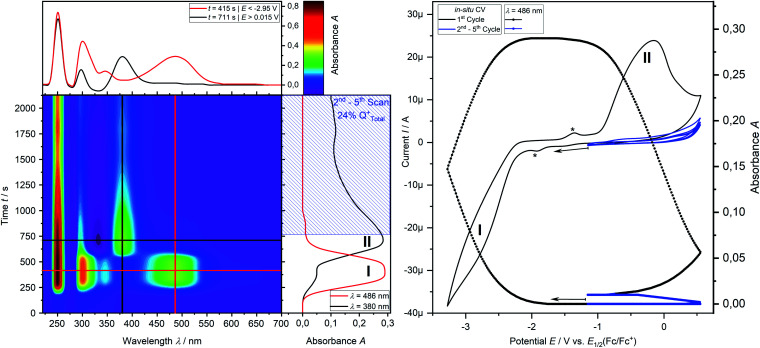
Spectroelectrochemical CV measurement of the reduction **I** and exhaustive re-oxidation **II** (*v* = −10 mV s^−1^) of phosphine **3a** (2.50 mM) in THF/0.1 M [^*n*^Bu_4_N][OTf] at a platinum grid electrode in a double compartment cuvette-cell. Left contoure plot: relative UV-vis spectral changes during 5 cycles of CV; middle vertical plot: time dependent relative UV-vis absorption; top left: projected relative UV-vis spectra after reduction **I** cycle and multiple reoxidation **II** cycles; top right: *in situ* CV; bottom right: absorption based CV.

CV measurement of phosphine **3a** in THF with *in situ* UV-vis monitoring under optimized conditions in double-compartment cuvette-cell lead to a CV curve (Fig. 2, right) comparable to the conventional CV (Fig. 1). *In situ* UV-vis spectroscopy required a high concentration of phosphine **3a** by compromising peak shapes (see ESI Fig. SI.7.‡ for a low concentration measurement, comparable to Fig. 1). The UV-vis spectrum (Fig. 2, red horizontal line) of the reduction product **I** shows a visible absorption maximum at *λ* = 486 nm. At the re-oxidation peak **II** the UV-vis spectrum indicates a new product with an absorption at *λ* = 380 nm decreasing (Fig. 2, black horizontal line) till the reduction **I** potential is reached in the second cycle again. The absorption-based CV curve intensity for the product of the reduction **I** (red line, bottom right) increases at the reduction **I** but also rapidly decreases at the re-oxidation **II**. This finding indicates a spectroscopically reversible reduction of phosphine **3a** to a reduction product which is formed back at the re-oxidation **II**. An intermediate of the re-oxidation **II** (black line) is evolving at the re-oxidation **II** and decreases slowly under formation of the phosphine **3a**. *In situ* UV-vis CV is executed solely for the reduction reaction **I** in order to investigate the stability of the reduction product. Fig. 3 shows the formation of the product of reaction **I** as indicated by the UV-vis spectrum (red horizontal line) and the intensive peak **I** in the first CV cycle. Further CV cycles around the reduction process **I** indicates no additional signs of decay (UV-vis, black line). Absorbance fluctuations after the first cycle (red line, vertical) are caused by diffusion from the thin-layer to the bulk area of the cuvette-cell. Based on the SEC results an electrode reaction mechanism (Scheme 2) is proposed. The electrochemically non-reversible reduction reaction **I** may lead to the formation of unstable intermediate [**3a**]^2−^ by a two-electron reduction (*vide infra*) which than reacts under P–C bond cleavage to the dianion **[3a′]2−**. The electron transfer with its follow-up reaction is best described by an EEC mechanism scheme. Comparing the peak potentials of the re-oxidation process **II** (*E*_P_ = −0.74 V) to the peak potential reported for the oxidation of vinyl lithium (*E*_P_ = −0.06 V)^18^ and benzophospholide lithium salts (*E*_p_ ≈ 1.19 V)^19^ results in the assumption of a two-electron (*vide supra*) reduction thus leading to the intermediate **3a′** with a preserved phospholide moiety and a α-silyl vinyl cation by two-electron reduction. In a homogenous follow-up reaction intermediate **3a′** is undergoing a ring-closure reaction forming phosphine **3a** back in accordance to the spectroscopic reversibility observed for the full process. To proof the two-electron transfer steps followed by a slow chemical follow-up reaction as an EEC mechanism a further spectroelectrochemical proof of the ring-closure reaction without an electron transfer involved has been realized. Fig. 4 shows an *in situ* UV-vis CV with a first cycle fully converting phosphine **3a** by the reduction **I***via* an EEC mechanism to the stable dianion **[3a′]2−** (red horizontal spectrum). Due to the slow scan rate the concentration of the dianion **[3a′]2−** and, therefore, its absorption becomes steady in time dependent (red vertical line) and potential dependent representation (black line, right diagram). Potential sweeps around the re-oxidation **II** range fully converts dianion **[3a′]2−** to the intermediate **3a′** based on the decreasing absorption of dianion **[3a′]2−**. Within the 2^nd^ to 5^th^ cycle no significant current is observed in the CV. The UV-vis spectrum shows the characteristic absorptions of intermediate **3a′** (black horizontal spectrum) decreasing over time (blue shaded area) by the chemical follow-up reaction forming phosphine **3a**. A half-life time determination (ESI, Fig. S8.‡) for the ring-closure reaction to form phosphine **3a** was performed giving *t*_1/2_ = 278 s regarding the intermediate **3a′**.

**Fig. 5:**
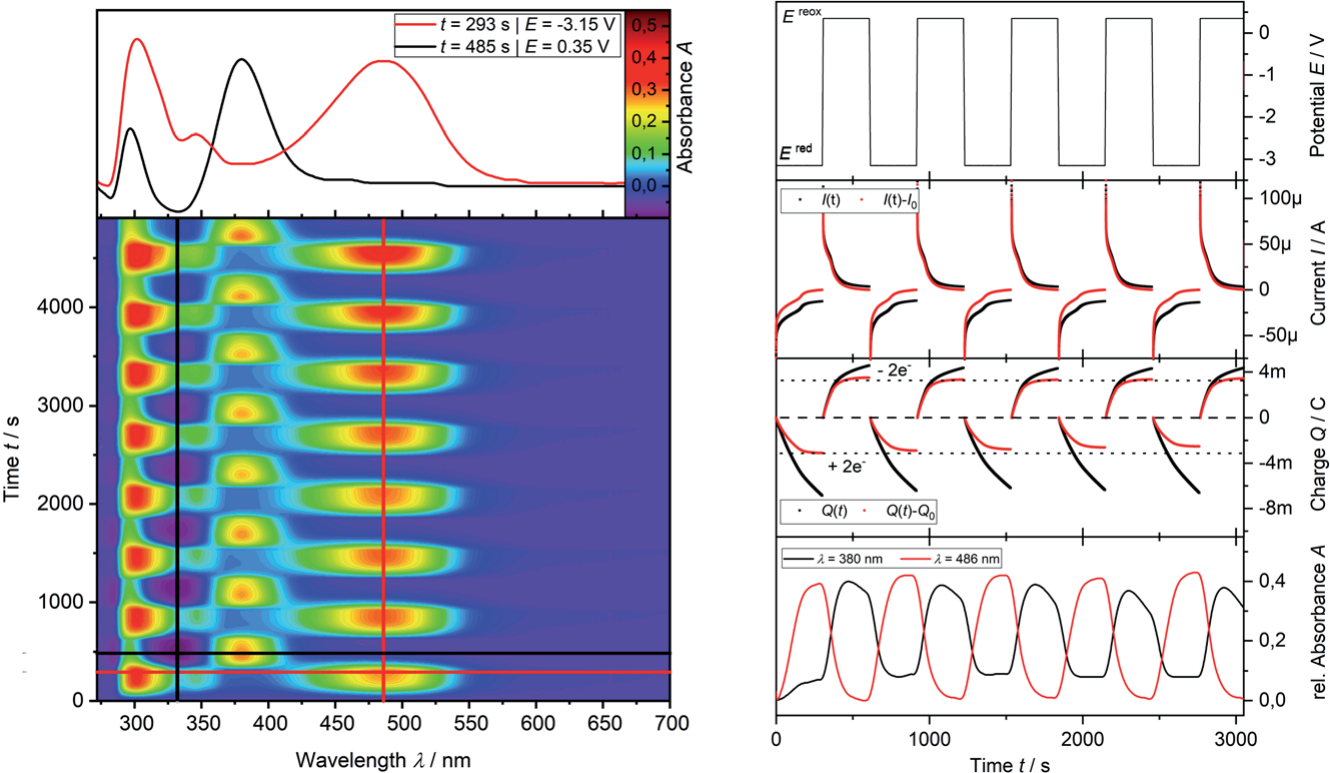
*In situ* UV-vis multi pulse chrono amperommetry (*in situ* UV-vis MPCA) of phosphine **3a** (2.50 mM) in THF/0.1 M [^*n*^Bu_4_N][OTf] at a platinum grid electrode in a double compartment cuvette-cell (*d* = 0.5 mm). Left, bottom: 2D-plot of relative *in situ* UV-vis spectra during the MPCA measurement (reduction **I** and re-oxidation **II** pulses); left, top: relative UV-vis spectra of the reduction product **[3a′]2−** (red line) and the intermediate **3a′** (black line) of the re-oxidation; right top to bottom: potential profile, chonoamperogramm, chronocoulogramm, chronoabsorptiometry.

Finally, the number of electrons involved in the EEC processes concerning the reduction **I** of phosphine **3a** to dianion **[3a′]2−** and its re-oxidation **II** is determined by the use of *in situ* UV-vis multi pulse amperommetry (Fig. 5). Applying repetitive reduction and re-oxidation pulses (1^st^ diagram Fig. 5) in a calibrated volume double-compartment cuvette-cell allows for the determination of the number of electrons consumed from the chronocoulogram (3^rd^ diagram Fig. 5). Multiple reversible switching between phosphine **3a** and dianion **[3a′]2−** (red line, 4^th^ diagram Fig. 5) over intermediate **3a′** (black line, 4^th^ diagram Fig. 5) further proved the reversibility as well as the two-electron nature of the process after correction of diffusion effects (compare ESI Fig. S9.‡).

The redox properties of tricyclic phosphine **3a** does not allow to access a product of its oxidation, while the reduction reaction at highly negative potentials leads to the formation of stable dianion **[3a′]2−**. *In situ* UV-vis CV and MPCA reveals a two-electron process for the reduction *via* an EEC sequence without a detectable intermediate (CV at 10 V s^−1^, ESI Fig. S10.‡) pointing towards a fast, chemical follow-up reaction to form dianion **[3a′]2−**. Furthermore, it was demonstrated that the reduction sequence from this dianion is chemically reversible proceeding over a metastable intermediate **[3a′]2−** after the two-electron reduction (EE part) with a slow follow-up reaction (C part) back to phosphine **3a***via* a total EEC sequence. Therefore, suitable reduction reagents should allow for preparative synthesis of dianion **[3a′]2−** while the intermediate **3a′** is only accessible *in situ*.

### Reductive ring cleavage with the tricyclic phosphine **3a**

**Scheme 3:**
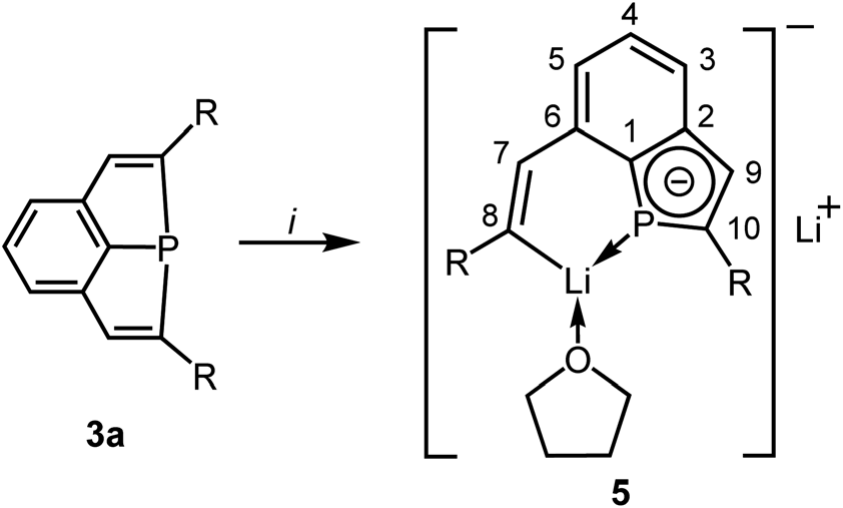
Synthesis of **5** [*i*: 2 Li, 2C_10_H_8_, THF; R = SiMe_2_Ph]; numbering scheme used for assignment of NMR data.

Electrochemical investigations revealed a very high negative potential necessary for the reduction of the tricyclic phosphine **3a** and suggested a challenging search for suitable reductants, which would allow the transfer of these results into a preparative scale. Treatment of **3a** with potassium graphite afforded dark red mixtures. NMR spectra were unspecific and showed only broad and low intensity resonances, which did not allow a reasonable assignment. All attempts to isolate a crystalline material by recrystallization of the crude product from various organic solvents failed (Electrosynthesis: see ESI Chapter 1.4‡). Metal naphthalenides are strong reducing agents and have potentials of about −3.0 V.^20^ We, therefore, treated **3a** with *in situ* prepared Li naphthalenide in a molar ratio of 1 : 2 in THF at −78 °C (Scheme 3). Slow warming to room temperature, filtration, evaporation and recrystallization of the solid residue from toluene afforded yellow crystals of compound **5** in 70% yield. Crystal structure determination (see below) confirmed the reductive cleavage of a P–C bond of one of the five-membered PC_4_ heterocycles by transfer of electrons to the C atom of the C–SiMe_2_Ph group and to the P atom.

Once crystallized, the solubility of **5** in toluene or benzene decreased dramatically. The NMR spectra were recorded in THF. The P atom of **5** shows a signal at *δ* = 45.1 ppm in the ^31^P NMR spectrum, which is shifted to a higher field compared to that of **3a** (*δ* = 68.8 ppm). The typical range of lithium phosphides is at about −120 ppm.^21^ The ^3^*J*_PH_ coupling constant to the H atom attached to the intact ring is comparatively small (11.3 Hz *versus* 18.3 Hz in **3a**) and may indicate a reduced *s*-character in the P–C bonding orbitals in accordance with Bent's rules^22^ or a reduced bond order of the C=C bond. The usual chemical shift of *δ* = 7.47 ppm is observed for this H atom, while the signal of the vinylic H atom at the opened ring (H–C7) in β-position to Li is shifted to a lower field (*δ* = 9.02 ppm). The latter value corresponds to that of the vinylic H atoms in the trilithium compounds **4**.^7^ The C atoms attached to P have ^13^C NMR signals at *δ* = 145.1 (C10) and 150.1 (C1) with ^1^*J*_PC_ coupling constants of 54.0 and 34.6 Hz. Also the remaining two C atoms of the PC_4_ ring show resonances [*δ* = 144.8 ppm (C2) and 128.1 ppm (C9)] similar to those of the annulated, aromatic C_6_ ring. The resonance of the carbanionic C atom C8 is shifted to a lower field (*δ* = 198.5 ppm) and similar to the chemical shift observed for **4** (*δ* = 192.1 ppm). The coupling constant of C8 to the P atom is surprisingly large with 34.5 Hz. Only a broad resonance was detected in the ^7^Li NMR spectrum at *δ* = 1.0 ppm.

**Fig. 6:**
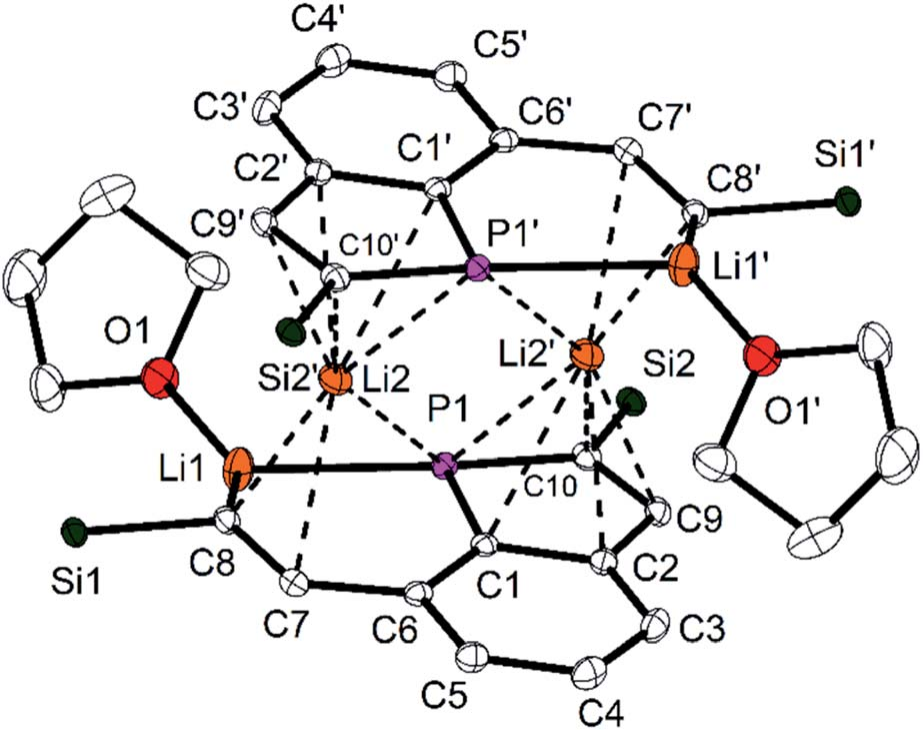
Molecular structure and numbering scheme of **5**. Displacement ellipsoids are drawn at the 40% level; *T* = 100 K. H atoms and the SiMe_2_Ph substituents (with exception of the Si atoms) are omitted. Dashed lines indicate short distances between Li2 and atoms of the phosphine backbones. Important bond lengths (pm) and angles (°): Li1–P1 246.8(3), Li1–O1 187.1(4), Li1–C8 210.9(4), Li2–P1 277.9(3), Li2–P1′ 261.6(3), Li2–C1 277.1(4), Li2–C1′ 257.0(4), Li2–C2′ 247.0(4), Li2–C9′ 235.4(4), Li2–C10′ 236.7(4), Li2–C7 249.5(4), Li2–C8 217.4(4), P1–C1 178.3(2), P1–C10 178.8(2), C1–P1–C10 91.45(8); symmetry equivalent atoms generated by −*x* + 1, −*y* + 1, −*z* + 2.

**5** forms centrosymmetric dimers in the solid state (Fig. 6) with the Li atoms Li2 and Li2′ in the bridging positions of a double sandwich type molecule. The C=C distance in the vinylic group of the opened ring (C7–C8) is with 135.6(2) pm in the typical range of C=C double bonds. The C–C distances in the PC_4_ ring are longer [138.7(3) (C9–C10) to 143.4(2) pm (C1–C2)] and similar to the remaining ones of the annulated aromatic ring [137.1(3) and 142.5(3) pm]. The P–C bond lengths are similar [178.3(2) and 178.8(2) pm] and shorter than usually observed for P–C single bonds. The PC_4_ ring is almost planar with the maximum deviation of 0.3 pm for the atom C9 and is coplanar to the C_6_ ring (angle between the normals of the planes 2.6°). These structural parameters resemble those of phospholyl anions^23–25^ with delocalized π-electrons. The NICS(0) value^23*k*^ of **5** was calculated for the optimized structure (HF/def2-TZVP//PBE0/def2-TZVP)^26^ to −11.44, it confirms the aromatic character of the PC_4_ ring and is quite similar to values of related phospholyl anions reported in the literature.^23*l*,*m*^ Hence, reduction of the tricyclic phosphine resulted in an unexpected structural motif which may best be compared to that of phosphaindoles. Two comparable compounds have been reported, in which the five-membered heterocycles were coordinated to Li or transition metal atoms.^25^ Our interpretation is supported by the results of ^31^P and ^13^C NMR spectra, in particular the ^31^P NMR chemical shift is in the typical range.^23–25^ Similar to the few lithium phospholides reported in the literature, the Li atom Li2 has relatively short contacts to all four C atoms of the intact PC_4_ ring [Li2–C9′ 235.4(4) to Li2–C1′ 257.0(4) pm] and to P1′ [261.6(3) pm]. Li2 is further coordinated to three atoms of the opened ring in the second molecular half to form the dimeric formula unit. The relevant distances are 277.9(3) pm (Li2–P1), 217.4(4) pm (Li2–C8) and 249.5(4) (Li2–C7); the other Li–C distances are at least 25 pm longer. The atoms Li2, Li2′, P1 and P1′ form a Li_2_P_2_ ring. The second Li atom (Li1) has coordination number three [Li1–O1 187.1(4); Li1–C8 210.9(4); Li1–P1 246.8(3) pm] in an almost ideal planar surrounding (sum of the angles 350.9°). The Li–P distance compares well to values reported for lithium phosphides.^27^ The η^1^-coordination of the P atom of a phospholyl anion to Li has not been observed previously. Cleavage of P–C bonds by Li is known for phenyl- or allylphosphines,^28^ but **5** is the only isolated compound, which has the resulting anionic components combined in a single molecule.^24^

### Reactions of the dilithium compound with element dihalides

**Scheme 4:**
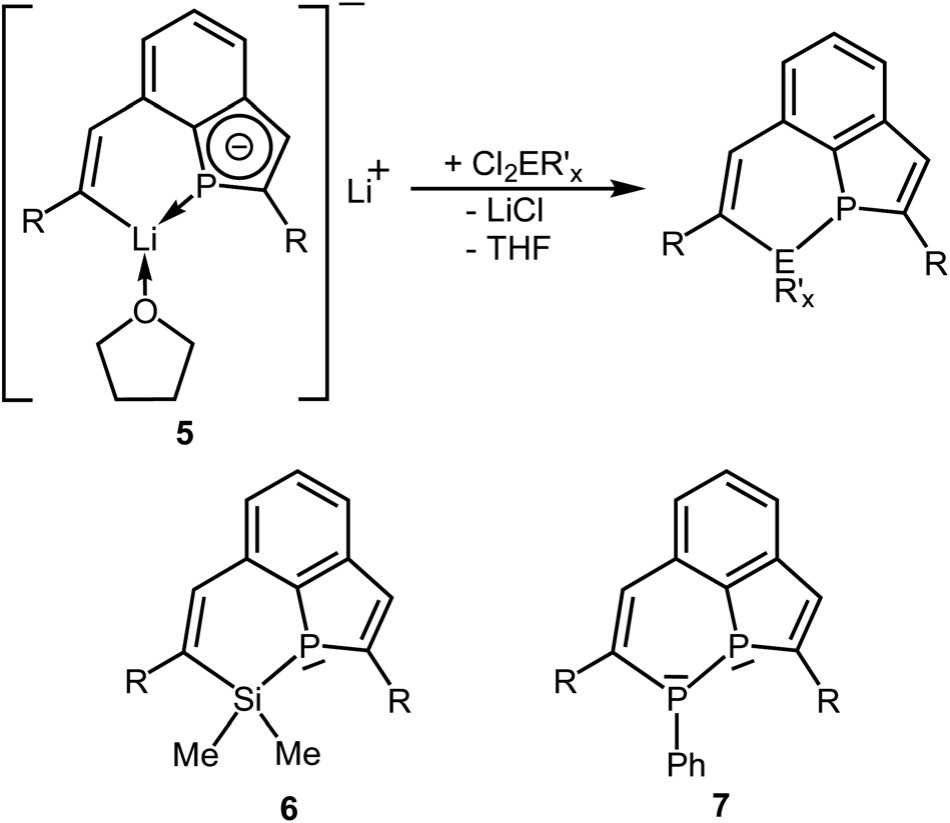
Reactions of **5** with 
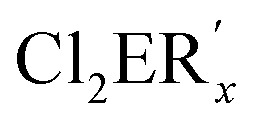
 (E = Si, P; R′ = Me, Ph; R = SiMe_2_Ph; *x* = 2 or 1) and schematic drawings of **6** and **7**.

The dilithium phosphaindol **5** is a highly interesting starting material for the synthesis of various secondary products by salt elimination. As a proof of principle we treated it with Me_2_SiCl_2_ and PhPCl_2_, in order to obtain new tricyclic compounds with a heteroatom embedded in one of the rings. The proposed transformations correspond formally to a ring expansion of the starting phosphine **3a**. THF solutions of isolated or *in situ* generated samples of **5** were treated with the neat element dihalides at room temperature or at −78 °C (Scheme 4). All volatiles were removed in vacuum at room temperature, the residues were extracted with *n*-pentane or *n*-hexane, and the resulting suspensions were filtered. Removal of the solvent from the filtrate afforded the Si compound **6** as a colorless, viscous liquid, which could not be obtained as a solid material from various solvents, while the diphosphorus compound **7** crystallized after concentration of the filtrate at room temperature and was isolated as yellow crystals in 85% yield.

**Fig. 7:**
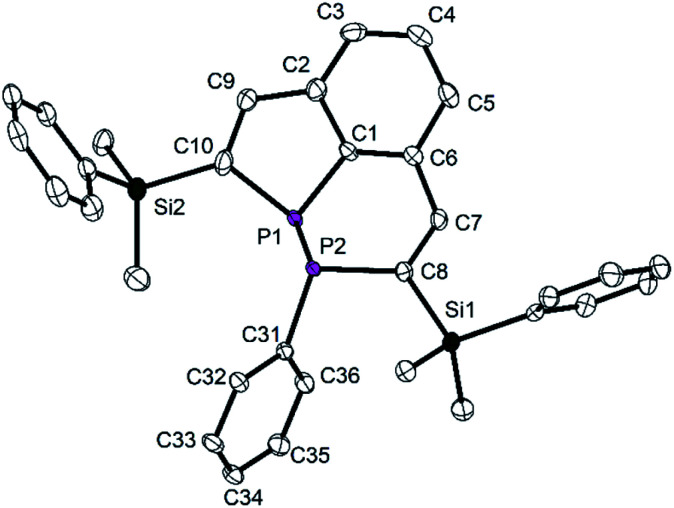
Molecular structure and numbering scheme of **7**. Displacement ellipsoids are drawn at the 40% level; *T* = 100 K. H atoms are omitted. Important bond lengths (pm) and angles (°) (main component; see text): P1–P2 219.59(9), P1–C1 179.8(3), P1–C10 184.1(2), P2–C8 185.3(8), P2–C31 184.0(3), C7–C8 134.3(7), C9–C10 136.0(3), C1–P1–C10 90.0(1), C1–P1–P2 93.0(1), C8–P2–P1 95.2(3).

Crystal structure determination of the diphosphorus compound **7** confirmed its molecular constitution. The tricyclic molecule consists of a six-membered, aromatic C_6_ homocycle and two annulated heterocycles, a six-membered P_2_C_4_ and a five-membered PC_4_ ring (Fig. 7). The heterocycles contain localized C=C double bonds with standard bond lengths of 134.3(7) (C7–C8) and 136.0(3) pm (C9–C10). The P–P distance corresponds with 219.59(9) pm to values reported in the literature.^29,30^ The P atoms have trigonal pyramidal surroundings and are stereogenic centers.

The P atoms are disordered over two positions, and all atoms of the molecular centers were refined on split positions (0.72 : 0.28; only the structural parameters of the main component are discussed). The two superimposed molecules represent the enantiomeric diastereomers with the configurations (*R*,*S*) and (*S*,*R*) at the P atoms. The phenyl group attached to P2 is in an *exo*-position (see for comparison the structure of the W complex discussed below). The endocyclic angles C1–P1–C10 and P–P–C are small [90.0(1) and 94.1(av)°], the exocyclic ones are larger (>95°). The C–P–P–C torsion angles of 161.5 (C1–P1–P2–C31) and −147.0° (C10–P1–P2–C8) indicate an approach of the lone pairs at P to an antiperiplanar orientation, which has previously been observed for diphosphines.^29*b*^

Compound **6** shows the expected simple NMR spectra. The ^31^P{^1^H} NMR signal (*δ* = −40.1 ppm) is considerable shifted to a higher field compared to that of the starting phosphine **3a** (*δ* = 68.8 ppm), which may be caused by relaxation of ring strain and the bonding to the relatively electropositive Si atom. Three doublets were observed in the ^29^Si NMR spectrum. A relatively large coupling constant (^1^*J*_PSi_ = 33.8 Hz) was characteristic of the Si atom of the PSiC_4_ heterocycle. Smaller values resulted expectedly for the Si atoms of the SiMe_2_Ph groups (^2^*J*_PSi_ = 26.9 Hz; ^3^*J*_PSi_ = 5.5 Hz). The vinylic H atoms showed ^1^H NMR signals at *δ* = 7.73 ppm (intact PC_4_ ring) and 7.86 ppm (PSiC_4_ ring) with ^3^*J*_PH_ coupling constants of 16.0 (similar to **3b**) and 2.7 Hz. The spectra of **7** were much more complicated. The ^31^P{^1^H} NMR spectrum revealed two doublets of doublets for the diastereomeric (*R*,*S*/*S*,*R*) and (*S*,*S*/*R*,*R*) forms, which showed remarkable differences in their chemical shifts. The (*R*,*S*) form resonated at *δ* = −5.2 ppm (P–Ph) and −16.3 ppm (PC_4_ ring) with a ^1^*J*_PP_ coupling constant of 156.1 Hz, while the (*S*,*S*) form gave two doublets at *δ* = −20.6 ppm (PC_4_ ring) and −77.6 ppm (P–Ph) with a large ^1^*J*_PP_ coupling constant of 354.3 Hz.^29,31^ The molar ratio between both isomers [(*R*,*S*)/(*S*,*R*) to (*R*,*R*)/(*S*,*S*)] in CDCl_3_ depends on temperature and was calculated from the integration ratios of VT ^1^H NMR spectra. It changes from about 1 : 0.41 at room temperature to 1 : 0.26 at 230 K. The ^1^H and ^31^P NMR resonances became broad upon warming to 330 K indicating a fast equilibration at elevated temperature. DFT calculations for both diastereomeric molecules [PBE0 def2-TZVP D3BJ CPCM(CHCl_3_]^26^ reproduced the differences in chemical shifts and coupling constants [(*S*,*R*): *δ* = −27.1 ppm (P1) and −12.9 ppm (P2), ^1^*J*_PP_ = 113.0 Hz; (*S*,*S*): *δ* = −30.5 and −99.8, 323.7 Hz]. The optimized geometries revealed that in both diastereomers the lone pair of electrons at the P atom of the PC_4_ heterocycle is oriented almost perpendicularly to the plane of the sp^2^-hybridized C atoms, which may result in deshielding by anisotropy of the π-orbitals and the ring current of the aromatic ring and in similar chemical shifts for both isomers. The lone pair at the P atom attached to the Ph group of the (*S*,*R*) isomer is almost in an antiperiplanar arrangement with respect to the lone pair of the P atom of the PC_4_ ring (∼180°). This arrangement results in similar ^31^P NMR shifts. For the (*S*,*S*) isomer the lone pairs at the P atoms approach a gauche conformation (torsion angle ∼30°) with the lone pair at the phenyl substituted P atom almost in the nodal plane of the vinylic π-orbital. The approach to the gauche orientation of the lone pairs may not only influence the chemical shifts, but may also result in an additional through-space coupling and may help to understand the increased ^1^*J*_PP_ coupling constant. For the gas phase a relatively small energetic difference was calculated between both diastereomers with a slight preference of the (*RR*,*SS*) over the (*RS*,*SR*) form by 9.3 kJ mol^−1^. The calculated activation barriers for inversion of configuration *via* planar transition states differ with 112.5 kJ mol^−1^ at P2 (P–Ph) and 70.4 kJ mol^−1^ at the P atom of the PC_4_ heterocycle. The values are related on the (*SR*,*RS*) form and may reflect the better delocalisation of the lone pair at P into the PC_4_ ring. Systematic conformational studies on the influence of the relative orientation of the lone pairs at the P atoms of diphosphines on the ^1^*J*_PP_ coupling constants afforded similar results.^29*b*^ A comparable effect has been reported for the coupling between fluorine atoms.^32^

### Coordination compounds of **6** and **7**

**Scheme 5:**
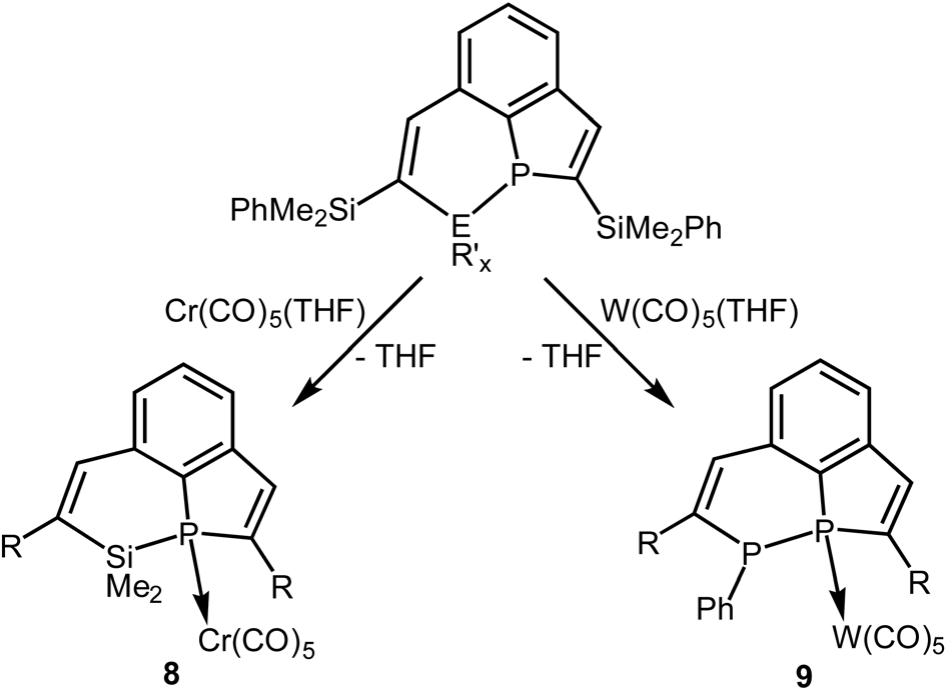
Syntheses of the complexes **8** and **9** (R = SiMe_2_Ph).

Compound **6** was only isolated as a highly viscous liquid which could not be purified by crystallization. Coordination to a transition metal atom should help to generate a crystalline material for purification and further characterization. We treated **6** with an *in situ* generated solution of [Cr(CO)_5_(thf)] in THF at room temperature (Scheme 5). Removal of the solvent, extraction of the residue with *n*-hexane, filtration and concentration of the filtrate afforded yellow crystals of complex **8** in 82% yield. The results of elemental analysis and mass spectrometry were in accordance with the constitution given in Scheme 5.

The resonance of the P atom (*δ* = −12.2 ppm) of **8** is shifted to a higher field compared to that of the starting phosphine **6**, and the ^3^*J*_PH_ coupling constant to the vinylic H atom at the PC_4_ ring increased to 29.3 Hz, which is in accordance with the higher coordination number of four at P. The ^4^*J*_PH_ coupling constant to the H atom attached to the PSiC_4_ heterocycle is, in contrast, with 2.1 Hz almost unchanged compared to that one of **6**. The chiral surrounding of the P atom results in a diastereotopic splitting of the resonances of all three pairs of Me groups. Three doublets were observed in the ^29^Si NMR spectrum, which are shifted to a lower field compared to the resonances in uncoordinated **6** (*δ* = 5.2 *versus* 2.1 ppm for the SiMe_2_ groups in the heterocycle). The NMR parameters of both compounds are very similar and confirm the identity of their constitution. **8** crystallized in very thin platelets. The obtained X-ray data were of insufficient quality and did not allow the refinement to reasonable *R* values and displacement parameters. But they allowed the unambiguous identification of the molecular constitution as depicted in Scheme 5. The analogous W complex was not obtained in a pure form.

In a similar reaction the tricyclic diphosphine **7** was treated with an *in situ* generated solution on [W(CO)_5_(thf)] in THF at room temperature. All volatiles were removed in vacuum, and the crude product was purified by column chromatography (SiO_2_; *n*-hexane/diethyl ether 8 : 1). Recrystallisation from Et_2_O/*n*-hexane afforded colorless crystals of the W complex **9** (Scheme 5) in a moderate yield of 55%. Only two doublets were observed in the ^31^P NMR spectrum. Resonances of a second diastereomer were not found. The relatively large ^1^*J*_PP_ coupling constant (313.8 Hz) corresponds to the value observed for the uncoordinated (*R*,*R*) diastereomer of **7**.

Also the chemical shifts of the P atoms of **9** are closer to those of the (*R*,*R*) form of **7**. They have the characteristically large difference with *δ* = −11.9 ppm for the bridgehead P atom P1 and *δ* = −65.7 ppm for the atom P2 attached to the phenyl group. The coordination to the W(CO)_5_ fragment is confirmed by a large ^1^*J*_PW_ coupling constant to the atom P1 of 211.3 Hz. P2 does not show a coupling to the W atom. In accordance with the increased coordination number at P, the ^3^*J*_PH_ coupling constant to the vinylic H atom at the PC_4_ ring is relatively large with 35.0 Hz.

**Fig. 8:**
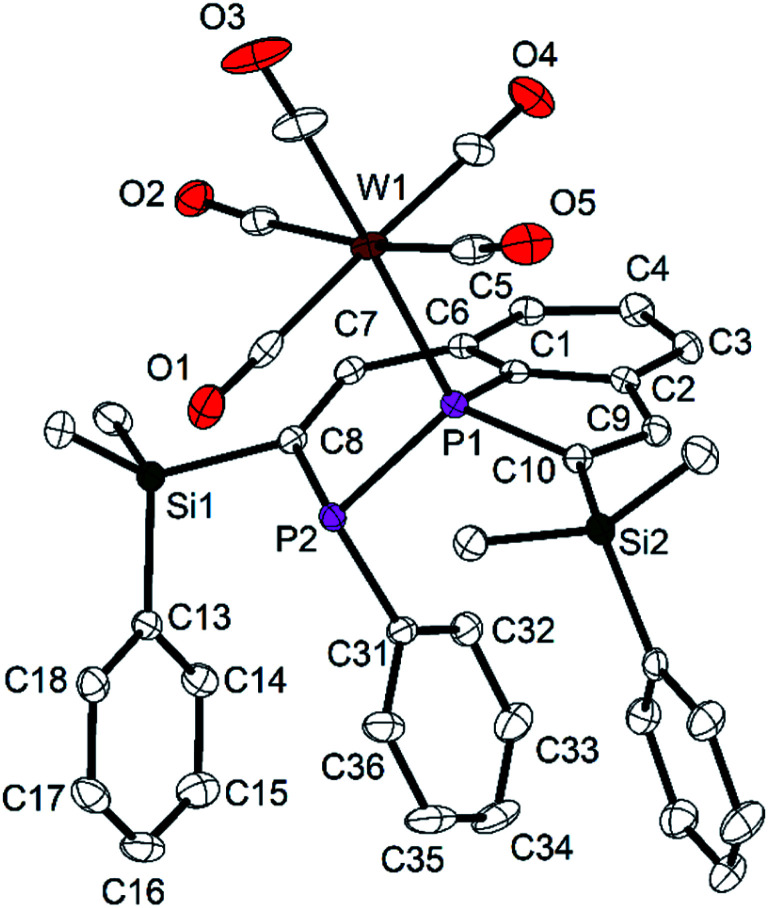
Molecular structure and numbering scheme of **9**. Displacement ellipsoids are drawn at the 40% level. H atoms are omitted; *T* = 100 K. Important bond lengths (pm) and angles (°): P1–P2 219.93(9), W1–P1 252.94(7), P1–C1 177.9(2), P1–C10 182.3(2), P2–C8 185.2(2), P2–C31 182.5(3), C7–C8 134.8(3), C9–C10 135.0(3), C1–P1–C10 92.1(1), C1–P1–P2 100.64(8), C8–P2–P1 95.61(8).

The molecular structure of **9** shows the intact diphosphine coordinated to W(CO)_5_*via* a W–P bond to the bridgehead P atom P1, as predicted by NMR spectroscopy (Fig. 8). The P1–W1 distance [252.9(1) pm] is in the typical range.^33^ The P1–C1 distance [177.9(2) pm] is shorter than that one in **7** [179.8(3) pm], the other P–C distances approach the standard value and are between 182.3(2) and 185.2(2) pm. In contrast to the molecular structure of **7**, the phenyl group attached to P2 adopts an *endo*-position, and the SiMe_2_Ph groups change their orientation with all three phenyl groups in neighboring positions (compare Fig. 2 and 3). Short intramolecular distances between atoms of the aryl groups indicate weak interactions (C36–C27 355.1, C36–H27 316, C36–C28 351.8, C36–H28 309, C36 to C13–C18 376.1 to 395.1 pm). The observed diastereomer has the configuration (*S*,*R*), with its enantiomer generated by the crystallographic center of symmetry of space group *P*2_1_/*c*. This configuration verifies, that complex **9** contains surprisingly the (*R*,*R*) diastereomer of **7** which was detected in solution by NMR spectroscopy as the minor component. This interpretation is in accordance with the NMR spectroscopic findings. Obviously, the coordination to the metal atom favors the shift of the dynamic equilibrium in solution and the rearrangement of the diastereomers of the ligand from (*R*,*S*/*S*,*R*) to (*R*,*R*/*S*,*S*). Rearrangement may be favored by the minimization of steric repulsion between the substituents of the phosphine ligand and the W(CO)_5_ group. This assumption is supported by the determination of the “percent buried volumes” % *V*_bur_,^34^ which were calculated based on the optimized geometries of the diasteromers of **7**. P1 was experimentally observed as the binding partner of the metal atom, and we obtained values of 40.4 (*S*,*R*) and 32.8% (*S*,*S*) which confirmed the smaller steric demand of the (*S*,*S*/*R*,*R*) couple and the resulting smaller steric stress in complex **9**.

### Reaction of **3a** with H_2_O_2_ – oxidative ring cleavage

**Scheme 6:**
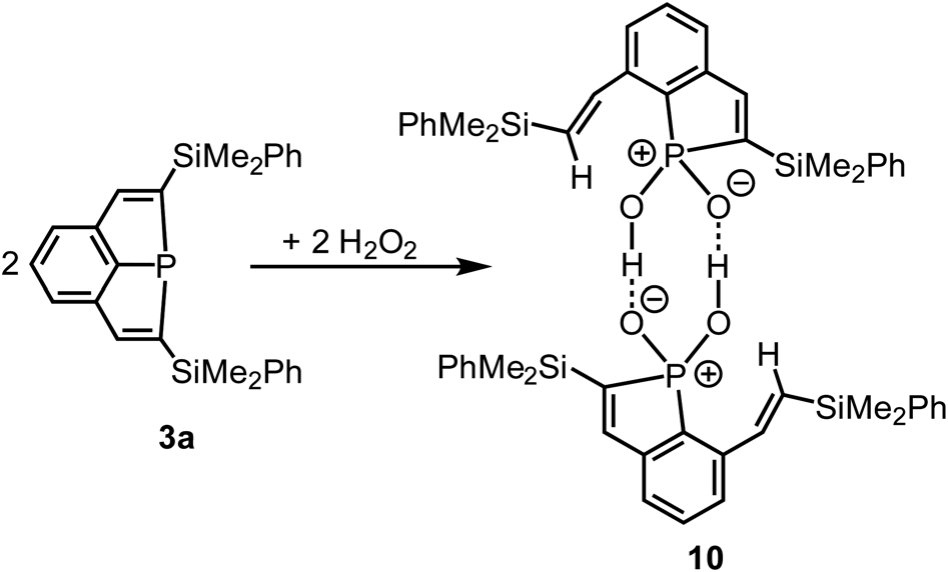
Reaction of **3b** with H_2_O_2_.

Ag^+^ and a strained phosphine of type **2** (Scheme 1) afforded by oxidation a remarkable radical cation.^14^ As shown by preliminary investigations, **3a** did, in contrast, not react with Ag^+^ by oxidation, but by formation of a simple adduct with the Ag cation coordinated to the P atom. This behaviour resembles that one of Au^+^.^1,7^ The oxidant H_2_O_2_ reacts with tertiary phosphines by oxidation of the P atoms, formation of phosphine oxides and release of water.^35,36^ Recently the highly selective synthesis of Ph_3_PO in a 100% yield was achieved on such a route.^36^ The highly strained ring system of compounds **3** may result in another reaction course, and we, therefore, treated a cooled (−78 °C) solution of **3a** in *n*-pentane with a concentrated H_2_O_2_ solution in water (Scheme 6). The mixture was slowly warmed to room temperature, all volatiles were removed in vacuum, and the residue was recrystallized from a mixture of CH_2_Cl_2_ and Et_2_O. Compound **10** was isolated as colorless crystals in 79% yield.

**Fig. 9:**
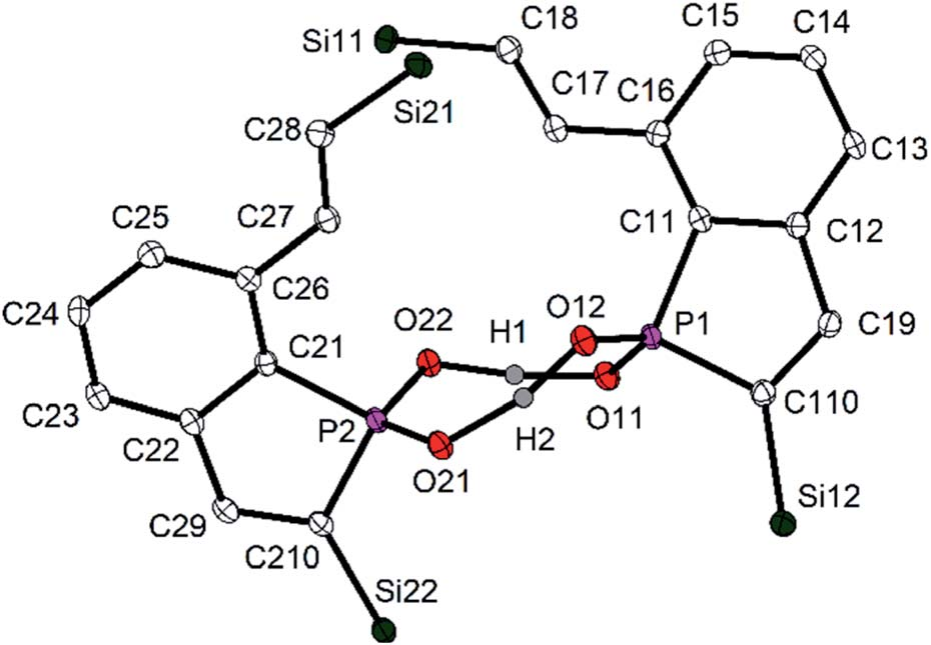
Molecular structure and numbering scheme of **10**. Displacement ellipsoids are drawn at the 40% level; *T* = 100 K. H atoms with exception of H1 and H2 and the SiMe_2_Ph groups except the central Si atoms are omitted. Important bond lengths (pm) and angles (°): P1–O11 153.7(2), P1–O12 151.6(2), P2–O21 153.4(2), P2–O22 151.8(2), P–C 179.6(av), O11–P1–O12 113.00(9), O21–P2–O22 112.85(9).

Crystal structure determination (Fig. 9) confirmed cleavage of a P–C bond of one of the PC_4_ rings of **3a**, the oxidation of the P atom and the formation of a phosphinic acid, R_2_PO_2_H, by formal insertion of the P atom into the O–O bond of the peroxide. Ring cleavage and H transfer resulted in an alkenyl side-chain with the *trans*-arrangement of two H atoms at the C=C bond. Hence, the reaction of **3a** with H_2_O_2_ is completely different from those usually observed with tertiary phosphines. **10** is dimeric in the solid state with a central, non-planar P_2_O_4_H_2_ heterocycle. The O–H distances (∼125 pm) are in a narrow range and indicate together with the short O–O distances of 250.0 pm on average two symmetric O–H–O hydrogen bonds.^37^ The P–O bond lengths [151.6(2) to 153.7(2) pm] are in the typical range of phosphinic acids, for which hydrogen bonding results in various structural motifs with dimeric, oligomeric and polymeric species in the solid state.^38^ The dimer of **10** is not centrosymmetric, both molecular halfes are instead related by a *pseudo*-twofold rotational axis perpendicular to the P_2_O_4_H_4_ ring. The P atom of **10** resonated at *δ* = 62.8 ppm in the ^31^P{^1^H) NMR spectrum in C_6_D_6_. The vinylic H atom at the PC_4_ ring showed a doublet at *δ*(^1^H) = 6.93 ppm with a large ^3^*J*_PH_ coupling constant of 51.6 Hz indicating the increased coordination number and oxidation state of P. The protons of the vinylic side-chain were detected at *δ* = 8.15 ppm (α to the central aryl ring) and 6.75 (H–C–Si), the ^3^*J*_HH_ coupling constant of 18.9 Hz confirmed their *trans*-arrangement. The vinylic C atoms showed the usual ^13^C NMR shifts between *δ* = 133.6 and 153.0 ppm. The proton of the P–OH group was detected as a broad singlet. Its chemical shift strongly depended on concentration and varied between 10.00 and 12.60 ppm, which may correlate with an increasing dissociation of the dimeric formula units into monomeric fragments in diluted solution. Mass spectrometry confirmed the constitution of **10** by the characteristic molecular ion peak at *m*/*z* = 459 for the monomeric formula unit.

## Conclusions

The sterically constrained tricyclic phosphine **3a** with annulated five- and six-membered rings and the P atom homoleptically bound to C atoms shows fascinating chemical and redox reactivity in various transformations, which resulted in coordination to metal atoms, oxidative addition to the P atoms and the generation of dimethyl phosphoranes, insertion into one of the rings by ring expansion *etc.* We reported here on reductive or oxidative ring cleavage. Applying cyclic voltammetry, square-wave voltammetry combined with mechanistic studies by the use of *in situ* UV-vis CV and multi-pulse chronoamperometry allowed detailed investigation of the reduction reaction. Reduction of phosphine **3a** leads to dianion **[3a′]2−***via* an EEC reaction mechanism consisting of a two-electron transfer followed by a fast ring-opening reaction to dianion **[3a′]2−**. The electrochemical reduction is a reversible reaction proceeding *via* a metastable intermediate **3a′** under ring closure back to phosphine **3a**. Electrochemical investigations predicted the formation of a stable dianion **[3a′]2−** by a two-electron reduction at *E*_1/2_ = −2.58 V (*vs. E*_1/2_(Cp_2_Fe/Cp_2_Fe^+^)). Inspired by these results we treated phosphine **3a** with Li and isolated the unique dilithium compound **5** by cleavage of one of the PC_4_ heterocycles. **5** represents a phosphaindole derivative with annulated aromatic C_6_ and PC_4_ rings and has a promising perspective for an application in secondary processes. Preliminary reactions with SiMe_2_Cl_2_ or PhPCl_2_ afforded by salt elimination new ring systems, in which Si or P atoms were incorporated into six-membered heterocycles and which formally represent products of a ring expansion of **3a**. The resulting diphosphine (**7**) afforded a mixture of diastereomers, which showed a dynamic equilibrium in solution. The (*R*,*S*) form was detected by crystal structure determination. Configuration change was observed upon coordination to W(CO)_5_. Minimization of steric repulsion seem to favour rearrangement to yield exclusively the (*R*,*R*) form of **7** coordinated to the metal atom. Hydrogen peroxide reacts usually with triorganylphosphines by transfer of one O atom and formation of triorganylphosphine oxides. In contrast, the strained ring system of **3a** led to cleavage of a P–C bond and formation of a phosphinic acid derivative by formal insertion of the P atom into the O–O bond of the peroxo group. Once again these reactions underscore impressively the fascinating capability of these tricyclic phosphines to be applied in various chemical transformations and in the generation of unprecedented structural motifs.

## Experimental section

### General considerations

All manipulations were carried out under purified argon, using standard Schlenk techniques. Solvents were distilled from drying agents and degassed (THF, diethyl ether and toluene over sodium/benzophenone; *n*-pentane and *n*-hexane over LiAlH_4_). NMR spectra were recorded in C_6_D_6_, d^8^-THF and CDCl_3_ using the following Bruker instruments: Avance I (^1^H, 400.13; ^13^C, 100.62; ^31^P, 161.98), Avance III (^1^H, 400.03; ^13^C, 100.60; ^31^P, 161.93 MHz) and referenced internally to residual solvent resonances (chemical shift data in *δ*). ^13^C and ^31^P NMR spectra were usually proton-decoupled. The assignment of NMR spectra is based on HSQC, HMBC, DEPT135, DEPT19.5 and H,H-ROESY data. Elemental analyses were determined by the microanalytic laboratory of the Westfälische Wilhelms Universität Münster. IR spectra were recorded as KBr pellets on a Shimadzu Prestige 21 spectrometer, electron impact mass spectra on a Finnigan MAT 95 mass spectrometer, HRMS (ESI-TOF) on Orbitrap LTQXL. Commercially available Me_2_SiCl_2_, PhPCl_2_, [Cr(CO)_6_] and [W(CO)_6_] were used as purchased. The starting tricyclic phosphine **3a**,^7^ [Cr(CO)_5_(thf)] and [W(CO)_5_(thf)]^39^ were synthesized according to literature procedures.

### Cyclic voltammetry (CV)

All electrochemical experiments were performed in a nitrogen filled glovebox with specially dried (see SI) solvents and [^*n*^Bu_4_N][OTf] as a supporting electrolyte by a PGSAT302 (Metrohm Autolab, Netherlands). CV measurements were performed in custom made 10 mL cells with platinum disk working electrodes (*d* = 1.6 mm diameter, ALS Co. Ltd., Japan) and a platinum wire coil (200 × 0.5 mm) as a counter electrode. Working electrodes were wet polished (1 μm polishing diamond, 0.05 μm polishing alumina) on different polishing pads before use. Reference electrodes of first-order, consisting of a silver wire in 0.01 M AgNO_3_ solution in acetonitrile with 0.1 M [^*n*^Bu_4_N][OTf] as supporting electrolyte are used. Working and counter electrodes were employed without any separation, while a Vycor glass cylinder (*D* = 3 mm, *L* = 4 mm, ALS Co. Ltd., Japan) was used to separate the reference electrolyte from the substrate solution. Electrochemical measurements with disk electrodes in analytical cells are referenced by adding an equimolar amount of Cp_2_Fe, calibrating the *E*_1/2_(Cp_2_Fe/Cp_2_Fe^+^) by cyclic voltammetry as a zero point according to the IUPAC procedure.^40^ Peak potentials and half wave potentials of the substrates where measured internally in case no reaction with ferrocene takes place. Spectroelectrochemical measurements in cuvette-cell were externally referenced by calibration of the reference electrode in an analytical cell or by calibrating the reversible half-wave potentials to measurements at disk electrodes.

### *In situ* UV-vis spectroelectrochemistry (SEC) in transmission geometry

*In situ* UV-vis SEC was performed with a Maya 2000PRO (Ocean Optics, Ostfildern, Germany) diode array spectrometer equipped with a 25 μm slit, grating JC-1 (300 lines per mm), resulting wavelength resolution of 1.1 nm and a DH-2000-BAL (Ocean Optics Ostfildern, Germany) balanced and adjustable deuterium-halogen light source. Transmission measurements were performed in a 0.5 mm pathway fused silica cuvette (ALS, Japan) equipped with a platinum gauze (80 mesh, 6 × 7 mm) electrode as working electrode and counter electrode. Working and counter electrode meshes were spot-welded to a Pt wire (0.08 mm) and embedded into perforated office lamination foil by heat treatment in special developed procedures. Mesh electrodes were conditioned by 30 min galvanostatic cycling between ±500 mA for 30 s in 1.0 M nitric acid. A standard cell holder installed in the glovebox with adjustable collimation lenses attached to 600 μm solarization resistant optical fibers were used to focus the light beam onto the working electrode. All *in situ* spectroelectrochemical cells were equipped with the same reference electrode used for disk electrode measurements. UV-vis SEC spectra in *in situ* measurements were recorded as relative absorbance data with the start material as a reference. All spectral data were recorded after optimization of the halogen light intensity relative to the deuterium lamp. Minimized integration times (*t* = 34–50 ms) are used with an averaging of 15 scans for each spectrum.

### Dilithium phosphide **5**

Phosphine **3a** (0.30 g, 0.70 mmol), lithium (0.010 g, 1.44 mol) and naphthalene (0.18 g, 1.40 mmol) were cooled to −78 °C and suspended in 15 mL of THF. The mixture was slowly warmed to room temperature overnight. Filtration and removal of all volatiles in *vacuo* afforded a solid material, which was carefully evacuated to about 5 × 10^−2^ mbar and dissolved in a small quantity of toluene at 70 °C. The solution was slowly cooled to 3 °C to afford **5** as yellow blocks (0.25 g, 70%). Mp: 175 °C (dec.). The assignment of NMR resonances is based on the numbering scheme given in Scheme 3. ^1^H NMR (d_8_-THF, 300 K, 400 MHz): *δ* 9.02 (s, 1H, 7-*H*), 7.57 (overlap, m, 4H, *o-H* 8- and 10-Si-Ph), 7.50 (d, ^3^*J*_HH_ = 7.7 Hz, 1H, 3-*H*), 7.47 (d, ^3^*J*_PH_ = 11.3 Hz, 1H, 9-*H*), 7.23 (m, 2H, *m-H* 8-Si-Ph), 7.17 (m, 1H, *p-H* 8-Si-Ph), 7.16 (overlap, m, 3H, *m*- and *p-H* 10-Si-Ph), 6.85 (*pseudo*-t br., ^3^*J*_HH_ = 7.3 Hz, 1H, 4-*H*), 6.75 (d, ^3^*J*_HH_ = 6.7 Hz, 1H, 5-*H*), 0.54 (s, 6H, 10-Si-C*H*_3_), 0.36 (s, 6H, 8-Si-C*H*_3_). ^13^C{^1^H} NMR (d_8_-THF, 300 K, 101 MHz): *δ* 198.5 (d, ^2^*J*_PC_ = 34.5 Hz, 8-*C*), 164.4 (d, ^*3*^*J*_PC_ = 5.5 Hz, 7-*C*), 150.1 (d, ^1^*J*_PC_ = 34.6 Hz, 1-*C*), 145.9 (s, *i-C* 8-Si-Ph), 145.1 (overlap, d, ^1^*J*_PC_ = 54.0 Hz, 10-*C*), 144.8 (overlap, s, 2-*C*), 144.3 (s, *i*-C 10-Si-Ph), 142.7 (d, ^2^*J*_PC_ = 3.2 Hz, 6-*C*), 135.0 (s, *o-C* 10-Si-Ph), 134.9 (s, *o-C* 8-Si-Ph), 128.4 (s, *p-C* 8- and 10-Si-Ph), 128.1 (s br., 9-*C*), 127.7 (s, *m-C* 8-Si-Ph), 127.6 (s, *m-C* 10-Si-Ph), 124.3 (d, ^3^*J*_PC_ = 8.5 Hz, 5-*C*), 122.1 (s, 3-*C*), 118.4 (s, 4-*C*), 0.5 (d, ^3^*J*_PC_ = 4.2 Hz, 10-Si-*C*H_3_), −0.2 (s, 8-Si-*C*H_3_). ^29^Si{^1^H} NMR (d_8_-THF, 300 K, 79 MHz): *δ* −12.0 (d, ^2^*J*_PSi_ = 34.7 Hz, 10-*Si*), −14.1 (d, ^3^*J*_PSi_ = 9.2 Hz, 8-*Si*). ^31^P{^1^H} NMR (d_8_-THF, 300 K, 162 MHz): *δ* = 45.1. ^7^Li{/} NMR (d_8_-THF, 300 K, 156 MHz): *δ* = 1.00 (br.). IR (KBr, cm^−1^): 3063 w, 3044 m, 3019 w, 2999 w, 2951 s, 2889 m, 2837 vw *ν*(CH); 1954 vw, 1892 vw, 1817 vw, 1651 vw, 1591 w, 1562 w, 1541 vw, 1528 vw, 1489 w, 1454 w, 1423 s, 1406 w *ν*(C=C), phenyl; 1356 vw, 1304 w, 1285 w, 1246 vs *δ*(CH_3_); 1175 w, br., 1113 s, 1065 vw, 1038 m, 986 m, 968 m, 955 m, 916 vw, 887 vw, 833 vs, 812 vs, 773 s, 729 vs *ν*(CC), *ν*(CO), *ρ*(SiCH_3_); 698 vs, 652 w, 613 w *ν*(SiC), phenyl; 590 vw, 509 m, 469 m *ν*(PC), *δ*(CC). Anal. calcd for C_30_H_35_Li_2_OPSi_2_ (512.6 for the monomer): C, 70.3, H, 6.9. Found: C, 70.5; H, 6.9.

### Synthesis of **6** (SiMe_2_)

**5** (0.15 g, 0.29 mmol of the monomer) was dissolved in 5 mL of THF, and Cl_2_SiMe_2_ (39 μL, 0.041 g, 0.32 mmol; density: 1.06 g mL^−1^) was added at room temperature. The yellow color of **5** disappeared immediately, and the colorless solution was stirred for 1 h. All volatiles were removed in *vacuo*, and the residue was treated with 10 mL of *n*-pentane. The suspension was filtered, and the solvent of the filtrate removed in *vacuo*. **6** was obtained as a colourless oil, which did not crystallize from various solvents (0.12 g, 85%). The assignment of NMR resonances is based on the numbering scheme given in Scheme 3. ^1^H NMR (C_6_D_6_, 300 K, 400 MHz): *δ* 7.86 (d, ^4^*J*_PH_ = 2.7 Hz, 1H, 7-*H*), 7.73 (d, ^3^*J*_PH_ = 16.0 Hz, 1H, 9-*H*), 7.54 (m, 2H, *o-H* 10-Si-Ph), 7.50 (m, 2H, *o-H* 8-Si-Ph), 7.48 (d, ^3^*J*_HH_ = 7.4 Hz, 1H, 3*-H*), 7.19 (t, ^3^*J*_HH_ = 7.6 Hz, 1H, 4-*H*), 7.16 (overlap, m, 6H, *o*- and *m-H* 8- and 10-Si-Ph), 6.91 (dd, ^3^*J*_HH_ = 7.3 Hz, ^4^*J*_PH_ = 3.6 Hz, 1H, 5-*H*), 0.53 (s, 6H, 10-SiC*H*_3_Ph), 0.40 (s, 6H, 8-Si-C*H*_3_Ph), −0.21 (d, ^3^*J*_PH_ = 5.0 Hz, 6H, ring Si*Me*_2_). ^13^C{^1^H} NMR (d_8_-THF, 300 K, 101 MHz): *δ* 156.6 (d, ^3^*J*_PC_ = 1.4 Hz, 7-*C*), 147.1 (d, ^1^*J*_PC_ = 3.6 Hz, 1-*C*), 147.0 (d, ^2^*J*_PC_ = 12.0 Hz, 2-*C*), 145.8 (d, ^1^*J*_PC_ = 48.4 Hz, 10-*C*), 145.5 (s, 9-*C*), 139.0 (s, *i-C* 8-Si-Ph), 138.8 (d, ^3^*J*_PC_ = 1.6 Hz, *i-C* 10-Si-Ph), 138.0 (d, ^2^*J*_PC_ = 6.9 Hz, 6-*C*), 136.2 (d, ^2^*J*_PC_ = 12.7 Hz, 8-*C*), 134.5 (s, *o-C* 8-Si-Ph), 134.4 (s, *o-C* 10-Si-Ph), 129.5 (s, *p-C* 10-Si-Ph), 129.4 (s, *p-C* 8-Si-Ph), 128.2 (overlap, s, *m-C* 8- and 10-Si-Ph), 126.1 (s, 4-*C*), 124.1 (s, 3-*C*), 123.5 (d, ^3^*J*_PC_ = 4.2 Hz, 5-*C*), −1.1 (d, ^3^*J*_PC_ = 3.4 Hz, 10-Si-*C*H_3_), −1.3 (s, 8-Si-*C*H_3_), −2.5 (d, ^2^*J*_PC_ = 11.1 Hz, ring SiMe_2_). ^29^Si{^1^H} NMR (d_8_-THF, 300 K, 79 MHz): *δ* 2.1 (d, ^1^*J*_PSi_ = 33.8 Hz, ring *Si*Me_2_), −5.4 (d, ^3^*J*_PSi_ = 5.5 Hz, 8-*Si*), −10.9 (d, ^2^*J*_PSi_ = 26.9 Hz, 10-*Si*). ^31^P{^1^H} NMR (d_8_-THF, 300 K, 162 MHz): *δ* −40.1 (s). IR (KBr, cm^−1^): 3067 m, 3049 m, 3011 w, 2955 s *ν*(CH); 1954 vw, 1819 vw, 1775 vw, 1722 vw, 1688 vw, 1653 vw, 1524 s *ν*(C=C), phenyl; 1425 m, 1406 w, 1306 w, 1248 vs *δ*(CH); 1190 vw, 1159 vw, 1111 s, 1040 m, 959 s, 907 s, 833 vs, 812 vs, 790 vs, 777 vs, 731 vs *ν*(CC), *ρ*(SiCH_3_); 700 vs, 654 m, 598 vw *ν*(SiC), phenyl; 484 w, 471 m, 453 w, 422 w *ν*(PC), *δ*(CC). MS (EI, 20 eV, 303 K): *m*/*z* (%) = 484 (100) [M]^+^, 135 (10) [SiMe_2_Ph]^+^. HRMS (ESI-ORBITRAP) *m*/*z*: [M + H]^+^ calcd for C_28_H_34_Si_3_P 485.1700; found 485.1709; [M + OH]^+^ C_28_H_34_OSi_3_P 501.1650; found 501.1651.

### Synthesis of **7** (PPh)

A solution of compound **5** in 10 mL of THF was prepared from **3a** (0.15 g, 0.35 mmol), Li (4.9 mg, 0.71 mmol) and naphthalene (0.090 g, 0.70 mmol) as described above. It was cooled to −78 °C and directly treated with PhPCl_2_ (57 μL, 0.075 g, 0.42 mmol; density: 1.32 g mL^−1^). The cooling bath was removed and the mixture stirred at room temperature for 3 h. All volatiles were removed *in vacuo*, and the residue was extracted with 10 mL of *n*-hexane. After filtration, concentration of the filtrate and addition of a seed crystal **7** crystallised at room temperature as yellow blocks (0.16 g, 85%). Alternatively, the solvent of the filtrate was completely removed *in vacuo* and the residue treated with refluxing methanol (7 mL), until a clear solution was formed. Cooling the solution to room temperature led to the separation of pure **7** as a yellow oil, which was isolated and recrystallized from *n*-hexane. Mp: 89 °C. The assignment of NMR resonances is based on the numbering scheme given in Scheme 3. *NMR data of the R,S/S,R diastereomer:* signals of the *para*-C- and -H atoms of the silicon bound phenyl rings overlapped and were not assigned. ^1^H NMR (CDCl_3_, 300 K, 400 MHz): *δ* 7.66 (overlap, m, 2H, *o-H* P-Ph), 7.63 (overlap, m, 1H, 9-*H*), 7.44 (overlap, m, 1H, 3-*H*), 7.34 (overlap, m, 1H, *p-H* P-Ph), 7.31 (overlap, m, 2H, *o-H* 10-Si-Ph), 7.29 (overlap, m, 1H, 4-*H*), 7.28 (overlap, m, 2H, *o-H* 8-Si-Ph), 7.26 (overlap, m, 4H, *m-H* 8- and 10-Si-Ph), 7.24 (d, ^3^*J*_PH_ = 14.0 Hz, 7-*H*), 7.17 (t, ^3^*J*_HH_ = 7.5 Hz, 2H, *m-H* P-Ph), 6.83 (dd, ^3^*J*_HH_ = 7.3 Hz, ^4^*J*_PH_ = 3.1 Hz, 5-*H*), 0.19 (overlap, s, 6H, 8- and −10-Si-C*H*_3_^1^), 0.14 (s, 3H, 10-Si-C*H*_3_^2^), 0.13 (s, 3H, 8-Si-C*H*_3_^2^). ^13^C{^1^H} NMR (CDCl_3_, 300 K, 101 MHz): *δ* 151.0 (dd br., ^1^*J*_PC_ = 55.0 Hz, ^2^*J*_PC_ = 8.9 Hz, 10-*C*), 149.5 (s br., 9-*C*), 147.0 (dd br., ^3^*J*_PC_ = 9.0 Hz, ^2^*J*_PC_ = 2.0 Hz, 7-*C*), 146.2 (dd br., ^2^*J*_PC_ = 12.3 Hz, ^3^*J*_PC_ = 2.0 Hz, 2-*C*), 144.5 (d br., ^1^*J*_PC_ = 9.1 Hz, 1-*C*), 138.8 (dd, ^1^*J*_PC_ = 50.8 Hz, ^2^*J*_PC_ = 5.2 Hz, 8-*C*), 137.3 (dd, ^2^*J*_PC_ = 9.1 Hz, ^3^*J*_PC_ = 5.3 Hz, 6-*C*), 137.0 (dd, ^*n*^*J*_PC_ = 22.2 Hz, ^*n*^*J*_PC_ = 10.7 Hz, *o-C* P-Ph), 137.0 (s, *i-C* 8- and 10-Si-Ph), 134.0 (s, *o-C* 8-Si-Ph), 133.9 (s, *o-C* 10-Si-Ph), 133.8 (overlap, m, *i-C* P-Ph), 129.9 (s, *p-C* P-Ph), 128.8 (d, ^3^*J*_PC_ = 8.3 Hz, *m-C* P-Ph), 128.4 (s, 4-*C*), 127.7 (overlap, *m-C* 8- and 10-Si-Ph), 123.7 (s, 3-*C*), 121.7 (d, ^3^*J*_PC_ = 4.8 Hz, 5-*C*), −1.1 (s, 8-Si-*C*H_3_^2^), −1.2 (s, 8-Si-*C*H_3_^1^), −1.7 (d, ^3^*J*_PC_ = 2.0 Hz, 10-Si-*C*H_3_^2^), −2.1 (d, ^3^*J*_PC_ = 2.8 Hz, 10-Si-*C*H_3_^1^). ^29^Si{^1^H} NMR (CDCl_3_, 300 K, 79 MHz): *δ* −4.5 (dd, ^3^*J*_PSi_ = 8.5 Hz, ^2^*J*_PSi_ = 4.1 Hz, 8-*Si*), −10.7 (d, ^2^*J*_PSi_ = 25.3 Hz, 10-*Si*). ^31^P{^1^H} NMR (CDCl_3_, 300 K, 162 MHz): *δ* −5.2 (d, ^1^*J*_PP_ = 156.1 Hz, *P*-Ph), −16.3 (d, ^1^*J*_PP_ = 156.1 Hz, *P*C_4_ heterocycle). *NMR data of the R,R/S,S diastereomer:* signals of the *ortho-, meta-* and *para*-C and -H atoms of the silicon bound phenyl rings overlapped and were not assigned. ^1^H NMR (CDCl_3_, 300 K, 400 MHz): *δ* 7.83 (d, ^3^*J*_PH_ = 16.0 Hz, 1H, 7-*H*), 7.39 (overlap, m, 1H, 9-*H*), 7.31 (overlap, m, 1H, 3-*H*), 7.27 (overlap, m, 1H, 4-*H*), 7.06 (dd, ^3^*J*_HH_ = 7.4 Hz, ^4^*J*_HP_ = 3.0 Hz, 1H, 5-*H*), 6.91 (t, ^3^*J*_HH_ = 6.9 Hz, *p-H* P-Ph), 6.76 (*pseudo*-t, ^3^*J*_HH_ = 7.2 Hz, 2H, *m-H* P-Ph), 6.40 (*pseudo*-t, ^3^*J*_HH_ = 7.1 Hz, ^3^*J*_PH_ = 8.0 Hz, 2H, *o-H* P-Ph), 0.67 (s, 3H, 10-Si-C*H*_3_^1^), 0.64 (s, 3H, 10-Si-C*H*_3_^2^), 0.56 (s, 3H, 8-Si-C*H*_3_^1^), 0.51 (s, 3H, 8-Si-C*H*_3_^2^). ^13^C{^1^H} NMR (CDCl_3_, 300 K, 101 MHz): *δ* 150.3 (dd br., ^3^*J*_PC_ = 11.0 Hz, ^2^*J*_PC_ = 6.0 Hz, 7-*C*), 149.4 (s, 9-*C*), 145.2 (d br., ^1^*J*_PC_ = 62.0 Hz, 10-*C*), 143.5 (d, ^3^*J*_PC_ = 18.2 Hz, ^2^*J*_PC_ = 2.0 Hz, 2-*C*), 143.2 (m br., 1-*C*), 138.2 (s br., *i-C* 10-Si-Ph), 137.2 (overlap, m, *i-C* 8-Si-Ph), 135.5 (dd, ^*n*^*J*_PC_ = 8.5 Hz, ^*n*^*J*_PC_ = 3.7 Hz, 6-*C*), 131.1 (d, ^*n*^*J*_PC_ = 19.8 Hz, *o-C* P-Ph), 130.6 (d br., ^1^*J*_PC_ = 58.0 Hz, 8-*C*), 128.0 (s, *p-C* P-Ph), 127.0 (s, 4-*C*), 127.0 (m, *m-C* P-Ph), 124.2 (s, 3-*C*), 122.5 (d, ^3^*J*_PC_ = 4.6 Hz, 5-*C*), −1.0 (s, 10-Si-*C*H_3_^1^), −1.1 (s, 10-Si-*C*H_3_^2^), −2.6 (d br., ^3^*J*_PC_ = 6.0 Hz, 8-Si-*C*H_3_^1^), −3.0 (d br., ^3^*J*_PC_ = 6.0 Hz, 8-Si-*C*H_3_^2^). ^29^Si{^1^H} NMR (CDCl_3_, 300 K, 79 MHz): *δ* −4.5 (overlap, m, 8-*Si*), −10.7 (overlap, m, 10-*Si*). ^31^P{^1^H} NMR (CDCl_3_, 300 K, 162 MHz): *δ* −20.6 (dd, ^1^*J*_PP_ = 354.3 Hz, *P*C_4_ heterocycle), −77.6 (d, ^1^*J*_PP_ = 354.3 Hz, *P*-Ph). IR (KBr, cm^−1^): 3063 w, 3044 w, 3021 w, 3011 w, 2955 m, 2899 vw *ν*(CH); 1958 vw, 1931 vw, 1896 vw, 1877 vw, 1813 vw, 1769 vw, 1653 vw, 1584 vw, 1564 vw, 1522 w *ν*(C=C), phenyl; 1489 w, 1429 m, 1404 w, 1331 vw, 1296 w, 1246 s *δ*(CH); 1186 vw, 1159 vw, 1111 s, 1042 m, 995 w, 953 s, 883 vs, 833 vs, 806 vs, 779 vs, 735 vs *ν*(CC), *ρ*(SiCH_3_); 698 vs, 656 m, 623 w *ν*(SiC), phenyl; 590 vw, 527 m, 500 w, 484 vw, 461 m, 434 vw *ν*(PC), *δ*(CC). MS (EI, 20 eV, 353 K): *m/z* (%) = 534 (100) [M]^+^, 135 (32) [SiMe_2_Ph]^+^. Anal. calcd for C_32_H_32_P_2_Si_2_ (534.7): C, 71.9, H, 6.0. Found: C, 71.4; H, 6.2.

### Synthesis of the Cr complex **8**

[Cr(CO)_6_] (0.19 g, 0.86 mmol) was dissolved in 10 mL of THF and irradiated by a mercury vapor lamp for 6 h. The orange solution was added to neat **6** (0.34 g, 0.70 mmol) at room temperature, and the mixture was stirred overnight. All volatiles were removed *in vacuo* at 40 °C. The residue was treated with 10 mL of *n*-hexane. The suspension was filtered. The product crystallised from the concentrated filtrate at room temperature (0.39 g, 82%). Mp: 110 °C. The assignment of NMR resonances is based on the numbering scheme given in Scheme 3. ^1^H NMR (CDCl_3_, 300 K, 400 MHz): *δ* 7.69 (d, ^4^*J*_PH_ = 2.1 Hz, 1H, 7-*H*), 7.48 (m, 2H, *o-H* 10-Si-Ph), 7.40 (d, ^3^*J*_PH_ = 29.3 Hz, 1H, 9-*H*), 7.37 (m, 2H, *o-H* 8-Si-Ph), 7.15 (overlap, m, 4H, *m-H* 8- and 10-Si-Ph), 7.14 (overlap, m, 2H, *p-H* 8- and 10-Si-Ph), 7.04 (d, ^3^*J*_HH_ = 7.5 Hz, 1H, 3-*H*), 6.98 (t, ^3^*J*_HH_ = 7.5 Hz, 1H, 4-*H*), 6.68 (dd, ^3^*J*_HH_ = 7.4 Hz, ^4^*J*_PH_ = 4.4 Hz, 1H, 5-*H*), 0.61 (s, 6H, 10-Si-C*H*_3_), 0.50 (s, 3H, 8-Si-C*H*_3_^1^), 0.45 (s, 3H, 8-Si-C*H*_3_^2^), 0.40 (d, ^3^*J*_PH_ = 8.6 Hz, P–Si–C*H*_3_^1^), −0.71 (d, ^3^*J*_PH_ = 4.6 Hz, P–Si–C*H*_3_^2^). ^13^C{^1^H} NMR (CDCl_3_, 300 K, 101 MHz): *δ* 222.0 (d, ^2^*J*_PC_ = 6.6 Hz, Cr–CO axial), 217.3 (d, ^3^*J*_PC_ = 10.0 Hz, Cr–CO equatorial), 155.8 (d, ^3^*J*_PC_ = 2.0 Hz, 7-*C*), 149.8 (d, ^1^*J*_PC_ = 16.8 Hz, 10-*C*), 149.5 (d, ^2^*J*_PC_ = 2.9 Hz, 9-*C*), 147.4 (d, ^1^*J*_PC_ = 24.2 Hz, 1-*C*), 145.5 (d, ^3^*J*_PC_ = 15.8 Hz, 2-*C*), 138.3 (d, ^2^*J*_PC_ = 5.0 Hz, 6-*C*), 138.0 (s, *i*-C 8-Si-Ph), 137.5 (d, ^3^*J*_PC_ = 2.7 Hz, *i-C* 10-Si-Ph), 137.0 (d, ^2^*J*_CP_ = 7.9 Hz, 8-*C*), 134.6 (s, *o-C* 8-Si-Ph), 134.4 (s, *o-C* 10-Si-Ph), 129.8 (s, *p-C* 10-Si-Ph), 129.7 (s, *p-C* 8-Si-Ph), 129.5 (s, 4-*C*), 128.4 (s, *m-C* 10-Si-Ph), 128.3 (s, *m-C* 8-Si-Ph), 125.2 (d, ^3^*J*_PC_ = 5.8 Hz, 5-*C*), 124.9 (d, ^3^*J*_PC_ = 4.6 Hz, 3-*C*), −0.9 (s, 10-Si-*C*H_3_^1^), −1.2 (s, 8-Si-*C*H_3_^2^), −1.5 (d, ^3^*J*_PC_ = 2.6 Hz, 10-Si-*C*H_3_^2^), −1.7 (s, 8-Si-*C*H_3_^1^), −2.5 (d, ^2^*J*_PC_ = 15.3 Hz, P–Si–*C*H_3_^1^), −4.3 (d, ^2^*J*_PC_ = 1.1 Hz, P–Si–*C*H_3_^2^). ^29^Si{^1^H} NMR (CDCl_3_, 300 K, 79 MHz): *δ* 5.2 (d, ^1^*J*_PSi_ = 13.5 Hz, P–*Si*), −4.4 (d, ^3^*J*_PSi_ = 7.3 Hz, 8-*Si*), −10.7 (d, ^2^*J*_PSi_ = 22.3 Hz, 10-*Si*). ^31^P{^1^H} NMR (CDCl_3_, 300 K, 162 MHz): *δ* −12.2 (s). IR (KBr, cm^−1^): 3048 vw, 2961 w *ν*(CH); 2058 s, 1981 m, 1931 vs, 1906 vs *ν*(CO); 1522 w *ν*(C=C), phenyl; 1445 vw, 1423 w, 1406 w, 1306 w, 1248 m *δ*(CH); 1161 vw, 1109 w, 1036 w, 951 w, 901 m, 833 m, 810 m, 777 m, 731 w *ν*(CC), *ρ*(SiCH_3_); 698 m, 656 s *ν*(SiC), phenyl; 527 vw, 513 vw, 455 w *ν*(PC), *δ*(CC). MS (EI, 20 eV, 353 K): *m*/*z* (%) = 676 (1) [M]^+^, 620 (2) [M − 2CO]^+^, 592 (4) [M − 3CO]^+^, 536 (29) [M − 5CO]^+^, 484 (100) [**6**].^+^ Anal. calcd for C_33_H_33_O_5_PSi_3_Cr (676.9): C, 58.6, H, 4.9. Found: C, 58.4; H, 4.8.

### Synthesis of the W complex of **9**

[W(CO)_6_] (0.17 g, 0.45 mmol) was dissolved in 10 mL of THF and irradiated with a mercury vapor lamp for 7 h. The orange solution was added to neat **7** (0.17 g, 0.32 mmol) at room temperature. The mixture was stirred overnight. All volatiles were removed at 40 °C *in vacuo*, and the crude product was purified by column chromatography (SiO_2_; *n*-hexane/Et_2_O 8 : 1). The solvents were removed *in vacuo*, and the product was recrystallised from a saturated mixture in *n*-hexane and Et_2_O at room temperature (0.15 g, 55%). Mp: 160 °C. ^1^H NMR (CDCl_3_, 300 K, 400 MHz): *δ* 7.80 (dd, ^3^*J*_PH_ = 18.2 Hz, ^4^*J*_PH_ = 1.5 Hz, 1H, 7-*H*), 7.44 (overlap, m, 3H, 4*-H* and *o-H* 10-Si-Ph), 7.43 (overlap, m, 3-*H*), 7.30 (overlap, m, 2H, *p-H* 8- and 10-Si-Ph), 7.23 (overlap, m, 5H, 5-*H* and *m-H* 8- and 10-Si-Ph), 7.19 (overlap, d, ^3^*J*_PH_ = 35.0 Hz, 1H, 9-*H*), 7.17 (overlap, m, 2H, *o-H* 8-Si-Ph), 7.08 (t, ^3^*J*_HH_ = 7.4 Hz, 1H, *p-H* P-Ph), 6.81 (*pseudo*-t, ^3^*J*_HH_ = 7.5 Hz, 2H, *m-H* P-Ph), 6.44 (dd, ^3^*J*_PH_ = 9.0 Hz, ^3^*J*_HH_ = 7.6 Hz, 2H *o-H* P-Ph), 0.69 (s, 3H, 10-Si-C*H*_3_^1^), 0.63 (s, 3H, 8-Si-C*H*_3_^1^), 0.51 (s, 3H, 10-Si-C*H*_3_^2^), 0.43 (s, 3H, 8-Si-C*H*_3_^2^). ^13^C{^1^H} NMR (CDCl_3_, 300 K, 101 MHz): *δ* 198.0 (dd, ^2^*J*_PC_ = 19.7 Hz, ^3^*J*_PC_ = 2.1 Hz, W–CO axial), 196.5 (dd, ^2^*J*_PC_ = 6.3 Hz, ^3^*J*_PC_ = 2.6 Hz, W–CO equatorial), 150.8 (s, 9-*C*), 147.6 (dd, ^2^*J*_PC_ = 13.7 Hz, ^3^*J*_PC_ = 6.0 Hz, 7-*C*), 147.0 (dd, ^1^*J*_PC_ = 13.3 Hz, ^2^*J*_PC_ = 4.5 Hz, 10-*C*), 143.5 (dd, ^2^*J*_PC_ = 18.2 Hz, ^3^*J*_PC_ = 2.0 Hz, 2-*C*), 138.7 (dd, ^1^*J*_PC_ = 30.5 Hz, ^2^*J*_PC_ = 3.6 Hz, 1-*C*), 137.2 (d, ^3^*J*_PC_ = 3.7 Hz, *i-C* 10-Si-Ph), 135.7 (d, ^3^*J*_PC_ = 4.1 Hz, *i-C* 8-Si-Ph), 135.5 (dd, ^2^*J*_PC_ = 8.5 Hz, ^3^*J*_PC_ = 3.7 Hz, 6-*C*), 134.4 (s, *o-C* 10-Si-Ph), 134.0 (s, *o-C* 8-Si-Ph), 132.6 (dd, ^2^*J*_PC_ = 22.8 Hz, ^3^*J*_PC_ = 4.3 Hz, *o-C* P-Ph), 132.2 (dd, ^1^*J*_PC_ = 63.4 Hz, ^2^*J*_PC_ = 4.1 Hz, 8-*C*), 130.6 (dd, ^1^*J*_PC_ = 34.4 Hz, ^2^*J*_PC_ = 5.8 Hz, *i-C* P-Ph), 130.3 (s, 4-*C*), 129.9 (s, *p-C* P-Ph), 129.5 (s, *p-C* 10-Si-Ph), 129.2 (s, *p-C* 8-Si-Ph), 128.1 (dd, ^3^*J*_PC_ = 7.8 Hz, ^4^*J*_PC_ = 1.5 Hz, *m-C* P-Ph), 127.8 (s, *m-C* 10-Si-Ph), 127.7 (s, *m-C* 8-Si-Ph), 125.5 (d, ^3^*J*_PC_ = 5.4 Hz, 3-*C*), 124.8 (d, ^3^*J*_PC_ = 6.6 Hz, 5-*C*), −0.3 (s, 10-Si-*C*H_3_^2^), −1.1 (s, 10-Si-*C*H_3_^1^), −2.0 (d, ^3^*J*_PC_ = 5.2 Hz, 8-Si-*C*H_3_^1^), −2.7 (d, ^3^*J*_PC_ = 5.0 Hz, 8-Si-*C*H_3_^2^). ^29^Si{^1^H} NMR (CDCl_3_, 300 K, 79 MHz): *δ* −2.6 (dd, ^2^*J*_PSi_ = 44.7 Hz, ^3^*J*_PSi_ = 12.3 Hz, 8-*Si*), −10.2 (d, ^2^*J*_PSi_ = 21.3 Hz, 10-*Si*). ^31^P{^1^H} NMR (CDCl_3_, 300 K, 162 MHz): *δ* −11.9 (d, ^1^*J*_PP_ = 313.8 Hz, ^1^*J*_WP_ = 211.3 Hz, *P*C_4_ heterocycle, −65.7 (d, ^1^*J*_PP_ = 313.8 Hz, *P*-Ph). IR (KBr, cm^−1^): 3065 vw, 2961 vw *ν*(CH); 2068 m, 1981 w, 1948 vs, 1923 sh, 1911 vs *ν*(CO); 1659 vw, 1562 vw *ν*(C=C, phenyl; 1425 vw, 1300 vw, 1248 w *δ*(CH); 1157 vw, 1111 w, 1038 vw, 999 vw, 959 vw, 876 w, 833 w, 810 w, 779 w, 729 w *ν*(CC), *ρ*(SiCH_3_); 694 w, 652 vw *ν*(SiC), phenyl; 594 w, 577 w, 519 w, 463 vw *ν*(PC), *δ*(CC). MS (EI, 20 eV, 403 K): *m*/*z* (%) = 858 (1) [M]^+^, 830 (62) [M − CO]^+^, 802 (5) [M − 2CO]^+^, 774 (50) [M − 3CO]^+^, 746 (46) [M − 4CO]^+^, 718 (58) [M − 5CO]^+^, 534 (100) [**7**]^+^. Anal. calcd for C_37_H_32_O_5_P_2_Si_2_W (858.6): C, 51.8, H, 3.8. Found: C, 51.6; H, 3.8.

### Synthesis of **10**

**3a** (0.14 g, 0.33 mmol) was dissolved in 20 mL of *n*-pentane and cooled to −78 °C. A solution of H_2_O_2_ in H_2_O (35.5%, 28 μL, 0.32 mmol) was added. The mixture was slowly warmed to room temperature overnight. All volatiles were removed *in vacuo*, and the residue was dissolved in a mixture of dichloromethane and diethyl ether. The solution was concentrated and cooled to 7 °C to afford **10** as colourless crystals (0.12 g, 79%). Mp: 124 °C. ^1^H NMR (C_6_D_6,_ 300 K, 400 MHz): *δ* 12.55 (s br., 1H, P–O*H*), 8.15 (d, ^3^*J*_HH_ = 18.9 Hz, 1H, 7-*H*), 7.61 (m, 2H, *o-H* 10-Si-Ph), 7.54 (m, 2H, *o*-H 8-Si-Ph), 7.33 (dd, ^3^*J*_HH_ = 8.0 Hz, ^4^*J*_PH_ = 5.4 Hz, 1H, 5-*H*), 7.23 (overlap, m, 3H, *m-H* and *p-H* 10-Si-Ph), 7.18 (overlap, m, 3H, *m-H* and *p-H* 8-Si-Ph), 6.93 (d, ^3^*J*_PH_ = 51.6 Hz, 1H, 9-*H*), 6.90 (td, ^3^*J*_HH_ = 7.6 Hz, ^5^*J*_PH_ = 1.3 Hz, 1H, 4-*H*), 6.75 (d, ^3^*J*_HH_ = 18.9 Hz, 1H, 8-*H*), 6.41 (dd, ^3^*J*_HH_ = 7.3 Hz, ^4^*J*_PH_ = 2.9 Hz, 3-*H*), 0.72 (s, 6H, 10-Si-C*H*_3_), 0.46 (s, 6H, 8-Si-C*H*_3_). ^13^C{^1^H} NMR (C_6_D_6_, 300 K, 101 MHz): *δ* 153.0 (d, ^2^*J*_PC_ = 13.1 Hz, 9-*C*), 141.6 (d, ^2^*J*_PC_ = 45.3 Hz, 2-*C*), 141.6 (d, ^3^*J*_PC_ = 5.4 Hz, 7-*C*), 140.6 (d, ^2^*J*_PC_ = 8.4 Hz, 6-*C*), 139.1 (s, *i-C* 8-Si-Ph), 137.0 (d, ^3^*J*_PC_ = 4.3 Hz, *i-C* 10-Si-Ph), 136.3 (d, ^1^*J*_PC_ = 89.6 Hz, 10-*C*), 134.7 (s, *o-C* 10-Si-Ph), 134.3 (s, *o-C* 8-Si-Ph), 133.6 (s, 8-*C*), 133.0 (d, ^4^*J*_PC_ = 1.9 Hz, 4-*C*), 129.7 (s, *p-C* 10-Si-Ph), 129.2 (s, *p-C* 8-Si-Ph), 128.7 (overlap, d, ^1^*J*_PC_ = 125.5 Hz, 1-*C*), 128.3 (overlap, s, *m-C* 10-Si-Ph), 128.1 (s, *m-C* 8-Si-Ph), 126.3 (d, ^3^*J*_PC_ = 8.8 Hz, 5-*C*), 124.1 (d, ^3^*J*_PC_ = 13.2 Hz, 3-*C*). ^29^Si{^1^H} NMR (C_6_D_6_, 300 K, 79 MHz): *δ* −10.2 (d, ^2^*J*_PSi_ = 14.8 Hz, 10-*Si*), −10.5 (s, 8-*Si*). ^31^P (^1^H) NMR (C_6_D_6_, 300 K, 162 MHz): *δ* 62.8 (s). IR (KBr, cm^−1^): 3431 vw *ν*(OH); 3162 vw, 3065 m, 3049 m, 3011 w, 2955 m, 2895 w *ν*(CH); 2583 vw, 2286 w, 2156 vw; 1954 vw, 1883 vw, 1819 vw, 1794 vw, 1769 vw, 1651 w, 1614 w, 1591 w, 1562 m, 1539 s *ν*(C=C), phenyl; 1485 w, 1456 s, 1425 s, 1412 m, 1379 vw, 1333 vw, 1317 w, 1261 vs, 1250 vs *δ*(CH); 1219 w, 1204 m, 1188 w, 1152 s, 1113 vs, 1045 w, 1007 vs, 991 vs, 953 vs, 837 vs, 810 vs, 804 vs, 779 vs, 737 vs, 700 vs *ν*(CC), *ρ*(SiCH_3_), *δ*(OH); 677 m, 658 m, 635 m, 611 m *ν*(SiC), phenyl; 575 m, 540 w, 511 vs, 469 s, 430 w *ν*(PC), *ν*(PO), *δ*(CC). MS (EI, 20 EV, 393 K): *m*/*z* (%) = 459 (47) [M − H]^+^, 382 (100) [M − Ph − H]^+^, 367 (20) [M − CH_3_ − Ph − H]^+^. Anal. calcd for C_26_H_29_O_2_PSi_2_ (460.7): C, 67.8, H, 6.3. Found: C, 67.6; H, 6.2.

### X-ray crystallography

Crystals suitable for X-ray crystallography were obtained by crystallisation from toluene (**5**, 3 °C), *n*-hexane (**7**, room temperature), a mixture of diethyl ether/*n*-hexane (**9**, room temperature) and CH_2_Cl_2_/Et_2_O (**10**, 7 °C). Intensity data was collected on a Bruker D8 Venture diffractometer with monochromated Mo K_α_ radiation. The collection method involved *ω*-scans. Data reduction was carried out using the program *SAINT*^*+*^.^41^ The crystal structures were solved by direct methods using *SHELXTL*.^42^ Non-hydrogen atoms were first refined isotropically followed by anisotropic refinement by full matrix least-squares calculation based on *F*^2^ using *SHELXTL*.^42^ H atoms were positioned geometrically and allowed to ride on their respective parent atoms. **5** crystallizes with two molecules of toluene per dimer. The P_2_C_4_ rings of **7** were disordered; the atoms of the molecular centre were refined on split positions (0.72 : 0.28). A further minor disorder led to the superposition of PC_4_ and P_2_C_4_ rings; the P atoms of the minor component were refined with site occupancy factors of 0.036 and 0.023. The crystals of **10** enclosed a molecule of CH_2_Cl_2_; its Cl atoms were refined on split positions (0.66 : 0.34).

## Conflicts of interest

There are no conflicts to declare.

## Supplementary Material

SC-012-D0SC06155G-s001

SC-012-D0SC06155G-s002
