# Supplementary material for: Sterically constrained tricyclic phosphine: redox behaviour, reductive and oxidative cleavage of P–C bonds, generation of a dilithium phosphaindole as a promising synthon in phosphine chemistry
Source: Chem Sci. 2021 Jan 18;12(10):3460–74. doi: 10.1039/d0sc06155g (PMC8179454; doi:10.1039/d0sc06155g)
Supplement: SC-012-D0SC06155G-s001 [file SC-012-D0SC06155G-s001.pdf]

Electronic Supplementary Material (ESI) for Chemical Science.
This journal is © The Royal Society of Chemistry 2021

Sterically constrained tricyclic phosphine: Redox behaviour, reductive and oxidative cleavage of P-C bonds, generation of a dilithium phosphaindole as a promising synthon in phosphine chemistry

Alexander Brand,[a] Stephen Schulz,[b] Alexander Hepp,[a] Jan J. Weigand,*[,b] and Werner Uhl*[, a]

Supporting Information

1 Electrochemical and spectroelectrochemical data 2
1.1 General electrochemical procedures 2
1.2 Cyclic voltammetry data of 3a in PhF, CH_3CN and THF 3
1.3 Additional *in situ* UV-vis spectroelectrochemical data and mechanistic investigation of the reduction of 3a 6
1.4 Preparative electrochemical reduction of phosphine 3a 8
2 ^{1}H, ^{13}C, ^{31}P and ^{7}Li NMR spectra 11
2.1 NMR-Spectra of Compound 5 11
2.2 NMR-Spectra of Compound 6 13
2.3 NMR-Spectra of Compound 7 15
2.4 NMR-Spectra of Compound 8 17
2.5 NMR-Spectra of Compound 9 18
2.6 NMR-Spectra of Compound 10 20
3 Quantum Chemical Calculations 22
3.1 Optimized Structure of 5 in Gas Phase 22
3.2 Structure of 5 with Dummy Atom for NICS(0) Calculation 24
3.3 Optimized Structure of 7 (S,R) with Solvent-Correction 26
3.4 Optimized Structure of 7 (S,S) with Solvent-Correction 28
3.5 Optimized Structure of Phosphoric Acid as Reference for NMR Calculations 31
3.6 Optimized Structure of 7 (S,R) in Gas Phase 32

3.7 Optimized Structure of 7 (S,S) in Gas Phase 34
3.8 Transition-State of Configurational Inversion of 7 at P-Ph in Gas Phase 37
3.9 Transition-State of Configurational Inversion of 7 at PC_4 in Gas Phase 39
3.10 Input Line for NMR Calculation of NICS(0) of 5 42
3.11 Input Line for NMR Calculation of Phosphoric Acid 42
3.12 Input Line for NMR Calculation of 7 (S,R) 42
3.13 Input Line for NMR Calculation of 7 (S,S) 42

1 Electrochemical and spectroelectrochemical data

1.1 General electrochemical procedures

All electrochemical (*EC*) and *in situ* UV-vis spectroelectrochemical (*in situ SEC*) measurements were performed in a Glovebox Pure Lab HE GP-1 SR (Innovative Technology, USA) within an atmosphere of purified nitrogen (<0.1ppm O_2; <0.1ppm H_2O). The glovebox was equipped with military grade BNC feedthroughs in a homemade gas tight flange for low noise electrical connection of electrochemical and spectroelectrochemical cells inside. UV-vis-NIR spectroscopy inside the glovebox was performed *via* another homemade, gas tight flange containing four light-tight chambers with optical grade fused silica windows and four 74-UV (Ocean Optics Inc., USA) collimating lenses with SMA 905 connectors for optical fibers. EC and SEC cells were connected to a PGSTAT302 (Metrohm Autolab, Utrecht, The Netherlands) E= ±10 V, U= ±35 V with an auxiliary voltage monitor (BK Precision 2831E, Yorba Linda, CA USA). NOVA Software (Metrohm Autolab) Version 1.11.2 was used to control the potentiostat, magnetic stirring and to trigger the spectrometer. UV-vis spectra recorded by OceanView software (Version 1.4.1, Ocean Optics) were triggered by a DG1032Z arbitrary waveform function generator (Rigol, Beijing, China) controlled by the Nova Software (1.10.4, Fa Metrohm Autolab) using a DAC164 analog/digital converter. Data analysis of the spectral and electrochemical data was carried out using OriginPro 2019 (OriginLab Cooperation, Northampton, MA, USA).

Solvent preparation / supporting electrolyte preparation

THF was dried by refluxing with potassium and benzophenone as an indicator until a midnight blue color was established, CH_3CN and PhF were dried by heating with CaH_2 to reflux conditions for at least 6 h and distillation under Argon onto vacuum activated molecular sieves (CH_3CN: 3 Å ; PhF, THF: 4 Å, $1\cdot10^{-3}$ mbar, 350 °C, 12 h). Additionally, all solvents were stored over vacuum activated molecular sieves for at least 14 days. [^{n}Bu$_4$N][OTf] was pre-dried at least 5 times by dissolving in CH_2Cl_2 and evaporating the solvent to high vacuum at 80 °C. Final drying and removal of HOTf traces was achieved by dissolving in dry benzene and refluxing this solution in a soxleth apparatus for 5 days with vacuum activated molecular sieves in the extraction thimble renewed every day. Prior to each measurement the solvent is passed through a Pasteur pipette with an activated ($1\cdot10^{-3}$ mbar, 350 °C, 24 h) aluminum oxide bed (*D* = 5 mm; *L* = 70 mm) in the glove box before the supporting electrolyte is added.

1.2 Cyclic voltammetry data of 3a in PhF, CH_3CN and THF

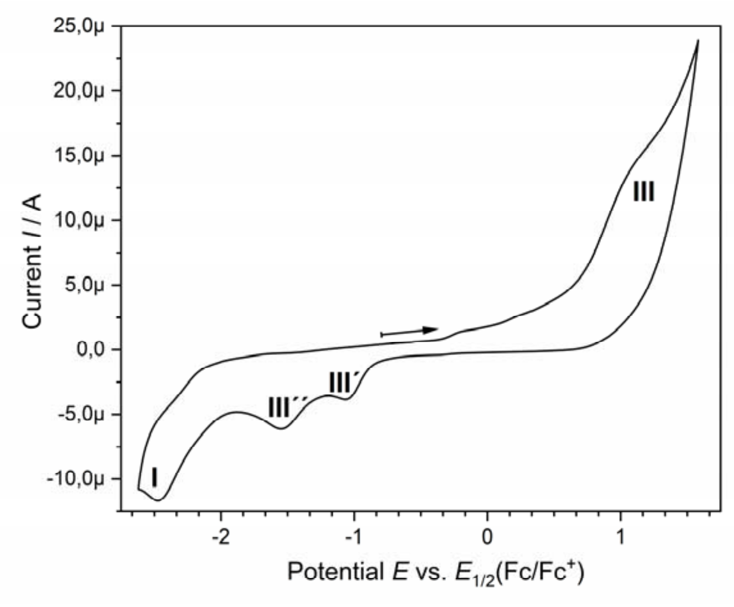

Figure SI.1. Cyclic voltammograms (2nd cycle; v = 0.1 V/s) of phosphine **3a** (3.94 mM) in PhF / 0.1 M [^{n}Bu$_4$N][OTf] at a D = 3 mm platinum disc electrode.

In PhF as a solvent a substrate-based non-reversible reduction **I** at E_P(red) = -2.62 V (vs. $E_{1/2}$(Cp_2Fe(Cp_2Fe^+))) and an non-reversible oxidation **II** at E_P(ox) = 1.12 V were observed as broad peaks in the CV (Figure SI.1.). Square wave voltammetry revealed half-wave potentials according to the CV at $E_{1/2}$(red) = -2.75 V for the reduction and $E_{1/2}$(red) = 0.72 V for the oxidation reaction. Follow-up reactions are observed after the oxidation reaction **III** in the second cycle as non-reversible re-reduction peaks **III´** and **III´´** at E_P(re-red) = -1.07 V and E_P(re-red) = -1.55 V. Therefore, PhF is not usable as solvent for further spectroelectrochemical investigations due to a low diffusion coefficient of **3a** in the reduction reaction **I** and the follow-up reactions **III´´** and **III´´´** after the substrate oxidation **III**.

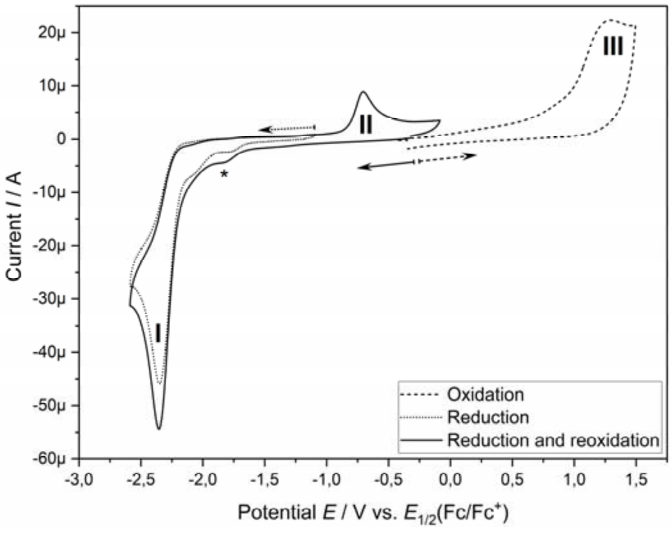

Figure SI 2. Cyclic voltammogram of **3a** (3.05 mM) in CH_3CN / 0.1 M [^{n}Bu$_4$N][OTf] at a Pt disc electrode (D = 3 mm; 0.1 V/s) with separated reduction and oxidations cycles. Asterisks mark minor impurities.

In CH_3CN as a solvent phosphine **3a** showed a reasonable sharp non-reversible reduction **I** peak E_P(red) = -2.36 V (vs. $E_{1/2}$(Cp_2Fe(Cp_2Fe^+); Figure SI.2. dotted curve) attributed to the two-electron reduction mechanism of **3a** to [**3a'**]$^{2-}$. The non-reversible oxidation **III** was observed at E_P(ox) = 1.28 V

(dashed line). Additionally, extended CV measurements of the reduction reaction **I** show the known two-electron re-oxidation **II** (solid line) of dianion [**3a'**]$^{2-}$ to **3a'** and back to **3a** (*vide infra*) at E_P(re-ox) = -0.7 V. SWV measurements allow an assignment of the half-wave potentials $E_{1/2}$(SWV) = -2.38 V to the substrate-based reduction **I** and $E_{1/2}$(SWV) = 1.15 V to substrate-based oxidation **II**.

Further investigation of the substrate based reduction **I** and the substrate based oxidation reaction **II** were conducted by a comparison of the full potential window CVs (dashed line) versus the partial CV scan for the oxidation follow-up processes (Figure SI.3., solid line), as well as for the reduction follow-up processes (Figure SI.4., solid line).

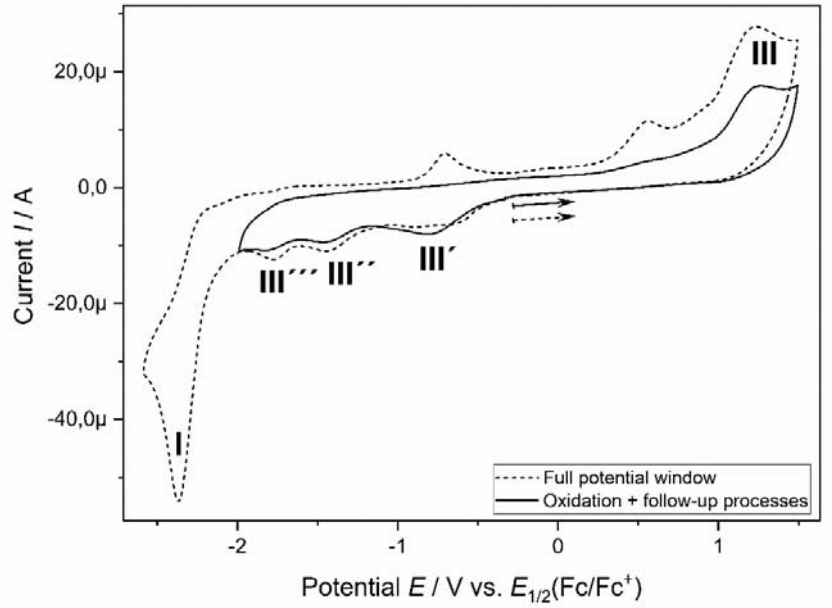

Figure SI.3. Different potential (oxidation) scans of the CV (2nd cycle; v = 0.1 V/s) of phosphine **3a** (3.05 mM) in CH_3CN / 0.1 M [^{n}Bu$_4$N][OTf] at a D = 3 mm platinum disc electrode to assign processes of the full potential window cycle to follow-up reduction processes **III´- III´´´** of the substrate oxidation process **III**.

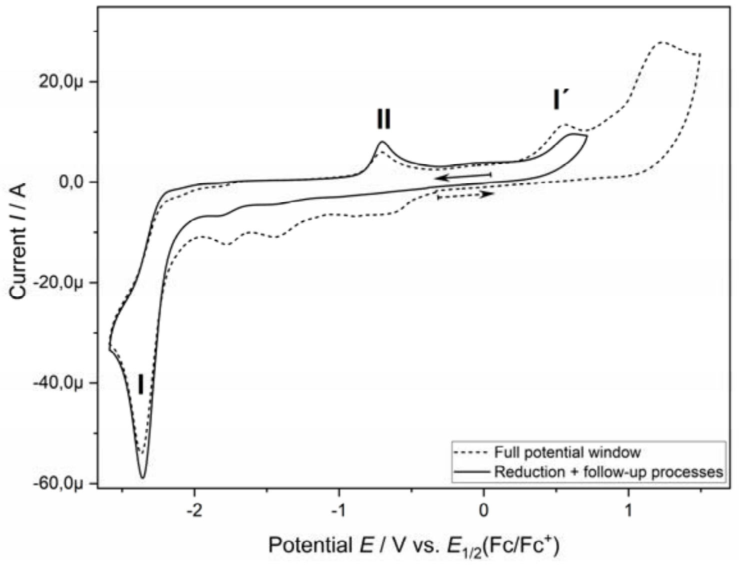

Figure SI.4. Different potential (reduction) scans of the CV (2nd cycle; v = 0.1 V/s) of phosphine **3a** (3.05 mM) in CH_3CN / 0.1 M [^{n}Bu$_4$N][OTf] at a D = 3 mm platinum disc electrode to assign processes of the full potential window cycle to follow-up oxidation processes **II** and **I´** of the substrate reduction process **I**.

Unfortunately, the oxidation **III** of phosphine **3a** leads to three follow-up reduction reactions **III´ / III´´ / III´´´** E_P(red) = -0.79 / -1.44 / -1.81 V indicating unwanted reactivity of the oxidation product in acetonitrile (Figure SI.3.). In acetonitrile an additional re-oxidation reaction **I´** E_P(ox) = 0.55 V (Figure SI.4.) after the already assigned re-oxidation reaction **II** is also found, rendering acetonitrile as not suitable solvent to handle the two-electron reduction (**I**) product [**3a'**]$^{2-}$ due to further reactions. Based on this detailed peak assignment acetonitrile is not suitable for the reduction of phosphine **3a** although the potential range of CH_3CN (in blank measurements) lead to another prediction.

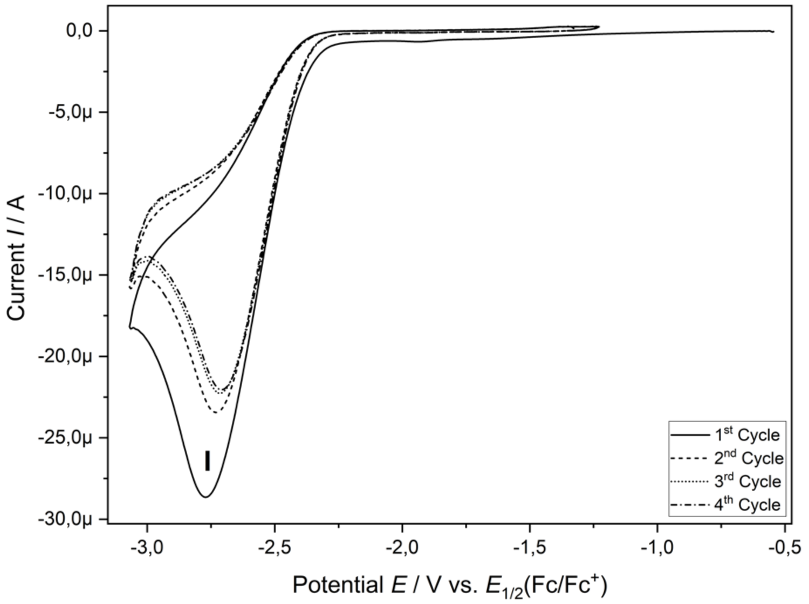

Figure SI.5. CV measurement of the reduction only (1st to 4th cycle; v = 0.1 V/s) of phosphine **3a** (2.32 mM) in THF / 0.1 M [^{n}Bu$_4$N][OTf] at a D = 3 mm platinum disc electrode. iR Compensated by R = 4750 Ω.

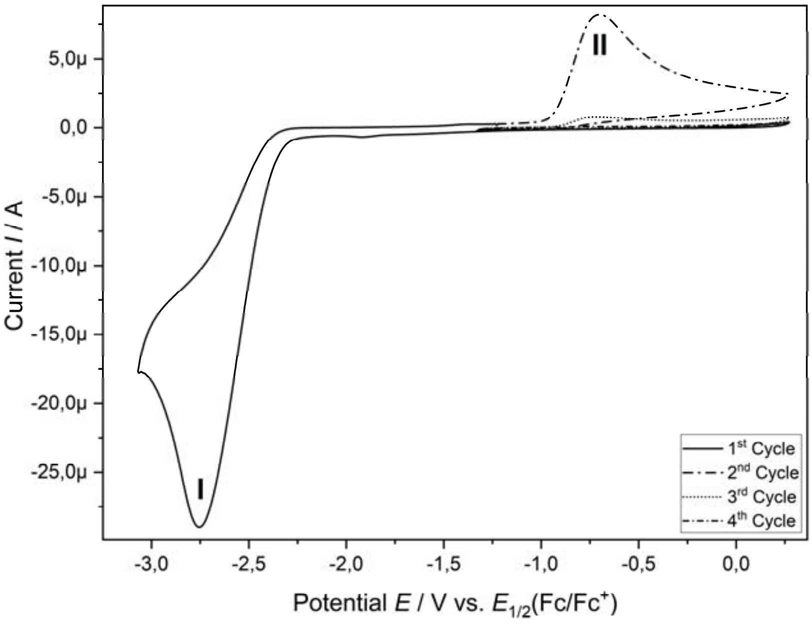

Figure SI.6. CV measurement of the reduction **I** in the first cycle and following potential cycling around the reoxidation **II** range [-1.33 – 0.27 V] (2nd to 4th cycle; v = 0.1 V/s) of phosphine **3a** (2.32 mM) in THF / 0.1 M [^{n}Bu$_4$N][OTf] at a D = 3 mm platinum disc electrode. iR Compensated by R = 4750 Ω.

CV measurements in THF do not allow for the observation of the oxidation reaction due to the limited oxidative potential window, but the reductive stability avoid side reactions after multiple reduction **I** cycles (Figure SI.5.). This enables an exhaustive re-oxidation **II** after the reduction product [**3a’**]$^{2-}$ is formed (Figure SI.6.). Figure SI.5. shows a CV measurement cycling exclusive around the non-reversible reduction peak **I**. No changes starting from the second cycle are observed is in accordance with the mechanism hypothesis of the two-electron reduction followed by a very fast chemical follow-up reaction forming [**3a’**]$^{2-}$ without the formation of electro-active side products: **3a** + 2 e$^-$ → [**3a’**]$^{2-}$ (*EEC* mechanism, see manuscript). Additionally, the back reaction from dianion [**3a’**]$^{2-}$ to **3a** via an *EEC* mechanism with a slower chemical step. Investigation by composited CV cycles (Figure SI.6.). producing the two-electron reduction **I** product [**3a’**]$^{2-}$ in a first cycle followed multiple cycles to show the exhaustive properties of the re-oxidation **II** due to limited amounts of [**3a’**]$^{2-}$ in the diffusion layer from the first cycle.

1.3 Additional *in situ* UV-vis spectroelectrochemical data and mechanistic investigation of the reduction of 3a

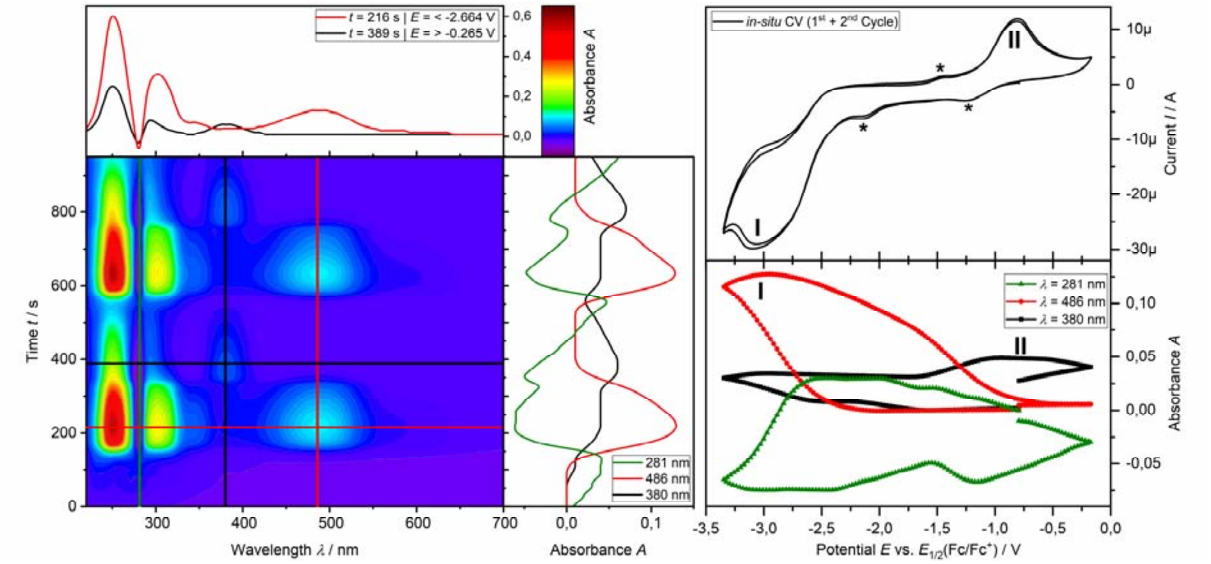

Figure SI.7. Spectroelectrochemical CV measurement of the reduction **I** and reoxidation **II** (v = -15 mV/s) of phosphine **3a** (0.84 mM) in THF / 0.1 M [^{n}Bu$_4$N][OTf] at a platinum grid electrode. Left contour plot: Relative UV-vis spectral changes during 2 cycles of CV; middle vertical plot: time dependent UV-vis absorption; top left: Projected relative UV-vis spectra after reduction **I** (red line) and after reoxidation **II** (black line); top right: *in-situ* CV, bottom right: absorption based CV. Asterisks mark minor impurities.

Spectroelectrochemical *in situ* UV-vis CV measurement Figure 2. (Manuscript) is performed in a high-concentration regime and allows to acquire suitable UV-vis spectra accompanied with a negative effect on the peak shapes. The used concentration (2.50 mM) was chosen to allow for a half-life time determination (*vide infra*). Figure SI.7. depicts the UV-vis CV with a concentration of 0.84 mM for phosphine **3a** leading to peak shapes for reduction **I** and re-oxidation **II** in accordance to the CV data (*vide supra*). The UV-vis spectra detect only traces of the intermediate **3a’** (black line, top left and black line, bottom right) formed after [**3a’**]$^{2-}$ (red line, bottom right) is re-oxidized (**II**). The reformation of phosphine **3a** is observed by its characteristic absorption (green line, bottom right).

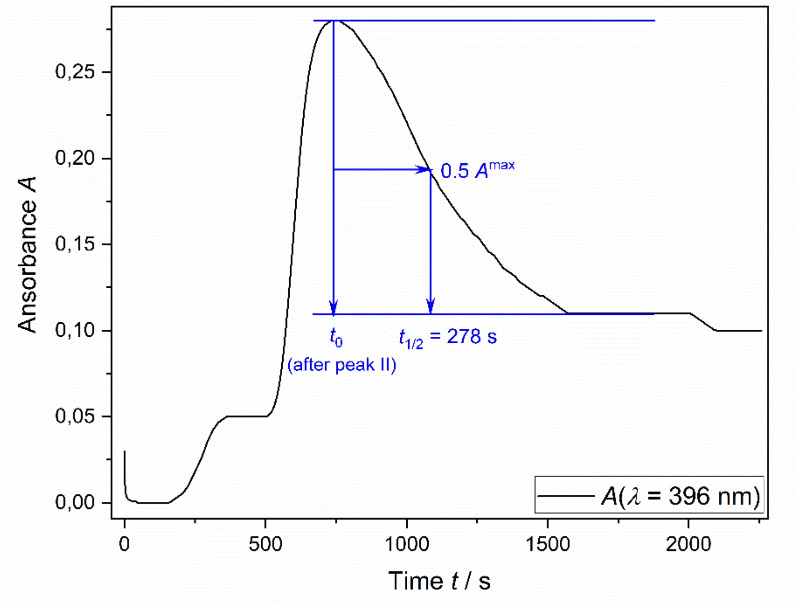

Figure S8. Absorbance during the spectroelectrochemical CV measurement (see manuscript Fig. 4) of the characteristic wavelength attributed to intermediate **3a'** and half-life time determination after re-oxidation UV-vis peak **II** (v = -10 mV/s). Phosphine **3a** (2.50 mM) in THF / 0.1 M [^{n}Bu$_4$N][OTf] at a platinum grid electrode in a double-compartment cuvette-cell.

Half-life time measurement of intermediate **3a'** based on a composed UV-vis CV experiment (Figure 4, manuscript) after reduction **I** of **3a** to [**3a'**]$^{2-}$ the time after the re-oxidation peak in the UV-vis experiment is used for a half-life time determination (Figure S8.). Assuming the electrochemical formation of intermediate **3a'** has fully proceed at the maximum of its characteristic UV-vis absorption.

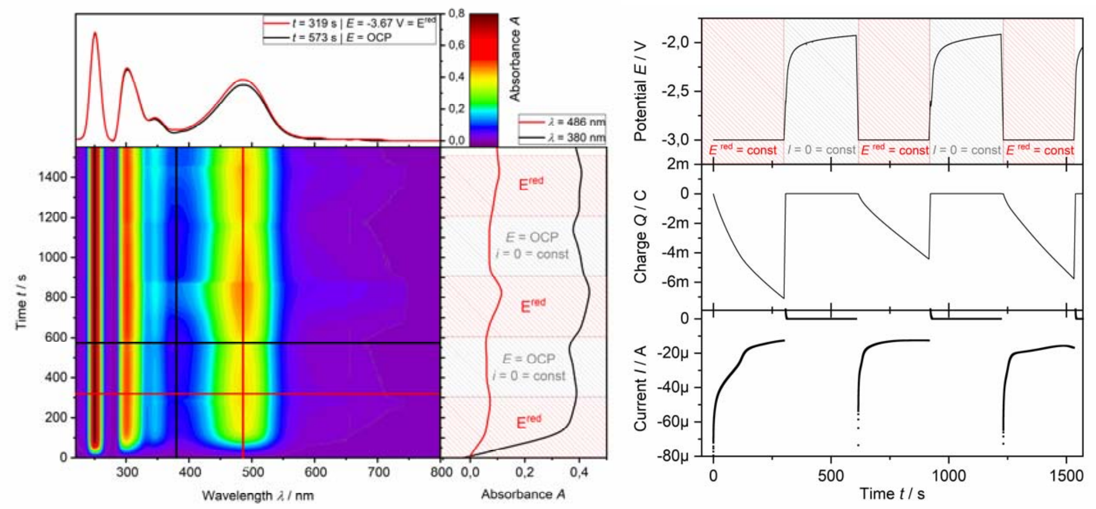

Figure S9. *In situ* UV-vis multipulse chrono amperometry / potentiometry (*in situ* UV-vis-MPCACP) of phosphine **3a** (2.50 mM) in THF / 0.1 M [^{n}Bu$_4$N][OTf] at a platinum grid electrode in a double compartment cuvette cell (d = 0.5 mm). Left, bottom: 2D-Plot of relative *in situ* UV-vis spectra during the MPCACV measurement; left, top: UV-vis spectra after the reduction pulse (red line) and after the zero-current pulse (black line); middle vertical: Relative UV-vis absorbance during reduction and zero-current pulses; right top to bottom: chronopotentiogramm, chronocoulombgramm, chronoamperogramm.

In addition to the *in situ* UV-vis MPCA experiment (Figure 5, manuscript) a background experiment was designed in order to differentiate between diffusion and re-oxidation of dianion [**3a'**]$^{2-}$ to **3a**. Therefore, a chronoamperametric pulse to trigger the reduction **I** reaction **3a** → [**3a'**]$^{2-}$ was applied, followed by a chronopotentiometric pulse controlling the current to zero. During the

chronopotentiometric pulse no reduction or re-oxidation happened. Only diffusion effect from the thin-layer area to the bulk compartment of the double-compartment cuvette-cell can take place. The losses by diffusion effects during the time with zero current conditions show only slight changes on the concentration of [**3a'**]$^{2-}$ in the cuvette cell. According to this finding, the reduction **I** and re-oxidation **II** in the *in situ* UV-vis MPCA measurement Figure 4 (manuscript) is suitable to proof the stability, spectroscopic and chemical reversibility of the reactions between **3a** and [**3a'**]$^{2-}$.

In addition to the half-life time measurement of the intermediate **3a´** for the *EEC* reoxidation-mechanism ([**3a'**]$^{2-}$ → **3a**) the *EEC* reduction-mechanism (**3a** → [**3a'**]$^{2-}$) was further investigated. The *EEC* hypothesis of the reduction is given by a CV experiment with an elevated scan rate of v = 10 V/s (Figure SI10). The reduction peak **I** has significantly broadened but remains up to 10 V/s non-reversible. Separation of the *EE* and *C* part of the mechanism was not possible due to the fast chemical follow-up reaction.

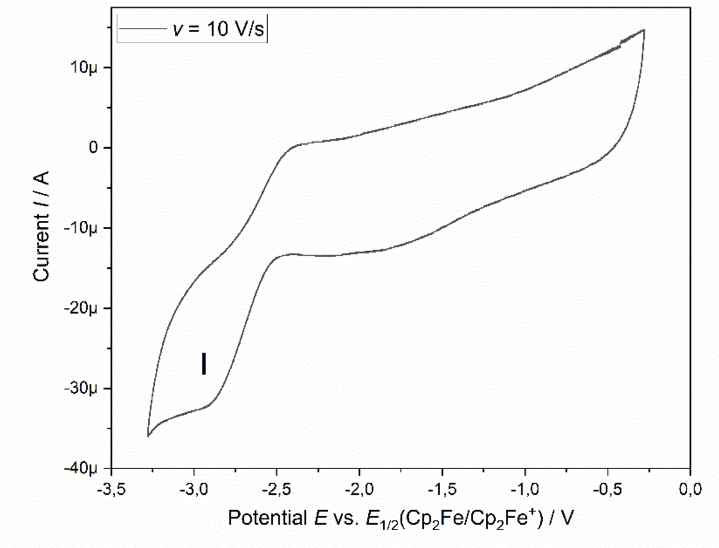

Figure SI10. CV measurement of the (2nd cycle; v = 10 V/s) of phosphine **3a** (4.25 mM) in THF / 0.1 M [^{n}Bu$_4$N][OTf] at a D = 3 mm platinum disc electrode. No *iR* compensation.

1.4 Preparative electrochemical reduction of phosphine 3a

Preparative electrochemical synthesis was used to gain UV-vis data of the reduction product dianion [**3a´**]$^{2-}$ on a preparative scale. Based on the assumed low diffusion coefficient deduced from the CV data potentiostatic conditions were chosen. Therefore, a solution of 0.28 mmol phosphine **3a** in 35 mL THF with 0.1 M [^{n}Bu$_4$N][OTf] as supporting electrolyte was used in the working electrode compartment of a H-type Schlenk electrolysis cell (Fa. HMTC GmbH, Drensteinfurt, Germany) divided by two D4 glass sinter plates. A platinum sheet metal (25*25 mm) is used as a working electrode together with 3 mm Pt disc electrode for *in situ* CV measurements. The counter electrode is based on a graphite rod (H*D = 35*15 mm) in 15 mL THF electrolyte, separated by another 5 mL electrolyte in the ion bridge compartment.

Electrolyses was performed potentiostatic with E = -3.00 V at room temperature insight a nitrogen filled glove box. Figure SI11 shows the current over time and after integration the charge consumption. After 60 min of electrolysis time an *in situ* CV was measured by switching from the preparative to the analytical Pt disc electrode. The potentiostatic synthesis was stopped after 20 h to avoid contamination from the counter electrode compartment. Due to the low current a conversion of only 34% based on a two-electron process with 100% current yield was achieved.

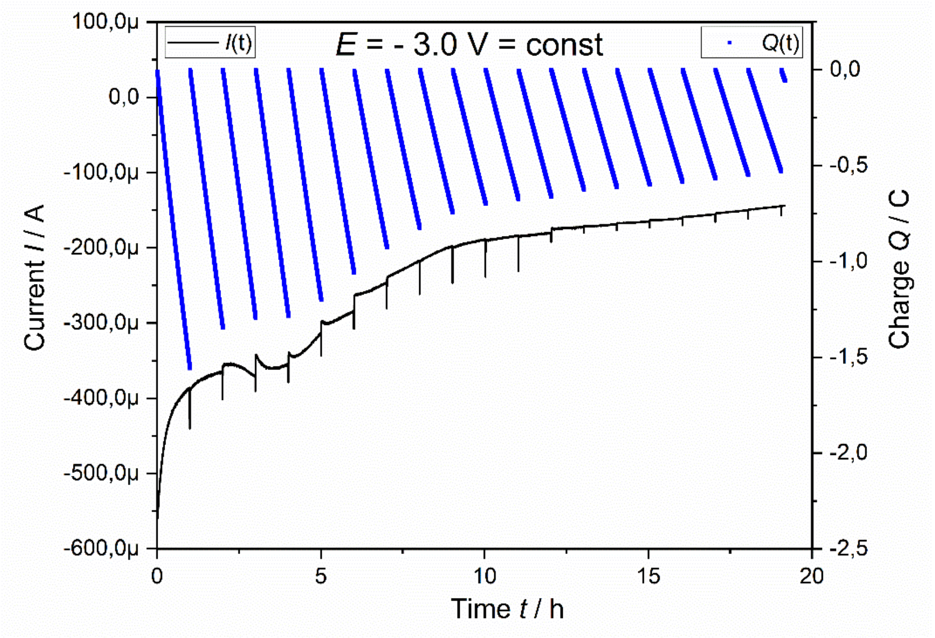

Figure SI11. Current and charge during the potentiostatic electrolysis of phosphine **3a** (8.00 mM) in THF / 0.1 M [^{n}Bu$_4$N][OTf] at a platinum sheet working electrode (25*25 mm) in a separated H-type cell. Samples were taken every 60 min.

In situ CV before the electrolysis (Figure SI12) show a very broad peak **I** for the reduction process, due to the high concentration of phosphine **3a** in the preparative electrolysis cell. The reoxidation peak **II** also showing a reasonable broadening and low intensity. After 23% conversion and a drastic color change from yellow to deep red (insert figure SI13) the CV show a significant increase of the reoxidation peak **II** due to the formed bulk concentration of [**3a**´]$^{2-}$ from the preparative reduction. Further low intensity peaks (*) indicate side reactions between decompositions products from the counter electrode compartment caused by the prolonged electrolysis time.

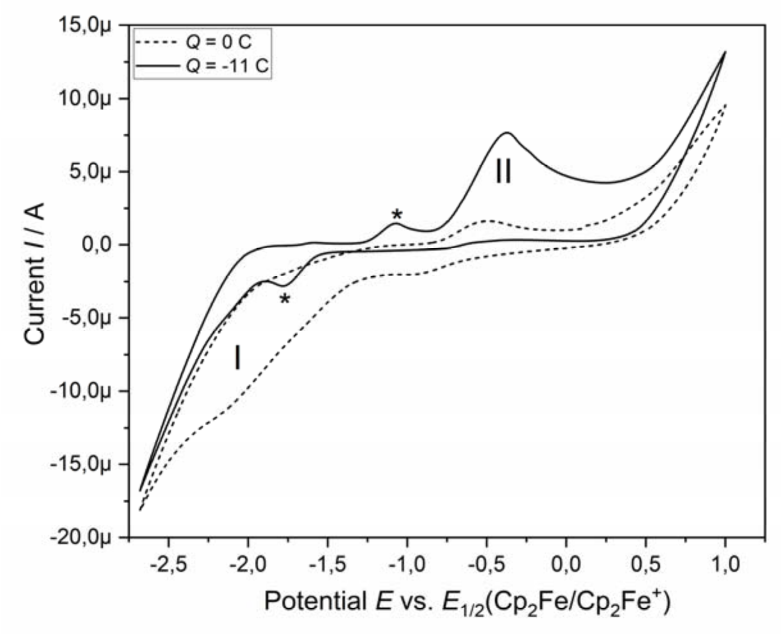

Figure SI12. *In situ* CV measurement of the (2nd cycle; v = 0.1 V/s) of phosphine **3a** (8.00 mM) in THF / 0.1 M [^{n}Bu$_4$N][OTf] at a D = 3 mm platinum disc electrode during the potentiostatic electrolysis in the beginning (Q = 0 C; 0% conversion) and after 20 h (Q = -11.0 C; 22.8% conversion, n = 2 e$^-$) No iR compensation. External ferrocen reference of the RE after the electrolysis.

UV-vis NIR spectroscopy (Figure SI12, black line) indicate the comparable vis band to the *in situ* UV-vis CV experiment (Fig. 2, manuscript) at λ = 485 nm assigned to dication [**3a´**]$^{2-}$. While remeasuring the separated solution after storage at -30 °C under inert conditions a significant decomposition is observed in the UV-vis spectrum (Figure SI12, red line).

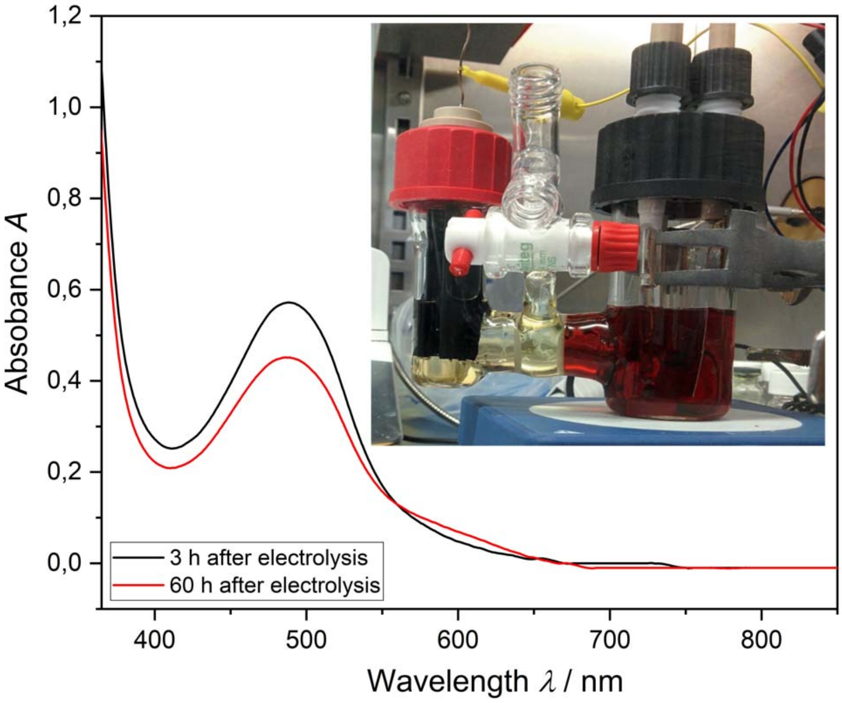

Figure SI13. UV-vis NIR spectra of the working electrode solution (1:10 diluted in 0.1 M [^{n}Bu$_4$N][OTf], d = 1 cm) after 34,0% conversion (Q = -16.0 C; n = 2 e$^-$) and after additional 60 h stored at -30 °C. Insert: Photo of the double separated electrolysis cell (left CE, right WE compartment).

Attempts to isolate and crystalize dianion [**3a´**]$^{2-}$ as its tetrabutyl ammonium salt [^{n}Bu$_4$N]$_2$[**3a´**] from the electrolyte failed due to the high supporting electrolyte content or the decomposition taking place. This support the necessity of the use of lithium-based reduction reagents to further stabilize the dianion [**3a´**]$^{2-}$ be formation of a Li–C bond.

2 ^{1}H, ^{13}C, ^{31}P and ^{7}Li NMR spectra

2.1 NMR-Spectra of Compound 5

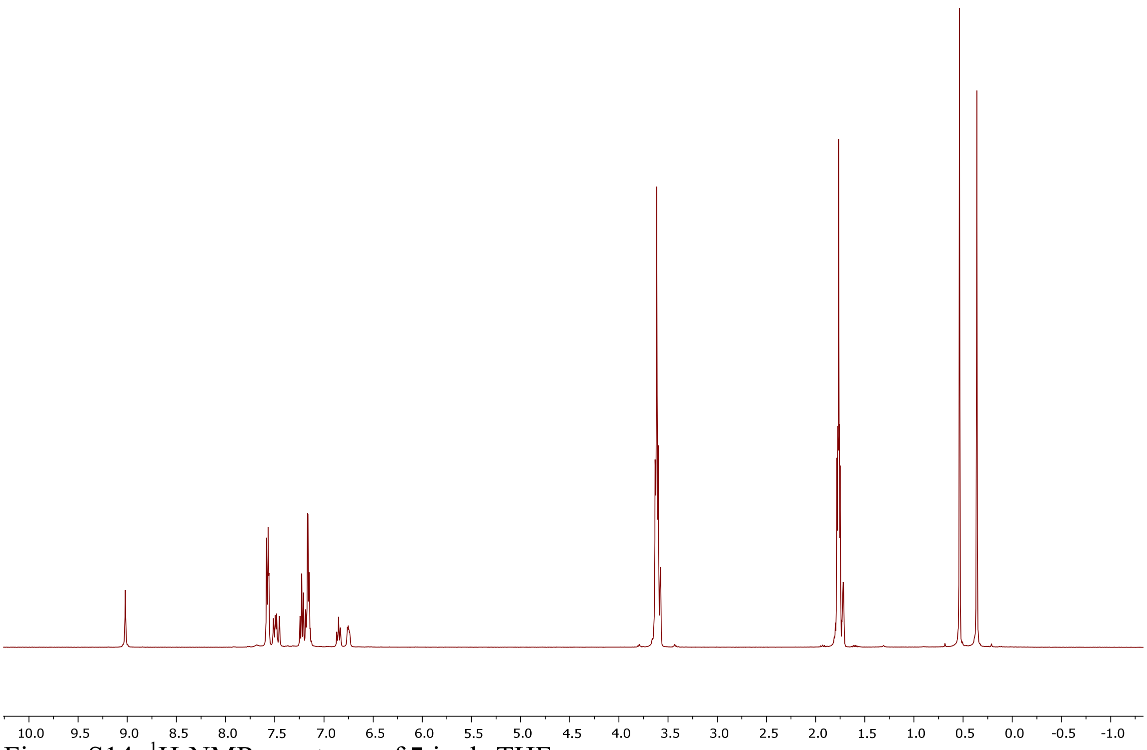

Figure S14: ^{1}H-NMR spectrum of **5** in d_8-THF

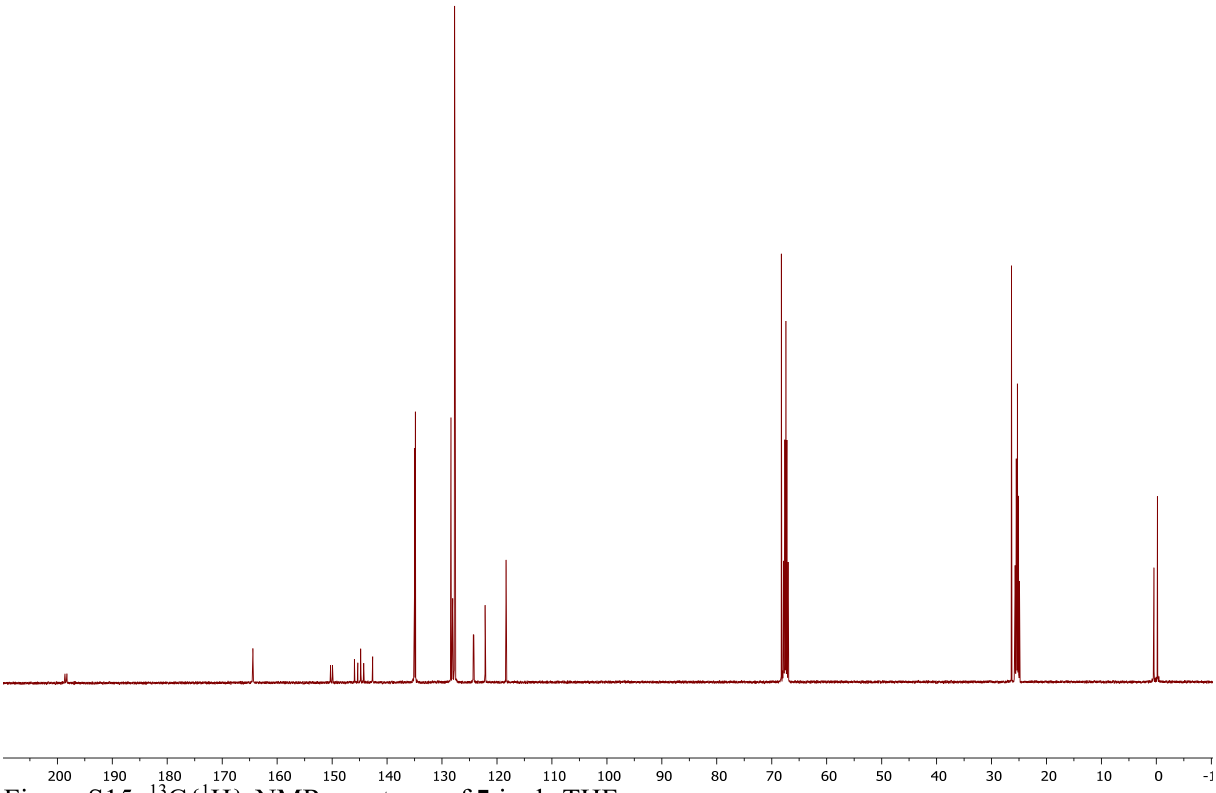

Figure S15: $^{13}C\{^{1}H\}$-NMR spectrum of **5** in d_8-THF

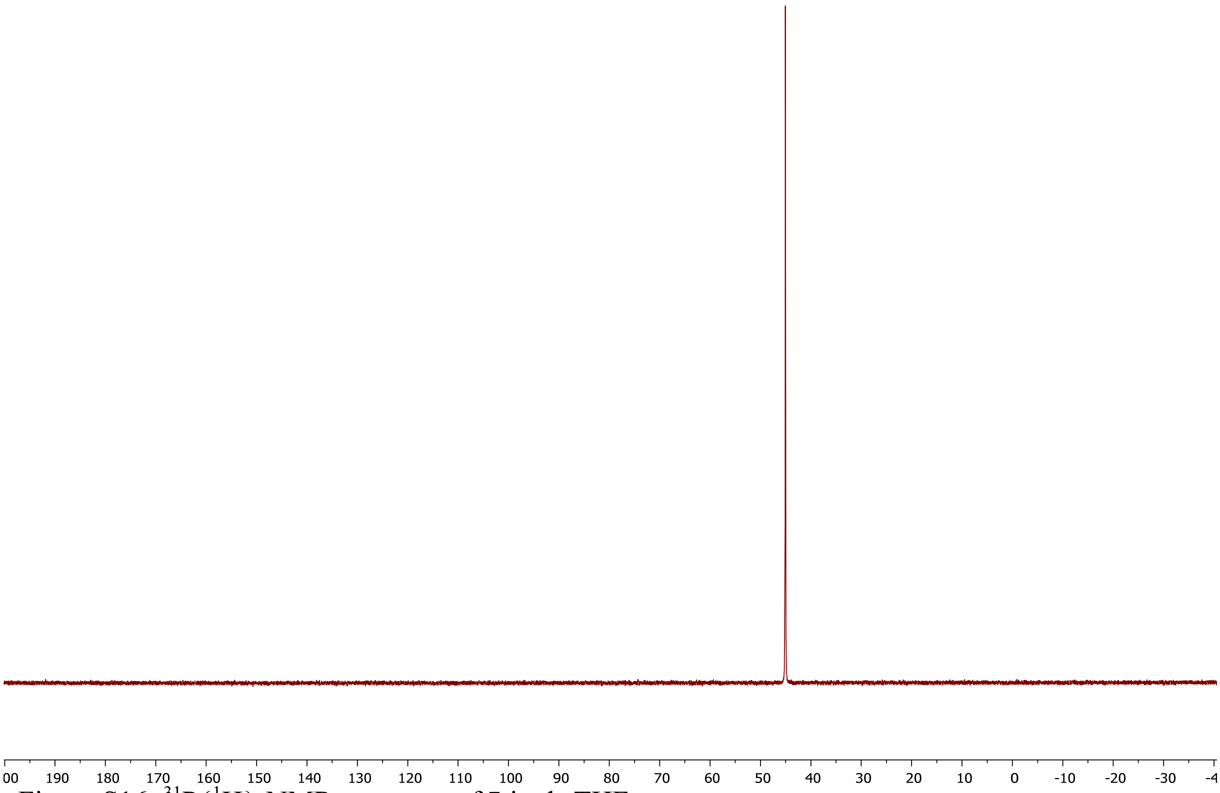

Figure S16: $^{31}P\{^{1}H\}$-NMR spectrum of **5** in d_8-THF

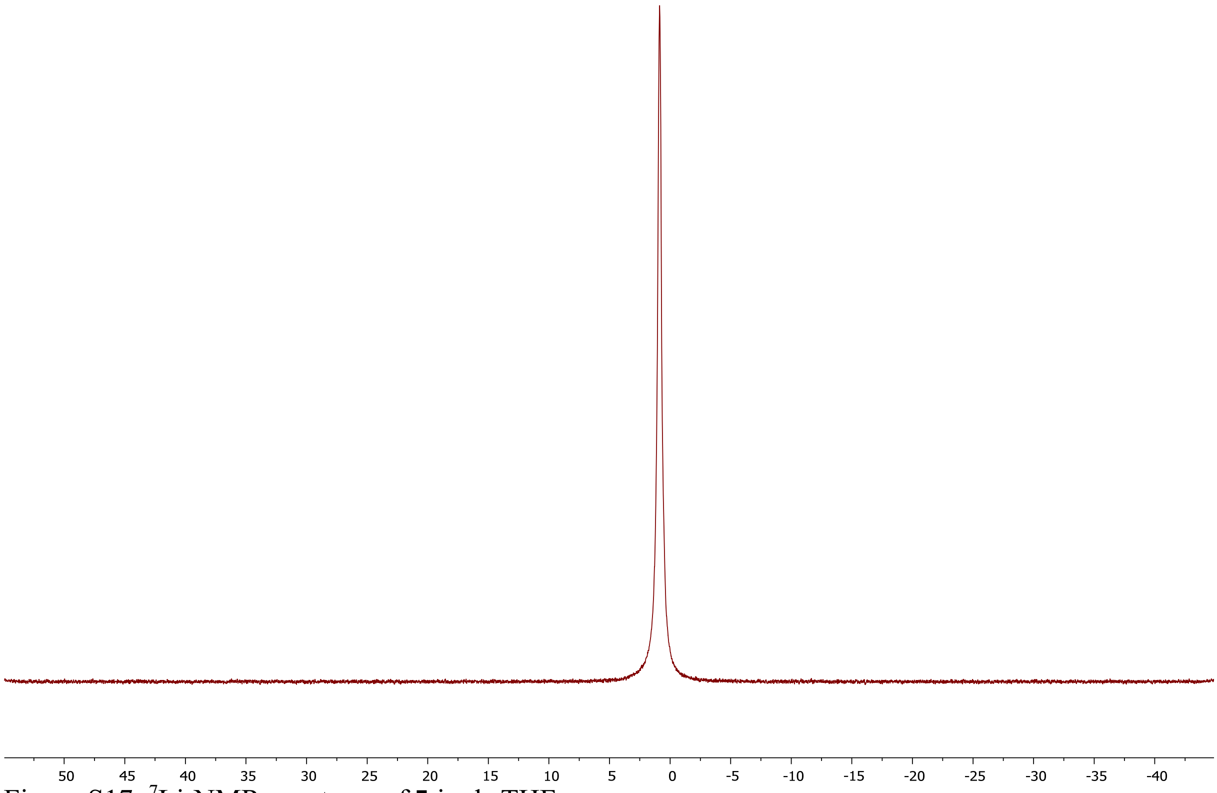

Figure S17: ^{7}Li-NMR spectrum of **5** in d_8-THF

2.2 NMR-Spectra of Compound 6

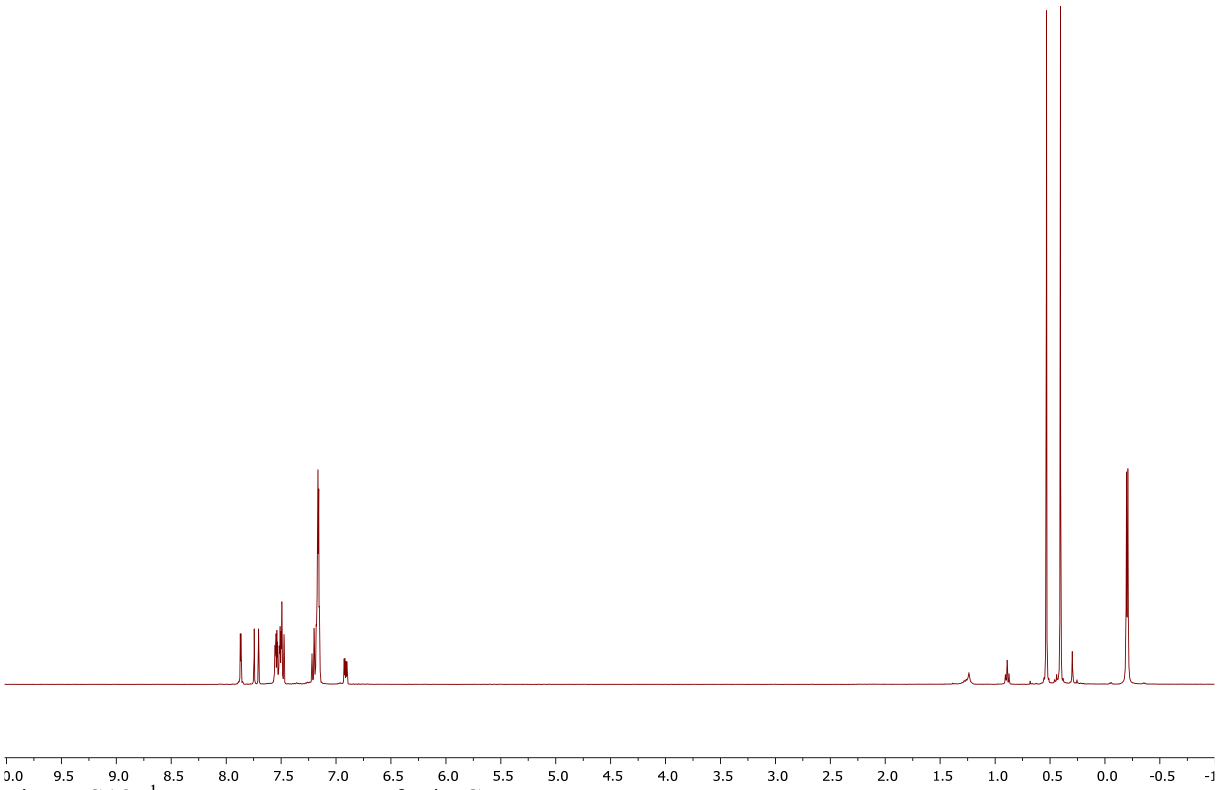

Figure S18: ^{1}H-NMR spectrum of **6** in C_6D_6

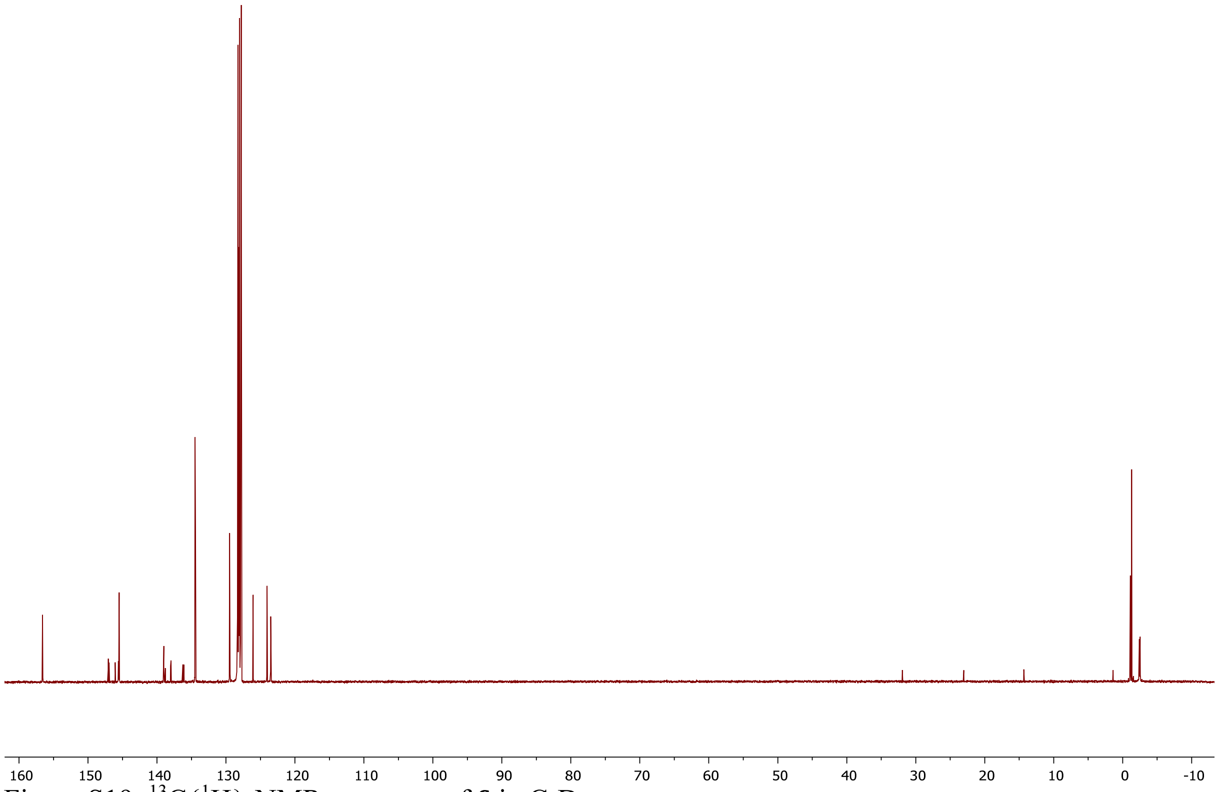

Figure S19: ^{13}C{^{1}H}-NMR spectrum of **6** in C_6D_6

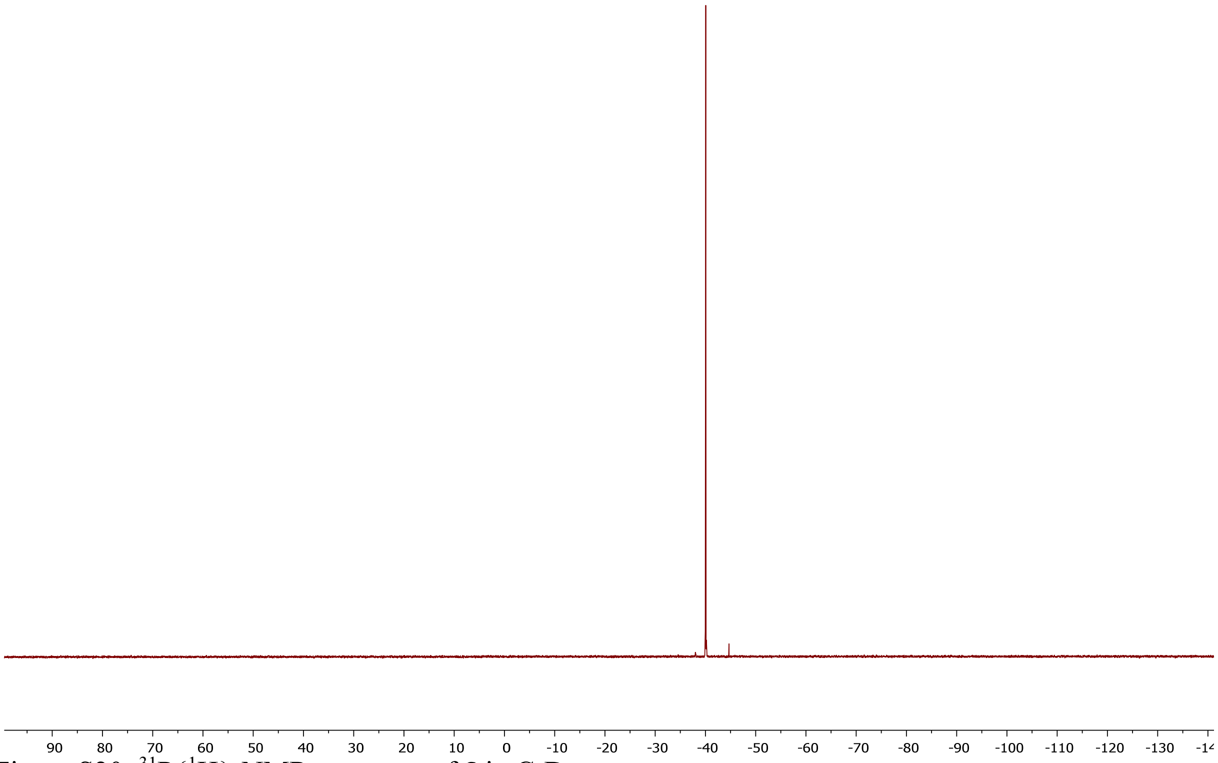

Figure S20: $^{31}P\{^{1}H\}$-NMR spectrum of **6** in C_6D_6

2.3 NMR-Spectra of Compound 7

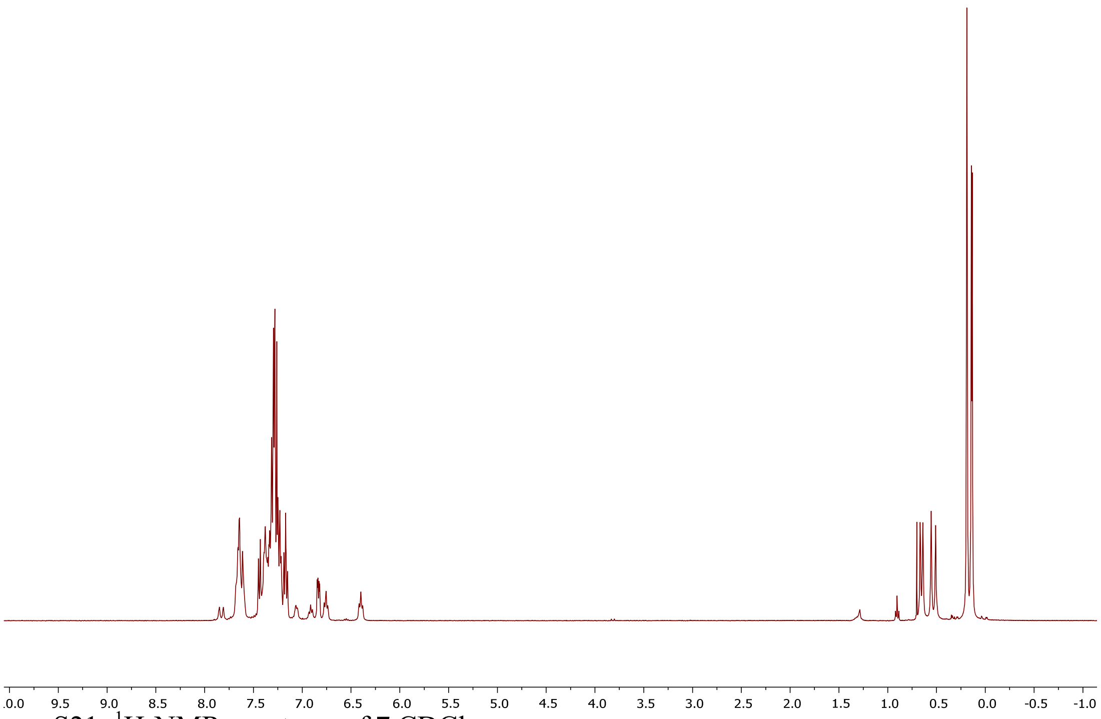

Figure S21: ^{1}H-NMR spectrum of **7** $CDCl_3$

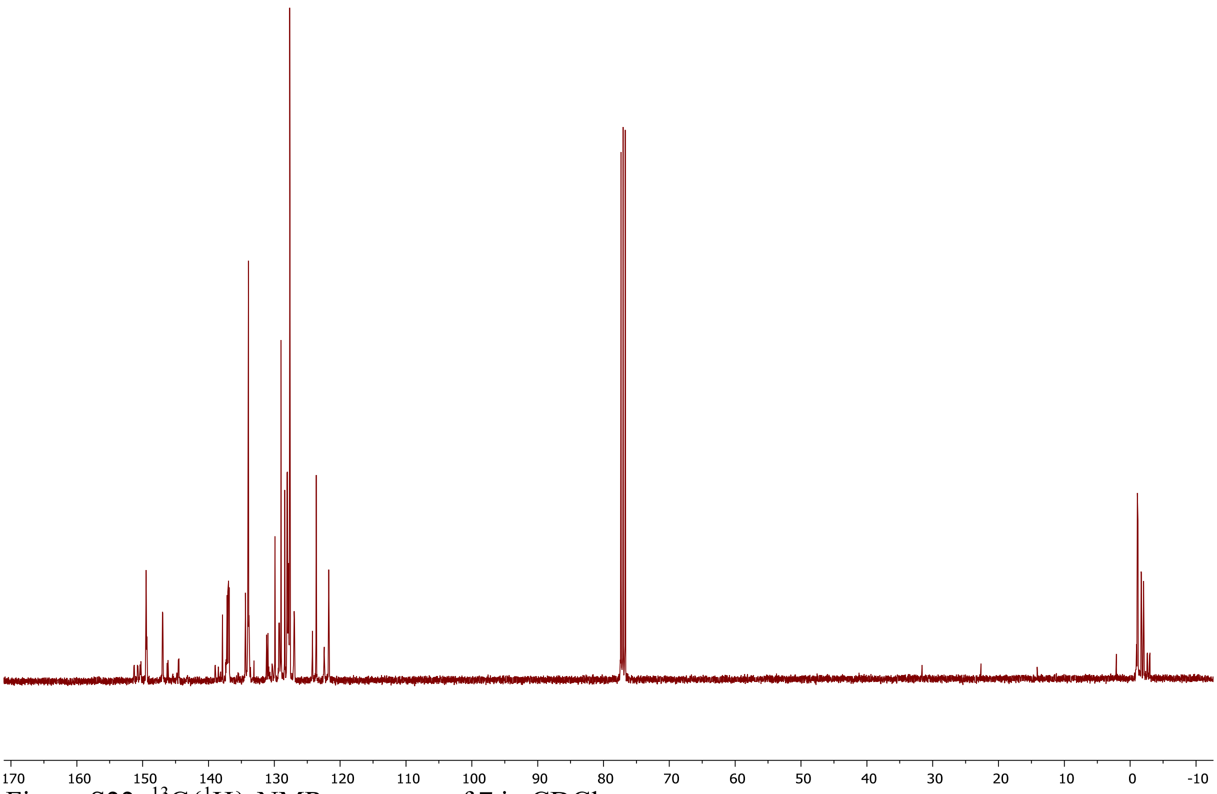

Figure S22: $^{13}C\{^{1}H\}$-NMR spectrum of **7** in $CDCl_3$

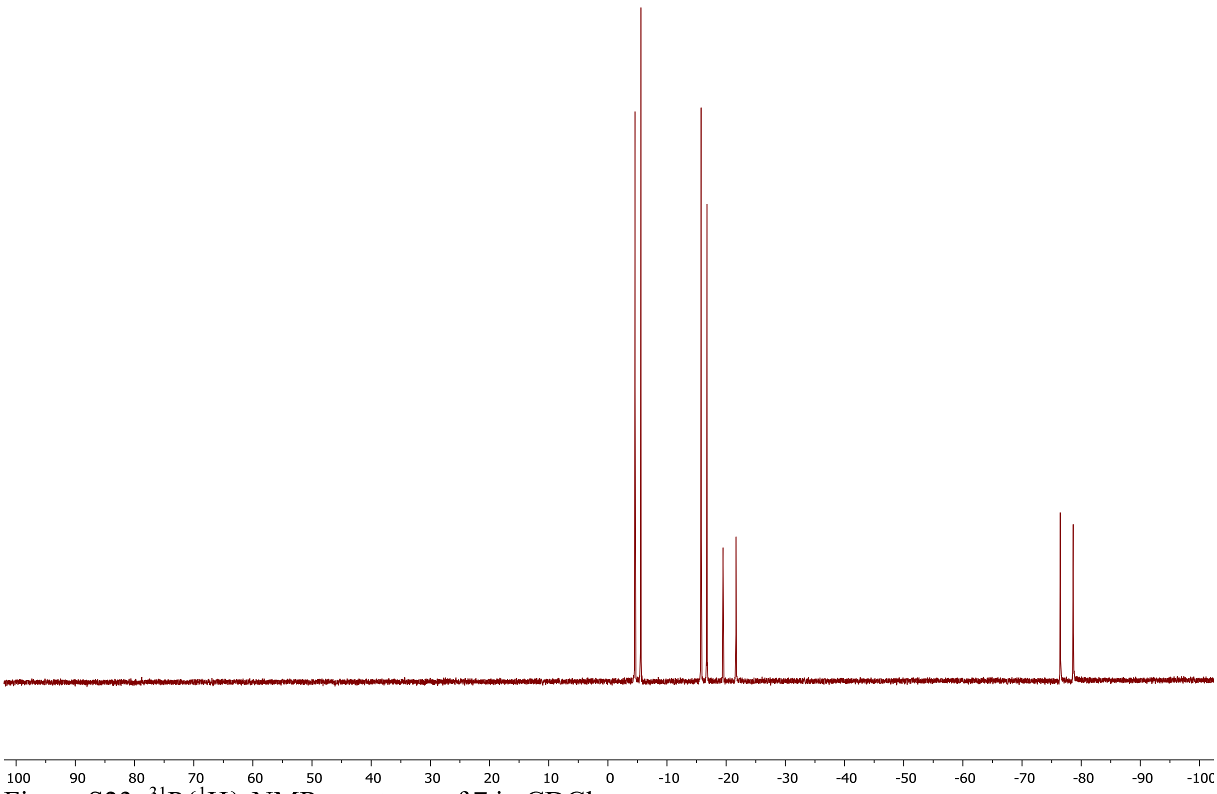

Figure S23: $^{31}P\{^{1}H\}$-NMR spectrum of **7** in $CDCl_3$

2.4 NMR-Spectra of Compound 8

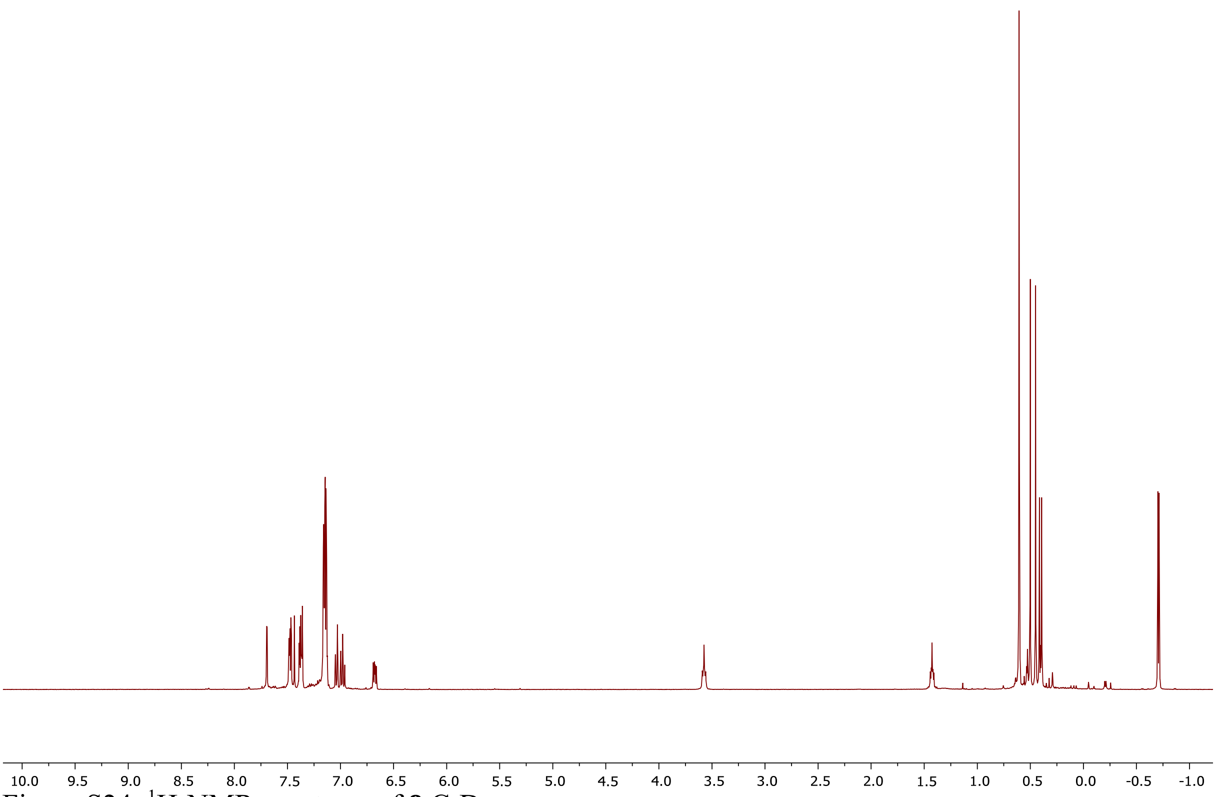

Figure S24: ^{1}H-NMR spectrum of **8** C_6D_6

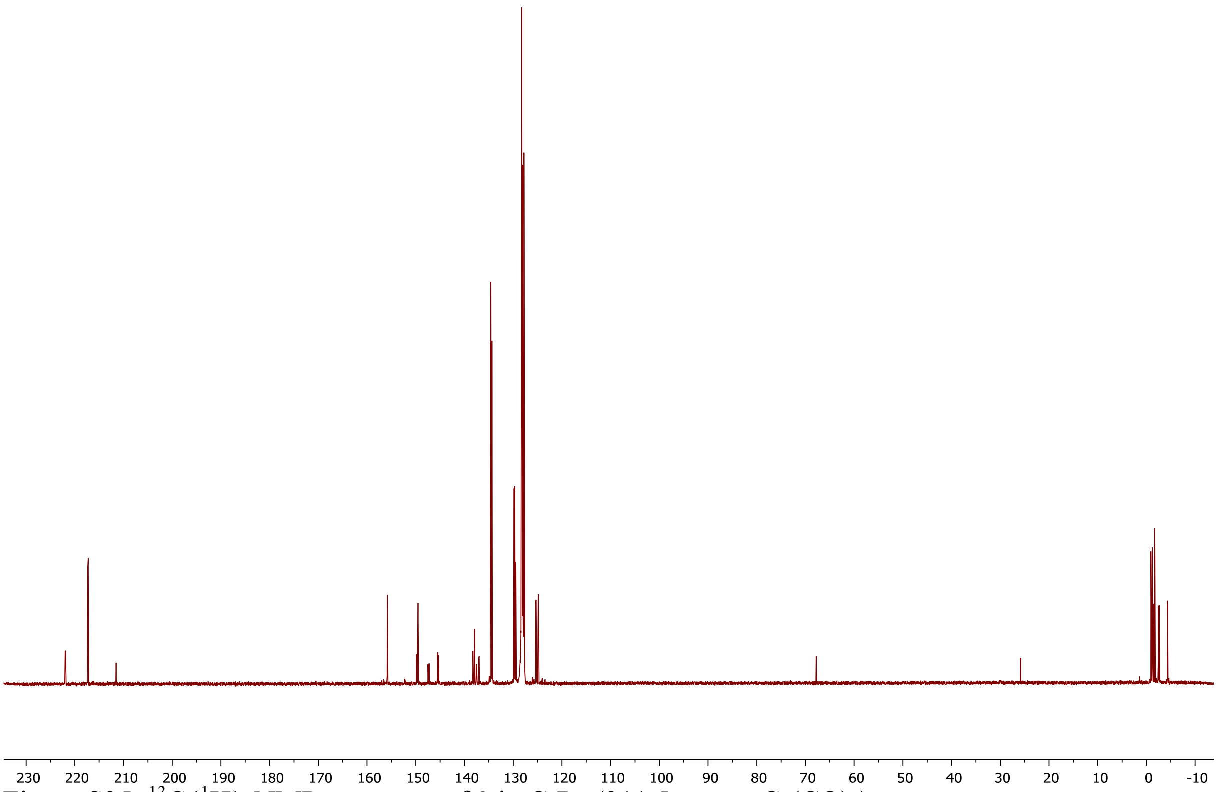

Figure S25: ^{13}C{^{1}H}-NMR spectrum of **8** in C_6D_6 (211.5 ppm = $Cr(CO)_6$)

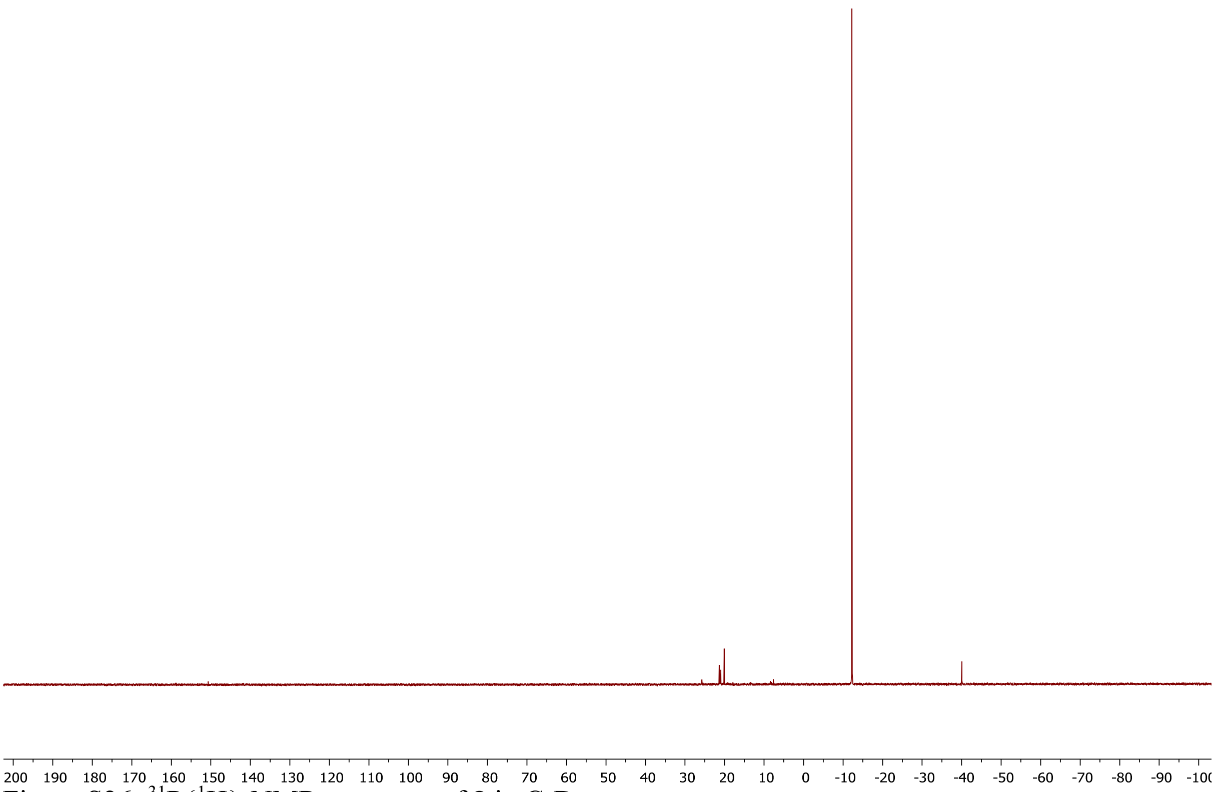

Figure S26: $^{31}P\{^{1}H\}$-NMR spectrum of **8** in C_6D_6

2.5 NMR-Spectra of Compound 9

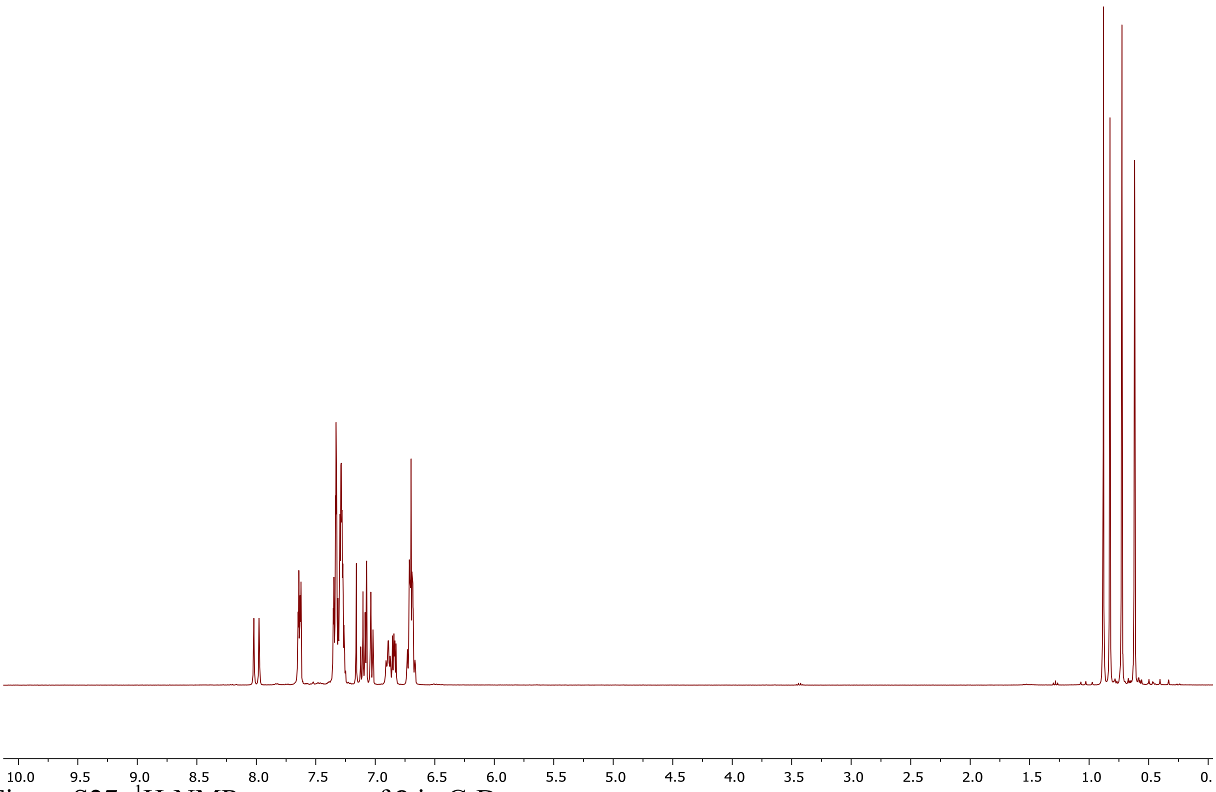

Figure S27: ^{1}H-NMR spectrum of **9** in C_6D_6

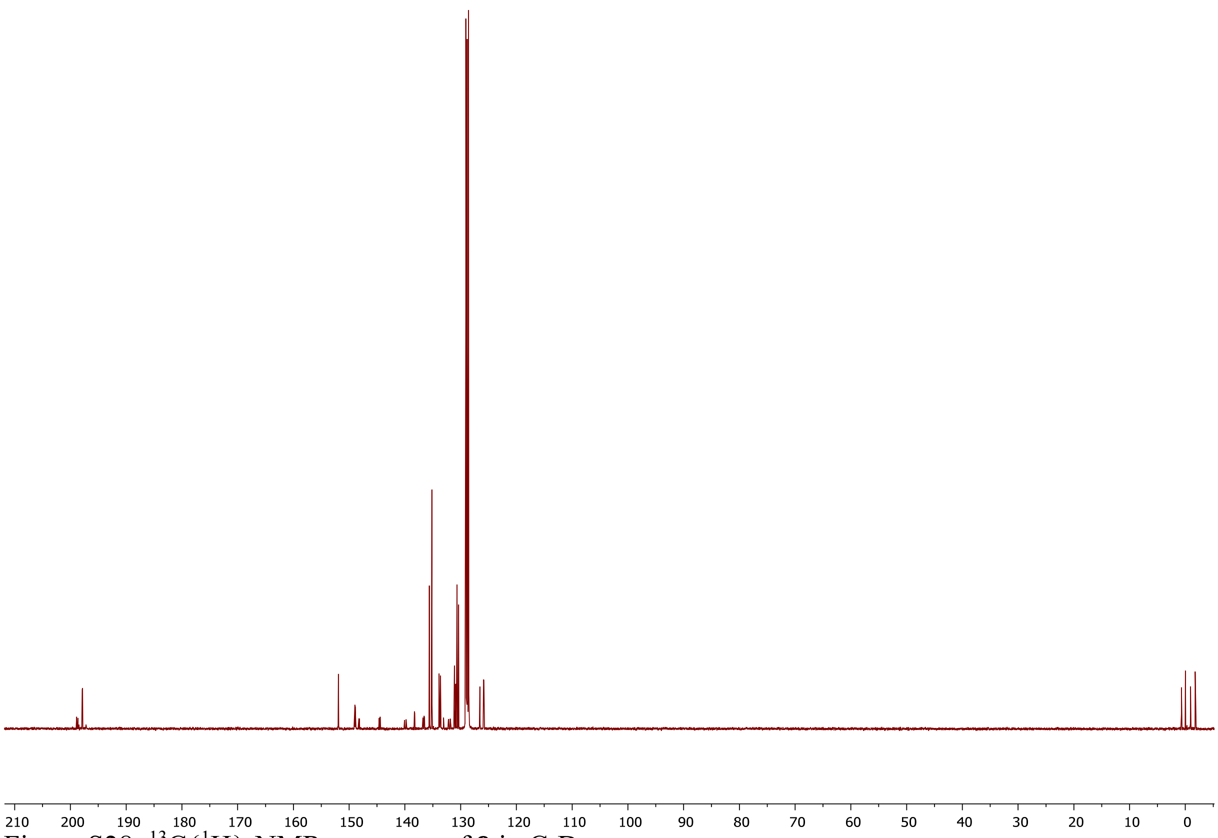

Figure S28: ${}^{13}C\{{}^{1}H\}$-NMR spectrum of **9** in C_6D_6

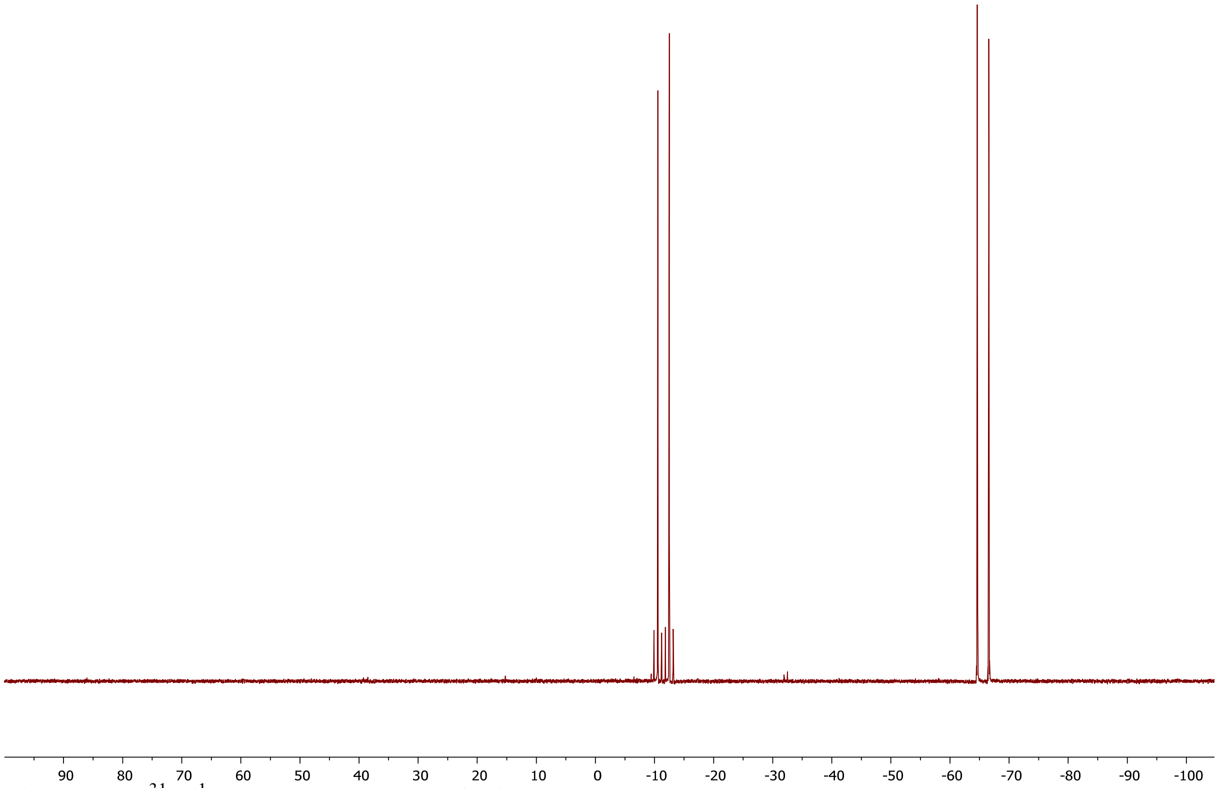

Figure S29: ${}^{31}P\{{}^{1}H\}$-NMR spectrum of **9** in C_6D_6

2.6 NMR-Spectra of Compound 10

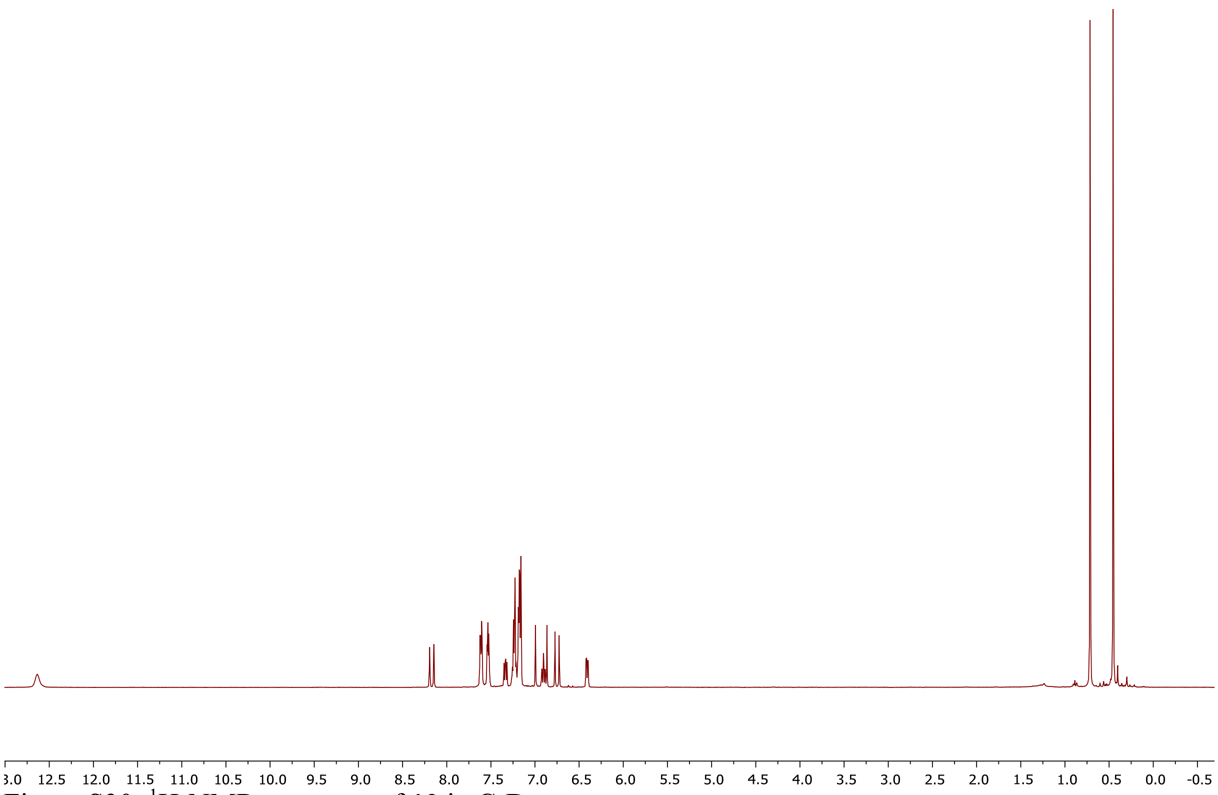

Figure S30: ^{1}H-NMR spectrum of **10** in C_6D_6

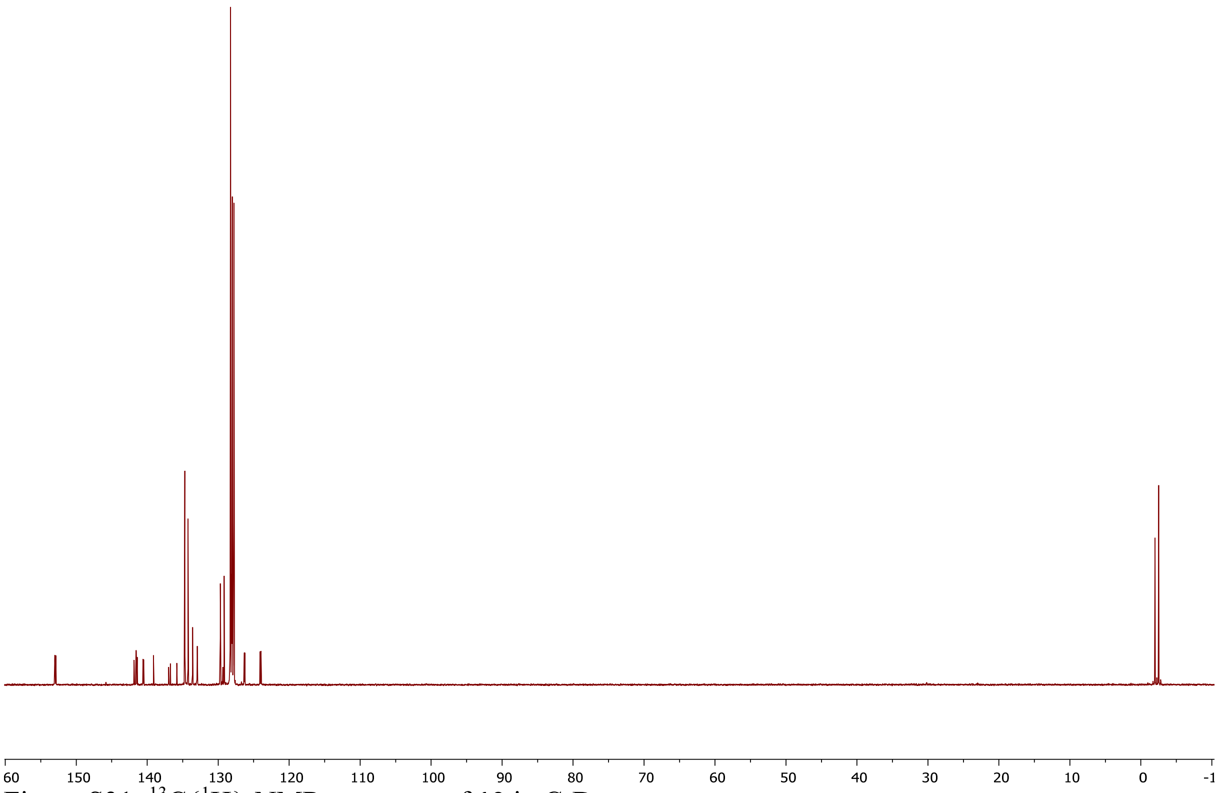

Figure S31: ^{13}C{^{1}H}-NMR spectrum of **10** in C_6D_6

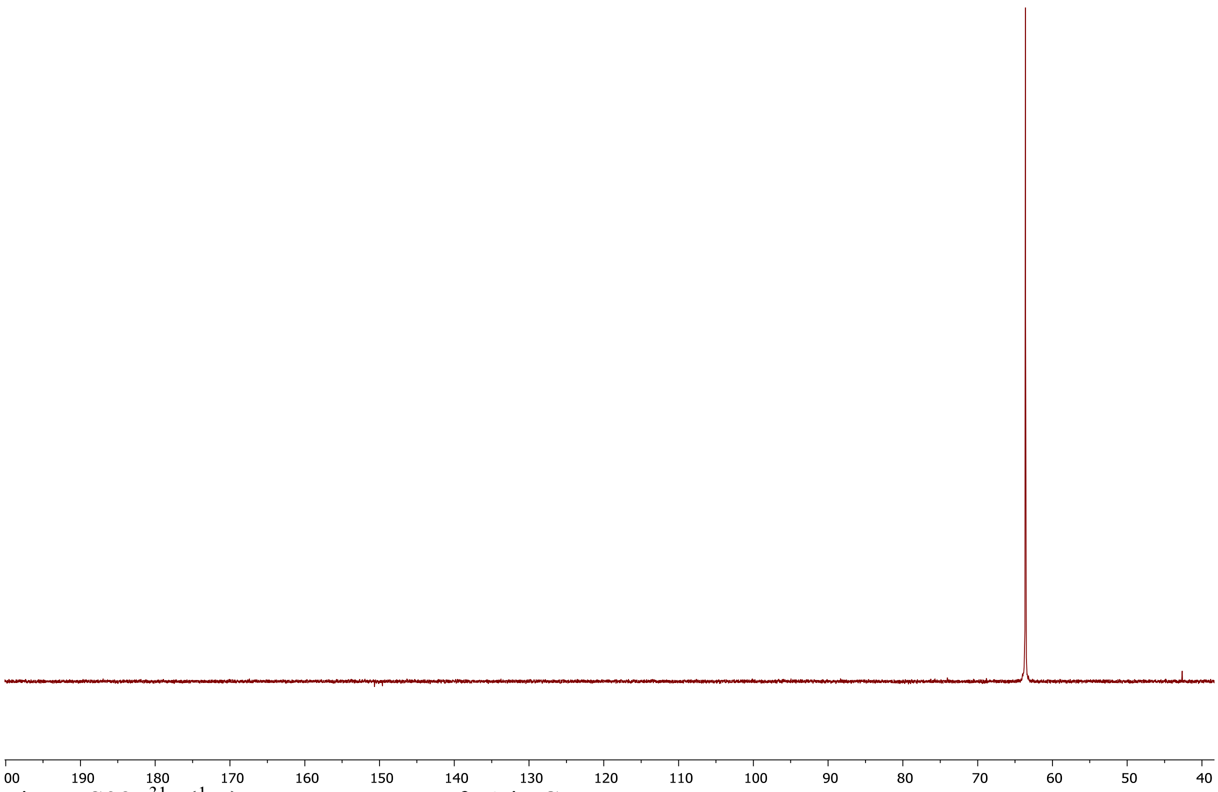

Figure S32: $^{31}P\{^1H\}$-NMR spectrum of **10** in C_6D_6

3 Quantum Chemical Calculations

3.1 Optimized Structure of 5 in Gas Phase

!Grid6 FinalGrid7 PBE0 def2-TZVP d3bj opt freq

Total enthalpy ... -1925.76324239 Eh

Total entropy correction ... -0.09088281 Eh -57.03 kcal/mol

Final Gibbs free enthalpy ... -1925.85412520 Eh

56

Coordinates from ORCA-job Input

P 9.49489112184651 5.82429782374990 10.76196696745999

C 9.99803782219421 7.50521026447127 11.01496484029531

C 11.10071454022009 5.27635868669592 11.34083684672251

C 11.33019708561039 7.59364214719745 11.54580105337329

C 9.28013296531750 8.70562187869723 10.75144437919929

C 11.93420803894332 6.32485563453546 11.71258317206986

Si 11.57527894340661 3.52080059244557 11.30608727659307

C 7.22188161460727 7.63646464655367 9.84157936284825

C 7.93686106548223 8.72150550023325 10.15036631179401

C 11.90196345274427 8.84858536408239 11.83186705534302

C 9.89282080617129 9.91703619485633 11.04465340670175

H 12.94805687295079 6.21183083377820 12.09574749263300

C 10.24569484778037 2.38958007860984 12.01891893477602

C 13.18448206029211 3.17815173889752 12.24455746281446

C 11.85726030959059 2.89316776754610 9.52923493442899

Si 5.63813081600642 7.35358222591309 9.08410544689554

H 7.60362534047708 9.76523071897335 9.96719036219484

H 12.91091084589129 8.89788053735327 12.23823211880419

C 11.18691071676219 9.99808889292983 11.58525898440296

H 9.33941329550575 10.83034756343723 10.83158999591780

H	10.11614901667215	2.58225458816140	13.08776677595352
H	10.48105153956784	1.32999192067866	11.87317934510553
H	9.29575191101555	2.61267597430854	11.52620897845276
H	14.00658812989892	3.75819440000317	11.81682083468862
H	13.46309554460172	2.11977782570615	12.22055822030753
H	13.07059473294656	3.47735866796819	13.29044909072272
C	12.54217703143715	1.70694845614886	9.25564190014157
C	11.33742091847746	3.60237115478615	8.44323791798845
C	4.55248140289981	8.70644708638051	8.23837950193830
C	4.43606619567036	6.55762275869453	10.32446546142365
C	5.90473384020914	6.05535977776603	7.70923388444787
H	11.62327566096245	10.97182107663217	11.79937453011884
H	12.96155256739978	1.12831999138915	10.07540185766010
C	12.70516057934707	1.24049939928088	7.95760115990405
C	11.48924554883234	3.13994875059123	7.14263419961244
H	10.79566944939672	4.52633003696935	8.64047287157433
H	4.33811247866111	9.49908014670319	8.96368949083261
H	3.59093260732460	8.33037958698613	7.86378139827399
H	5.08945658569409	9.16116798947785	7.39982689481427
H	4.93959406381000	5.73314826529736	10.83440726480745
H	3.53544840536884	6.17403525613616	9.83190182072900
H	4.13221576471298	7.28900445204866	11.08087572203000
C	7.06735688794342	5.27574372136411	7.74013084455514
C	4.99612559080160	5.81250357593911	6.67600852460614
H	13.24391191334314	0.31466573763147	7.77461106969255
C	12.17453004909662	1.95838478834490	6.89429742265137
H	11.06606040843438	3.70804980587180	6.31990518296556
H	7.78165336159736	5.45797020538349	8.54225293673938
C	7.30682272377130	4.30168155351086	6.77953332704264
C	5.22723457975963	4.83847304948150	5.71142109208389
H	4.08431908010419	6.40214124276500	6.61699246509082

H	12.29349299124239	1.59690877928165	5.87706434875031
H	8.21711081342762	3.71105773098991	6.83204689210212
C	6.38735416372100	4.07688801859260	5.76182709352960
H	4.50209898701722	4.67341818802724	4.91796444586533
H	6.57402191303320	3.31304694974515	5.01166882752938

3.2 Structure of 5 with Dummy Atom for NICS(0) Calculation

P	6.02801	4.24972	8.85059
C	6.53116	5.93063	9.10358
C	7.63383	3.70178	9.42946
C	7.86332	6.01906	9.63442
C	5.81325	7.13104	8.84006
C	8.46733	4.75028	9.80120
Si	8.10840	1.94622	9.39471
C	3.75500	6.06188	7.93020
C	4.46998	7.14693	8.23899
C	8.43508	7.27401	9.92049
C	6.42594	8.34246	9.13327
H	9.48118	4.63725	10.18437
C	6.77881	0.81500	10.10754
C	9.71760	1.60357	10.33318
C	8.39038	1.31859	7.61785
Si	2.17125	5.77900	7.17273
H	4.13675	8.19065	8.05581
H	9.44403	7.32330	10.32685
C	7.72003	8.42351	9.67388
H	5.87253	9.25577	8.92021
H	6.64927	1.00767	11.17639
H	7.01417	-0.24459	9.96180
H	5.82887	1.03810	9.61483

H	10.53971	2.18361	9.90544
H	9.99622	0.54520	10.30918
H	9.60371	1.90278	11.37907
C	9.07530	0.13237	7.34426
C	7.87054	2.02779	6.53186
C	1.08560	7.13187	6.32700
C	0.96919	4.98304	8.41309
C	2.43785	4.48078	5.79785
H	8.15640	9.39724	9.88799
H	9.49467	-0.44626	8.16402
C	9.23828	-0.33408	6.04622
C	8.02237	1.56537	5.23125
H	7.32879	2.95175	6.72909
H	0.87123	7.92450	7.05231
H	0.12405	6.75580	5.95240
H	1.62258	7.58659	5.48845
H	1.47271	4.15857	8.92303
H	0.06857	4.59946	7.92052
H	0.66534	5.71442	9.16950
C	3.60048	3.70116	5.82875
C	1.52925	4.23792	4.76463
H	9.77703	-1.25991	5.86323
C	8.70765	0.38380	4.98292
H	7.59918	2.13347	4.40853
H	4.31477	3.88339	6.63087
C	3.83994	2.72710	4.86815
C	1.76035	3.26389	3.80004
H	0.61744	4.82756	4.70561
H	8.82661	0.02233	3.96568
H	4.75023	2.13648	4.92067
C	2.92047	2.50231	3.85045

H	1.03522	3.09884	3.00658
H	3.10714	1.73847	3.10029
DA	7.24630	4.90410	9.34036

3.3 Optimized Structure of 7 (S,R) with Solvent-Correction

!Grid7 NoFinalgrid PBE0 def2-TZVP d3bj CPCM(Chloroform) tightopt verytightscf numfreq normalprint

Total enthalpy ... -2498.47281911 Eh

Total entropy correction ... -0.10154194 Eh -63.72 kcal/mol

Final Gibbs free enthalpy ... -2498.57436105 Eh

68

Coordinates from ORCA-job Input

P	0.53707710391846	7.24836368361299	5.64516980009804
P	1.92977539520189	8.92634757761354	5.85820173471791
C	1.84166512864063	6.22165832192701	4.97216186095459
C	0.47687267357810	6.22790528435134	7.13619664498095
C	2.31409843171028	9.00298424648067	4.05766689083700
C	1.97803033246095	5.04181059219980	5.71061511966885
C	2.66061808575330	6.53293728292888	3.89407625601186
Si	-0.56969380868668	6.65739013262125	8.61980654147862
C	1.17600913121717	5.08049732565286	6.92117398098502
C	2.65340450680753	7.89543324744385	3.36130044524659
Si	2.62596795410298	10.69000716449204	3.25952304120720
C	2.87233790533232	4.06671470079678	5.25938303609998
C	3.54834335552597	5.54995115294734	3.46008882727105
C	-0.72287788281576	5.12644273582571	9.69157961469667
C	-2.25149161607122	7.22446559303237	8.02947506029772
C	0.27014718236262	8.04037975604470	9.55624891485197
H	1.15849498005189	4.24307380564818	7.61380383860227

H	3.04454319645748	8.01314865834126	2.35299875907686
C	3.58113639342012	10.37636025110172	1.67192449043012
C	3.68244442276745	11.71513120639564	4.41454642660357
C	1.05010829573672	11.60220147631699	2.83809741826955
H	2.98941580690282	3.13227163700990	5.79714005496530
C	3.62881908130856	4.32516327607014	4.12556835047634
H	4.20510531273298	5.75227345263850	2.62045174451081
C	-0.14188176795077	5.05009418141122	10.95874357760158
C	-1.42011100726446	4.00820268429402	9.22358685179313
H	-2.87634276677124	7.51452164611354	8.87827525090939
H	-2.76537118251036	6.43483185845817	7.47607203215137
H	-2.15070128367429	8.08999429012424	7.36793466970377
H	-0.30252986677472	8.31540055547111	10.44613721370775
H	0.34706590451694	8.92574717652790	8.91865492970958
H	1.28003690359587	7.75993637096716	9.86454544160784
C	4.95184012786412	10.10369252577521	1.70483855395743
C	2.94840981820350	10.38382066333538	0.42711127171185
H	3.14016915801873	11.96342504948832	5.32910312039567
H	3.96199917647725	12.65018286410711	3.92128932286567
H	4.59745275335699	11.18808152582905	4.69477561580686
H	0.55502717285852	11.96436534147471	3.73983691763861
H	0.34601739028791	10.96511870260951	2.29784905440275
H	1.29009772829232	12.46293361369229	2.20723763137285
H	4.32664799737072	3.57622018692879	3.76850320189999
H	0.40754099671489	5.89896440971862	11.35294725312894
C	-0.25211797819587	3.90193175046339	11.73301765977782
H	-1.88108526246934	4.03196287492852	8.24025524725301
C	-1.53414305208891	2.85815920091577	9.99060794571195
H	5.47604356414221	10.08466109879281	2.65551425655877
C	5.66431441355527	9.84996036379618	0.54191325656531
H	1.88497279374630	10.59115482453376	0.36324386693958

C 3.65411310676534 10.12898047876270 -0.74182433015561

H 0.20687231024247 3.86469975000677 12.71495127645886

C -0.94888209203165 2.80373191800992 11.24947633587664

H -2.07956962917759 2.00251894245199 9.60771140409111

C 5.01461585098892 9.86157180671750 -0.68584881026159

H 6.72775120785735 9.64293598569877 0.59175493195552

H 3.14136652617238 10.13984653171909 -1.69744086296490

H -1.03630164147507 1.90611394346102 11.85157942447958

H 5.56874857607415 9.66351281976208 -1.59666641250433

C 0.74165652865279 10.29036580136831 6.15711855064408

C 1.11596806488714 11.26297801148730 7.08257036070015

C -0.52380636239320 10.36452309595594 5.57558334308339

C 0.25171260511191 12.29969333192564 7.40932608371631

H 2.09149450831707 11.20381511876416 7.55445392969034

C -1.00239237797386 12.36582393529859 6.82105282051030

H 0.55741468641940 13.05036379167333 8.12914876346967

C -1.38955101098136 11.39427928219346 5.90530884563435

H -0.83280979603401 9.61119003589755 4.85951228765844

H -2.36994341066610 11.44136284734895 5.44474113326263

H -1.68190575047422 13.17036025024670 7.07847192914473

3.4 Optimized Structure of 7 (*S*,*S*) with Solvent-Correction

!Grid5 FinalGrid6 PBE0 def2-TZVP d3bj CPCM(Chloroform) tightopt numfreq normalprint

Total enthalpy ... -2498.47645196 Eh

Total entropy correction ... -0.10043130 Eh -63.02 kcal/mol

Final Gibbs free enthalpy ... -2498.57688326 Eh

68

Coordinates from ORCA-job Input

P	0.15013060884002	7.08422699775960	5.68009265306768
P	1.73201554625871	8.40733112103678	6.47223010469050
C	1.22186148375471	6.16930284430282	4.59187103670539
C	-0.16270681077340	5.59806859753781	6.66732080862156
C	2.32537699714715	8.92170239199035	4.80132623984722
C	1.14358878765612	4.79422434805576	4.83649176135885
C	2.10439642771597	6.71043924987689	3.66578665242080
Si	-1.27605810139954	5.52971397459531	8.16504838821932
C	0.33444542111167	4.51570322696540	6.00904294660553
C	2.40134250698535	8.13780900949395	3.70529235567168
Si	2.93386909442583	10.69976271372889	4.77611398005635
C	1.86033818965634	3.92141316597909	4.01697474461520
C	2.80558769932432	5.82409709605867	2.84772541192963
C	-0.46767069104528	6.53855089074499	9.52676819002356
C	-1.47276866402405	3.75324619262517	8.70526627317646
C	-2.92659545541685	6.29323608471953	7.73024877504586
H	0.14599276750704	3.49445590268390	6.32934944588689
H	2.82599602906619	8.56269097019508	2.79627098860902
C	3.80778634878501	10.99960805665356	3.14304761875223
C	4.13891806079057	10.91430630745289	6.19237238737449
C	1.48351384304984	11.86132858038968	4.97210768046261
H	1.80499034055544	2.84889646171574	4.16916586500777
C	2.66310157073667	4.44736313353091	3.01279157421742
H	3.49942655183855	6.21124146884599	2.10863375888843
C	-0.47265061024915	7.93509250861629	9.47179046680399
C	0.22337447935531	5.92966787224467	10.57680724167163
H	-2.08167896010779	3.69863120770336	9.61154307312172
H	-0.50940887387648	3.28108042902427	8.91245706447914
H	-1.97353882551468	3.17354135933756	7.92559293515765
H	-3.59171640183027	6.28887599358544	8.59788578247195

H	-3.40365424705478	5.73152145148837	6.92295781550884
H	-2.80966386768611	7.32680220885511	7.39465751431316
C	5.05601949270445	10.42002432955619	2.89467953256361
C	3.23417922908042	11.77079062651455	2.13022785629843
H	3.66119643689496	10.68569672589382	7.14891939871728
H	4.49414765743265	11.94764250549840	6.22833972907215
H	5.00448496721227	10.25677437968812	6.08252052553368
H	0.97533398128939	11.65952231740372	5.91870020477006
H	0.75785168945686	11.73174912442401	4.16564500071522
H	1.81571539762795	12.90297636428621	4.97733432938101
H	3.22125637948890	3.77673074622435	2.36945746070231
H	-0.99646195476455	8.44565307793997	8.66934354670815
C	0.19686055549498	8.69589729096566	10.41970559200103
H	0.25242530214047	4.84724024375531	10.64885747634072
C	0.89171680606879	6.68378889476310	11.53178139883204
H	5.53071852193236	9.81078699437159	3.65840775270855
C	5.70725881703160	10.60268028979040	1.68371409990773
H	2.26595215806595	12.23501883070401	2.28774040181215
C	3.88046053868955	11.95941009377280	0.91516275801369
H	0.18880313163877	9.77788800650073	10.34948127635370
C	0.88312605490354	8.06989778269686	11.45178556273772
H	1.42562218247269	6.18965506588324	12.33614933142342
C	5.11860390081450	11.37472927921784	0.69004467855348
H	6.67496273171607	10.14362719269747	1.51345683147065
H	3.41651093851302	12.56328642947529	0.14297891681402
H	1.41102047942101	8.66124091566193	12.19157524136766
H	5.62516174952806	11.51988126224319	-0.25767756906790
C	3.05825677541393	7.29108034947845	7.05091144599662
C	2.93704741893930	6.81454449476320	8.35792006065144
C	4.16922078231762	6.91890102751932	6.29690170531827
C	3.89298407668285	5.96487954007560	8.88995700752043

H 2.08559899498371 7.10856039892438 8.96044655796168

C 4.99600249814439 5.59524786951299 8.13054503055063

H 3.77860682434691 5.59742034977956 9.90382129122767

C 5.13302241587252 6.07903606147858 6.83683132209207

H 4.28357804572722 7.28606673143544 5.28537710500309

H 5.99360560601824 5.79817833893279 6.23969843640979

H 5.74905817111613 4.93624425037693 8.54785716875520

3.5 Optimized Structure of Phosphoric Acid as Reference for NMR Calculations

!Grid5 FinalGrid6 PBE0 def2-TZVP CPCM(Chloroform) d3bj tightopt verytightscf numfreq

Total enthalpy ... -643.86969595 Eh

Total entropy correction ... -0.03619163 Eh -22.71 kcal/mol

Final Gibbs free energy ... -643.90588758 Eh

8

Coordinates from ORCA-job Input

P -4.69063350796895 0.49877921247310 0.11272864873468

O -3.30888648012770 0.41668861383740 0.86578249507568

O -4.61732288364226 -0.74848122551954 -0.85217256151414

O -4.60004488459736 1.70920714709684 -0.90891543168871

H -4.90711125267117 2.54032512028118 -0.52524640184670

H -2.53282255636921 0.44290985413306 0.29234482338187

H -5.42766505354080 -0.89620081784504 -1.35588247685023

O -5.81269338108252 0.56370209554300 1.04942090470754

3.6 Optimized Structure of 7 (S,R) in Gas Phase

!Grid6 FinalGrid7 PBE0 def2-TZVP d3bj tightopt numfreq

Total enthalpy ... -2498.45825960 Eh

Total entropy correction ... -0.10119126 Eh -63.50 kcal/mol

Final Gibbs free enthalpy ... -2498.55945086 Eh

68

Coordinates from ORCA-job Input

P 0.53132088138187 7.25974055893180 5.67010675381841

P 1.93395398970768 8.93742209269745 5.83826615463572

C 1.80963474156187 6.22611223001559 4.95989989541249

C 0.51741819246797 6.24438664708376 7.16473026066693

C 2.26140421582495 9.00328399869579 4.02621448502185

C 1.96345440988276 5.04679794392264 5.69482106480510

C 2.59535860020165 6.53196969949875 3.85652768926448

Si -0.46275600411243 6.68472998500968 8.68585816217497

C 1.20152034823286 5.09224276318846 6.92956808110860

C 2.57812911050382 7.89332116623601 3.32346289444374

Si 2.54809543989602 10.67957847042075 3.20454930931670

C 2.83776428284686 4.06791337081739 5.21558152754466

C 3.46414728506725 5.54522934816092 3.39670576758541

C -0.68448147868334 5.13307244624478 9.71437529178814

C -2.11971347024867 7.37822107646926 8.16172510639995

C 0.47968275426487 7.98419290878611 9.64677947294237

H 1.19823956402641 4.25757558745610 7.62590395025779

H 2.94380011013853 8.00942497974062 2.30528274512845

C 3.58589359436606 10.36801137768648 1.66942193279189

C 3.51099615488820 11.78226811574966 4.37152626050869

C	0.95846282398782	11.52132985593474	2.69030527139332
H	2.96705538341959	3.13310778499050	5.74975163083734
C	3.55951065180396	4.32131731144705	4.05968918539134
H	4.09605490170596	5.74583804917026	2.53793916204696
C	-0.15644578244904	5.01326939847968	11.00005136510730
C	-1.38749370858400	4.04287834648747	9.19398500587370
H	-2.71089386262993	7.66589807540749	9.03472557947175
H	-2.69351691343815	6.64823182259544	7.58640413379630
H	-1.98195860750815	8.26514865045565	7.53641910648656
H	-0.04939595829194	8.26683661165323	10.56082688824515
H	0.59282742494665	8.88400887760396	9.03592115312324
H	1.47982466385570	7.63715222163764	9.91567464950722
C	4.94090109616329	10.04606283699583	1.78481627763453
C	3.03943473879728	10.42624565207288	0.38703261293684
H	2.90574503230593	12.06957184155642	5.23329325052091
H	3.81400957179183	12.69419680524569	3.85042984483421
H	4.41183439304726	11.28742099595788	4.74136819532248
H	0.40799991137368	11.88089672688720	3.56035184455385
H	0.30435061250042	10.84566683666913	2.13435091779724
H	1.18583726022793	12.38027001122576	2.05269568741416
H	4.24222254752541	3.56871301615809	3.68213626021096
H	0.39756541397077	5.83947338633119	11.43368665447268
C	-0.32383996729980	3.85185552270370	11.74173618851857
H	-1.80478691789521	4.10033360463098	8.19276842285996
C	-1.55864664895857	2.87993076071926	9.92805096263610
H	5.39749728899904	9.98428287659670	2.76802571090214
C	5.71963044624214	9.79408226337315	0.66656482454942
H	1.99041172462265	10.67255304399322	0.25914902267531
C	3.81200606878024	10.17324541616748	-0.73836938403730
H	0.09555116423910	3.78093303321283	12.73920811442775
C	-1.02589872628408	2.78315800534780	11.20633047420829

H -2.10796196505574 2.04660816654455 9.50415226495824

C 5.15442477410030 9.85680561654138 -0.59989880417626

H 6.76950884857173 9.54811396214583 0.78109224900289

H 3.36471730787168 10.22350782857402 -1.72489392125908

H -1.15785702710663 1.87466782072136 11.78316185439493

H 5.76075738984208 9.65946727475026 -1.47675050801158

C 0.72899150546548 10.28870884679003 6.14602467449295

C 1.07068266306451 11.23160864573178 7.11316371086657

C -0.52156256725931 10.37642675414785 5.53543328443619

C 0.19063540563004 12.24918382302937 7.45512068621044

H 2.03626670385838 11.16062431130531 7.60357523549342

C -1.04663781017541 12.32954409045836 6.83607660915961

H 0.47115405215161 12.97497174852024 8.20974607948357

C -1.40169001398888 11.38964872699120 5.87654752814724

H -0.80653564175919 9.63971257041645 4.79258679272746

H -2.37020834154043 11.44694983422607 5.39263895656390

H -1.73838403285323 13.12005557058556 7.10352748817486

3.7 Optimized Structure of 7 (S,S) in Gas Phase

!Grid4 FinalGrid5 PBE0 def2-TZVP d3bj tightopt freq

Total enthalpy ... -2498.46215026 Eh

Total entropy correction ... -0.10085220 Eh -63.29 kcal/mol

Final Gibbs free enthalpy ... -2498.56300246 Eh

68

Coordinates from ORCA-job Input

P 0.09812794081455 7.07478996063122 5.67778509408559

P 1.68173746625078 8.40556528683055 6.46010671719353

C	1.14116942239127	6.19259606160349	4.53790073315097
C	-0.15071257532312	5.56784241904074	6.65185130667388
C	2.27703030830874	8.92770925968421	4.79443609308609
C	1.08505170259539	4.81180628292060	4.75543436526175
C	1.98742358934932	6.75971136000764	3.59338283818490
Si	-1.19858158365541	5.45775402483451	8.18924147352501
C	0.33147122963247	4.50328528624935	5.95539719155018
C	2.30729709510981	8.17957024960884	3.67151637560608
Si	2.93960289979109	10.68462346855366	4.80683044326200
C	1.77232333197879	3.96346671751783	3.88750850234754
C	2.65755421176429	5.89648604189909	2.72691355285799
C	-0.43471081977751	6.55391417280247	9.50684938962229
C	-1.24218781614435	3.68293413960329	8.77684674742685
C	-2.92089730177930	6.07729654179629	7.80235196688518
H	0.17211403897698	3.47479120308589	6.26842702343239
H	2.72716456793246	8.62431954952930	2.76965082524402
C	3.85688462505629	10.97871427138635	3.19688129200768
C	4.11837321132402	10.85182179504830	6.25145406454193
C	1.52137110065957	11.88954994944901	4.98928407389043
H	1.73104318622946	2.88757702427299	4.01806617768384
C	2.52783905091938	4.51618200166792	2.86301343296706
H	3.32228385255066	6.30611412055657	1.97329763838663
C	-0.49484195775126	7.94453963812131	9.38838567584310
C	0.26020239965769	6.02281529962493	10.59456043379233
H	-1.80000436718989	3.60038254771515	9.71304604460658
H	-0.23783855206942	3.28633658609298	8.94349068222248
H	-1.73662527793845	3.04686926650533	8.03846784120861
H	-3.55231092987249	6.05720499510555	8.69414403639492
H	-3.38669231007936	5.45757626328193	7.03223016802167
H	-2.89500484399369	7.10339737104907	7.42774463795976
C	5.07559589521599	10.33781775107763	2.95606422612070

C	3.34732710698831	11.80403574913718	2.19400973840591
H	3.60865163383898	10.64464565006586	7.19588186483323
H	4.51940282727081	11.86754759787543	6.29678105251150
H	4.95687476397959	10.15703773340756	6.16804453048950
H	0.99308005361784	11.68976188708200	5.92492805072447
H	0.80160111554608	11.78836370074700	4.17384047384370
H	1.87705132530572	12.92291393082161	5.01284793659971
H	3.06142017710670	3.86420221799181	2.18111370281904
H	-1.02149854432026	8.39625372754106	8.55328533814895
C	0.12477409426072	8.77379041009820	10.31094221333869
H	0.33120223453411	4.94695074601317	10.71594104026893
C	0.87703166898948	6.84570396067974	11.52590165993376
H	5.49929431237498	9.68505381577725	3.71378853738239
C	5.75871882701788	10.51335464955875	1.76304115336255
H	2.40352670947228	12.31721461492675	2.34687878576193
C	4.02583882034346	11.98591332874553	0.99669756526319
H	0.07764609481112	9.84968341566334	10.18795660609783
C	0.81376257460156	8.22430604175105	11.38267800452907
H	1.41289796279764	6.41117482701350	12.36258086982840
C	5.23302243399049	11.33989698767508	0.77933428341020
H	6.70348940603868	10.00698921339785	1.59909394834450
H	3.61097804990687	12.63306344492275	0.23193852815628
H	1.30256337439128	8.86962836631525	12.10362619332144
H	5.76505737169002	11.48003540273248	-0.15486166498398
C	3.01515241043730	7.30216352771195	7.04538893937360
C	2.96760922936948	6.94566839685661	8.39394575482253
C	4.05375361967761	6.81649004494067	6.25402852589128
C	3.92351286326906	6.10081328692641	8.93244805205254
H	2.17563510059920	7.33269268454936	9.02409304710323
C	4.95170989797810	5.61645139713138	8.13612785453694
H	3.86665160488171	5.82907426066764	9.98050174515899

C 5.01648652695335 5.98078103983985 6.79904771917061

H 4.10857037627440 7.09122750671332 5.20888017408324

H 5.81892179305649 5.60783744889887 6.17239282690612

H 5.70487939201415 4.96059807866961 8.55794388346570

3.8 Transition-State of Configurational Inversion of 7 at P-Ph in Gas Phase

!Grid6 FinalGrid7 PBE0 def2-TZVP d3bj optts numfreq

Total enthalpy ... -2498.41755467 Eh

Total entropy correction ... -0.09905821 Eh -62.16 kcal/mol

Final Gibbs free enthalpy ... -2498.51661288 Eh

68

Coordinates from ORCA-job Input

P 5.00985148684332 4.38201250598389 13.17227882038468

C 4.21298666743295 4.19967499061516 14.79309757814461

P 5.34285104535168 6.45935420259634 13.66890582337721

C 6.36308861745800 3.29312467724023 13.70017221043982

C 3.19872113349338 4.97369027512444 15.36468068099170

C 4.76097112324461 3.09224301292712 15.46078653064078

C 3.86194881214940 7.12660541232952 14.31404659735021

C 6.79824995418390 7.44520758689608 13.43683698263582

C 7.60328255193699 7.27073494661391 12.30687245618771

C 5.96596119364746 2.61932673907514 14.80941816366484

Si 7.89683049920972 2.95818429496983 12.70076965928658

C 2.93767728392721 6.32540302070162 14.92774306069173

C 2.60031274240452 4.46405903928429 16.52659476444340

C 4.16072246955661 2.62098244442370 16.62384507399010

Si 3.41306615802275 8.93949674133919 14.02048492988464

C	7.15274195140549	8.41106023780386	14.38269358374507
H	6.57194576041357	8.50604327357104	15.29155244849746
C	8.72723688161608	8.05386579206663	12.12652458214135
H	7.33902613873981	6.52447326604213	11.56722178970297
H	6.51802987972487	1.77325124236822	15.21194730990099
C	7.49854625243294	3.09753897006310	10.87761299843849
C	8.55714676680438	1.25280403759198	13.09870791361067
C	9.17447124443082	4.24815544603620	13.17307598583538
H	1.99606746988160	6.76985806275079	15.23768006101221
C	3.05421373281284	3.29782309450971	17.12395819553842
H	1.80085207552880	5.03084804564107	16.99224789304034
H	4.55074323551338	1.74556769630604	17.13073385644575
C	4.16184810286754	9.56231558514535	12.42654137095429
C	3.94444857140244	10.01097822692457	15.46155557016263
C	1.54095065820333	8.99688221976772	13.88262114860006
C	8.25349657073724	9.22410692914098	14.16794195933153
C	9.04722646178841	9.04636389888823	13.04451886105557
H	9.35266987710279	7.89611323380776	11.25605668468435
H	8.39364737090794	2.96514837308835	10.26466142546890
H	7.07066837731645	4.07444607106744	10.63892497758648
H	6.77247871327732	2.33595921511677	10.58326812180396
H	7.83525754192614	0.47509253401767	12.83746886898713
H	8.79546977793554	1.16137544528466	14.16112805223491
H	9.47812113828653	1.06584124529469	12.54021695622387
C	9.06966788270420	4.96487376745588	14.36629038512049
C	10.27755863469058	4.49970274891729	12.35418589865786
H	2.56727625592614	2.93833061715164	18.02319530017505
H	3.81840531922498	10.58215406345956	12.23384665481193
H	3.85649337730003	8.93699794368631	11.58455974619906
H	5.25279582519958	9.56755691630892	12.46370482349882
H	5.03045137294312	10.11086982467564	15.49375518983880

H	3.61289396169732	9.59007649143502	16.41362502326577
H	3.52528701743033	11.01629568799475	15.36517353285283
C	0.89808916293053	8.31456537400703	12.84595897101032
C	0.74862138115736	9.70800425057990	14.78335535858321
H	8.50913342208535	9.98120601217337	14.90080578176631
H	9.92130104897989	9.66784501968214	12.89082664586093
H	8.21201219460861	4.80557574924784	15.01201591362213
C	10.02970516130221	5.89637272164621	14.73074030620367
C	11.24185848704627	5.42895599034550	12.71319639043027
H	10.38961899331042	3.96481236558184	11.41546193785766
H	1.48465901635371	7.73736056221345	12.13748295560337
C	-0.48001665531038	8.34477165394814	12.71098314591090
C	-0.63400601532528	9.74062550945918	14.65660742426636
H	1.21302116709940	10.24607079303555	15.60314280956519
H	9.91932857717386	6.45313247009564	15.65417432360376
C	11.11798242149700	6.12943806036313	13.90446115202720
H	12.08960691017825	5.61049921356087	12.06163385351816
H	-0.95709120457238	7.80729537789464	11.89905812620638
C	-1.25013340195662	9.05978240554257	13.61883432803785
H	-1.23019643882744	10.29814971025361	15.37049109402577
H	11.86514241207637	6.86385400213648	14.18341233013967
H	-2.32929257684201	9.08304266473244	13.51706665022375

3.9 Transition-State of Configurational Inversion of 7 at PC_4 in Gas Phase

!Grid6 FinalGrid7 PBE0 def2-TZVP d3bj optts numfreq

Total enthalpy ... -2498.43224795 Eh

Total entropy correction ... -0.10038288 Eh -62.99 kcal/mol

Final Gibbs free enthalpy ... -2498.53263083 Eh

68

Coordinates from ORCA-job Input

P	6.07831248508092	5.02423403435716	14.04891990884296
C	4.57538386858753	4.34970179115253	14.47977239401771
P	6.04559250266479	7.14769024544912	13.76677995254079
C	7.02019498382661	3.55544548486513	14.12959413715592
C	3.36782034559198	5.02028872743597	14.69284444110834
C	4.75745907934297	2.96016284766529	14.66884611185067
C	4.29020358097905	7.35117175902789	14.26372671942259
C	5.89962544823348	7.28287607814552	11.93926458451611
C	4.78648002210022	6.84207580944277	11.22861887818333
C	6.11533785246703	2.55871017361302	14.46059527764854
Si	8.83398203844196	3.40524227471798	13.74885216527902
C	3.31621781255788	6.46438923977286	14.57901606186927
C	2.28053623345203	4.24438982574492	15.06438651789487
C	3.62750899751279	2.21774350122133	15.04658956534252
Si	3.87569335215750	9.18871425704498	14.43498583689715
C	6.96442263153329	7.86062986042726	11.25580137133293
H	7.82744127757923	8.21486422235061	11.81114459097766
C	4.74116614589055	6.98177955235546	9.85211637910179
H	3.95806915672350	6.38776534188384	11.76105758397719
H	6.42705180363073	1.52370759051303	14.55033198278196
C	9.14824791178469	4.21552214808274	12.09108490114200
C	9.30523442564986	1.59732557874913	13.72121008433639
C	9.81042635682166	4.32030197471881	15.06444783418052
H	2.33927105836814	6.88893778737285	14.81160611030630
C	2.41514480016504	2.85687235891284	15.22642420635253
H	1.32024309705114	4.71739648166300	15.24265810353492
H	3.70705814669462	1.14794923862870	15.20941782029170
C	4.76873141415831	10.21705840907164	13.15097216866763
C	4.38431671602783	9.74675760769904	16.14595498465568

C	2.02667189063348	9.37272443157687	14.18988075431784
C	6.92360325874311	7.99076278220555	9.87464113060036
C	5.81031872251758	7.55496260839872	9.17328632346105
H	3.87171846885208	6.63837218608110	9.30287373428146
H	10.20603933883531	4.16049764224628	11.82194228364749
H	8.86319518879224	5.27110889376408	12.11204597671098
H	8.56115780903055	3.73283560198798	11.30605234871902
H	8.73571104362431	1.06982828213738	12.95229043393597
H	9.10559079211576	1.11461529830456	14.68085497669916
H	10.36713391407774	1.47421618370164	13.49410859287336
C	10.74913624549019	3.67826209058687	15.87192104989100
C	9.60685630442913	5.68820243391961	15.26464453365880
H	1.54783220447330	2.27639562140005	15.51834738327247
H	4.49796258785007	11.26742322896715	13.28969982928096
H	4.49852618838085	9.92663862160383	12.13406095707164
H	5.85413945008342	10.13447010056115	13.24645673457921
H	5.45782385592013	9.58983851538671	16.27943257462394
H	3.86785003880905	9.17500580863279	16.92022229913807
H	4.17710391351745	10.80916742051433	16.29896184817436
C	1.46209642542376	9.08767177302105	12.94339760160250
C	1.17395118720184	9.77787920928373	15.21637947825433
H	7.75858277925364	8.43846299002008	9.34805276945514
H	5.77294713947532	7.66111764040671	8.09518900239724
H	10.93192561889974	2.61584309247182	15.74719263379886
C	11.45922717955437	4.37203081739442	16.84232652545370
C	10.30977746690911	6.38742507274011	16.23226953757502
H	8.88054457057143	6.22776065231886	14.66251745028666
H	2.09935796282271	8.76194781844532	12.12605657322273
C	0.09831890288947	9.20401534171600	12.72949360846172
C	-0.19385400140431	9.89686458942604	15.00916560809709
H	1.58011733373684	10.00494475446198	16.19671830878861

H	12.18339381809319	3.85132088859636	17.45904433392856
C	11.24011427335103	5.72854923857033	17.02428398401411
H	10.12966317902606	7.44759755985450	16.37122539315048
H	-0.31949137612244	8.97783964374818	11.75482050781410
C	-0.73315637745484	9.60970684208959	13.76501274020595
H	-0.83919256097372	10.21346212204409	15.82095405284726
H	11.79143649825910	6.27240971201095	17.78304171537525
H	-1.80089478076341	9.70087828731785	13.60070373612443

3.10 Input Line for NMR Calculation of NICS(0) of 5

!Grid5 FinalGrid6 HF def2-TZVP AutoAux d3bj NMR

3.11 Input Line for NMR Calculation of Phosphoric Acid

!Grid7 NoFinalgrid PBE0 def2-TZVP d3bj CPCM(Chloroform) AutoAux NMR

3.12 Input Line for NMR Calculation of 7 (*S*,R)

!Grid7 NoFinalgrid PBE0 def2-TZVP d3bj CPCM(Chloroform) AutoAux NMR

3.13 Input Line for NMR Calculation of 7 (*S*,*S*)

!Grid7 NoFinalgrid PBE0 def2-TZVP d3bj CPCM(Chloroform) AutoAux NMR
